# Hydrolysis, polarity, and conformational impact of C-terminal partially fluorinated ethyl esters in peptide models

**DOI:** 10.3762/bjoc.13.241

**Published:** 2017-11-16

**Authors:** Vladimir Kubyshkin, Nediljko Budisa

**Affiliations:** 1 Biocatalysis group, Institute of Chemistry, Technical University of Berlin, Müller-Breslau-Strasse 10, Berlin 10623, Germany

**Keywords:** conformation, ester bond, hydrolysis, peptides, polarity

## Abstract

Fluorinated moieties are highly valuable to chemists due to the sensitive NMR detectability of the ^19^F nucleus. Fluorination of molecular scaffolds can also selectively influence a molecule’s polarity, conformational preferences and chemical reactivity, properties that can be exploited for various chemical applications. A powerful route for incorporating fluorine atoms in biomolecules is last-stage fluorination of peptide scaffolds. One of these methods involves esterification of the C-terminus of peptides using a diazomethane species. Here, we provide an investigation of the physicochemical consequences of peptide esterification with partially fluorinated ethyl groups. Derivatives of *N*-acetylproline are used to model the effects of fluorination on the lipophilicity, hydrolytic stability and on conformational properties. The conformational impact of the 2,2-difluoromethyl ester on several neutral and charged oligopeptides was also investigated. Our results demonstrate that partially fluorinated esters undergo variable hydrolysis in biologically relevant buffers. The hydrolytic stability can be tailored over a broad pH range by varying the number of fluorine atoms in the ester moiety or by introducing adjacent charges in the peptide sequence.

## Introduction

Fluorine is a rare element in natural biochemical settings [1]. Notwithstanding several prominent fluoro-organic metabolites in nature [2–3], fluorine is virtually absent from natural biopolymers such as proteins and nucleic acids. Therefore, organofluorine groups lack a natural background in spectroscopic observations of biological samples. This feature is especially beneficial for NMR applications because the sole stable fluorine isotope (^19^F) has the third largest magnetogyric ratio among the nuclei measured by NMR (after ^3^H and ^1^H hydrogen isotopes); as a result, ^19^F NMR experiments are remarkably sensitive [4].

Fluorine-containing groups can be incorporated into biopolymers by various approaches, including those that utilize biosynthesis [5–7], enzymatic conversion [8], chemical synthesis [9–10], and ligation reactions [11]. Depending on the research target, ^19^F NMR measurements can be used to study ligand–protein [12] and protein–protein interactions [13]; membrane proteins [14–16] and membrane-associated peptides [17–18]; equilibria among conformations of RNA [19], DNA [20], and peptide nucleic acids (PNA) [21]; and many others. Particularly recent is the development of peptide-based contrast agents for ^19^F imaging [22].

In polypeptides, the incorporation of fluorine can significantly alter the properties of the native molecule. The hydrophobicity [23–24], conformational equilibria [25], and the thermodynamic [26–29] and kinetic [30–31] folding profiles can be altered by the presence of even a single fluorinated amino acid in the sequence. Perhaps the most studied molecules exhibiting such effects are the proline analogues, with the proline-to-fluoroproline exchange providing the first proof-of-principle and experimental basis for a number of subsequent conceptual studies [32]. For example, these were used to demonstrate the impact of non-canonical amino acids in proteins [33]. Fluoroproline-containing sequences were also applied as collagen mimics to dissect collagen-stabilizing forces [34–35].

However, the impact of fluorine labeling on polypeptides is still not fully understood and it may appear controversial in the literature. For example, a donor–acceptor type enhancement of the face-to-face stacking of phenyl- and pentafluorophenyl groups has been suggested [36]; though, subsequent studies of α-helical [37], peptoid [38], and collagen-mimicking models [39] did not support this suggestion. Though, this was reported later in a context of a hydrophobic core of model protein structures [40]. Furthermore, the influence of the fluorinated groups on lipophilicity remains uncertain, especially in biochemical literature.

**Figure 1:**
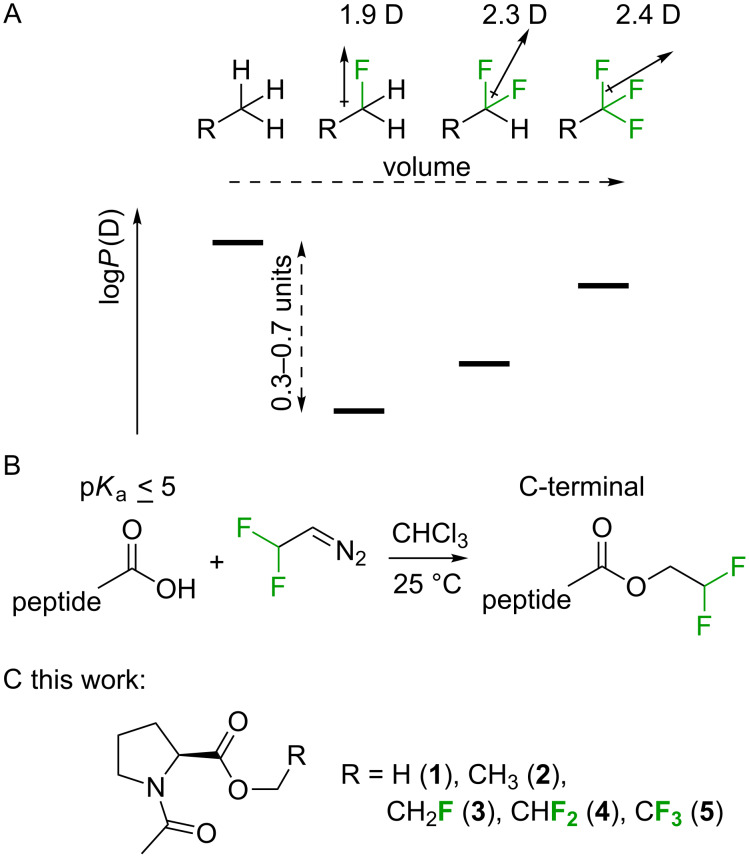
A. Dependence of the lipophilicity (log*P*) on the number of fluorine atoms in a partially fluorinated terminal alkyl group demonstrates a characteristic checkmark-shape [41]. B. Selective labeling of carboxylic acids (C-terminus in peptides) with 2,2-difluorodiazoethane yields 2,2-difluoroethyl esters [46]. C. Esters of *N*-acetylproline studied herein.

The impact of partially fluorinated alkyl groups on the polarity of small molecules was recently investigated in a series of model studies by Huchet and others [41–44]. These investigations demonstrated the checkmark-shape of the lipophilicity (log*P)* changes upon increasing the number of fluorine atoms in the terminal aliphatic alkyl fragment (Figure 1A; see also remark on page S2 in Supporting Information File 1). While a methyl group is lipophilic, the incorporation of one, two or three fluorine atoms produces a non-additive increase in the molecular dipole. On the other hand, the linear increase in the molecular volume due to the hydrogen-to-fluorine exchange increases the lipophilicity. As the result of these two opposite tendencies, the log*P* value decreases with incorporation of each moiety in the following order: RCH_3_ > RCF_3_ > RCHF_2_ ≥ RCH_2_F. The polarity and lipophilicity are among the most important effects of fluorination, as these parameters strongly impact other important biological properties, such as the potential distribution in biochemical compartments and the metabolic stability [44–45].

Considering the variety of spectroscopic applications, a labeling strategy that allows specific incorporation of a fluorinated moiety into a modular peptide fragment is highly desirable. Several recent efforts have been made to achieve this goal [9,47]. Fluorinated diazoalkanes have shown particularly promising results in small molecule functionalization [48], and these have a potential to be used for biomacromolecular functionalization as well [49]. As an example, we recently discovered the selective formation of 2,2-difluoroethyl esters upon treatment of small molecules and peptide substrates with 2,2-difluorodiazoethane (Figure 1B) [46]. The transformation was carried out in chloroform, which certainly limited the substrate scope. Nonetheless, due to the high acidity of the C-terminal carboxylic group [50], a number of C-terminally modified peptides were readily prepared by this method with full conversion. The availability of the C-terminal 2,2-difluoroethanol-esterified peptides also enables the investigation of the impact of partial fluorination on target peptides. Of particular importance are investigations of the hydrolytic stability of the partially fluorinated esters and the possible conformational impact on the peptide. Thus, we herein report results from studies of *N*-acetylproline esters, which were used as models for C-terminally modified peptides (Figure 1C). In addition, the conformational impact is examined using several oligopeptides.

## Results and Discussion

### Synthesis

**Scheme 1:**
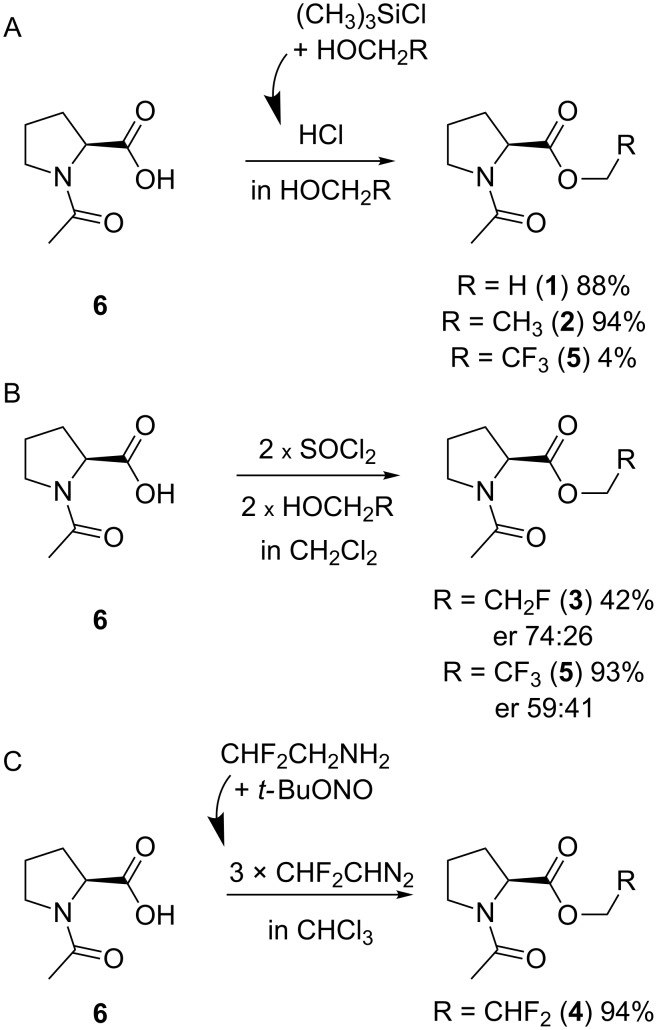
Synthesis of the model compounds.

The synthesis of the C-terminal esters was performed starting from *N*-acetylproline (**6**), which was prepared as described previously [50]. The reference methyl and ethyl esters **1** and **2** were prepared by stirring of **6** in acidic alcohol (methanol or ethanol, respectively) at room temperature overnight (Scheme 1A). We attempted to employ the same procedure for esterification of **6** in trifluoroethanol. However, this resulted in a very low yield of the desired product **5** (4%). The monofluoro- and trifluoroethyl esters **3** and **5** were then prepared via the corresponding chloranhydride, which was generated as described (Scheme 1B) [51]. The drawback of this method is that generation of chloranhydride from *N*-acetylated proline leads to partial epimerization of the residue.

Finally, ester **4** was prepared by treatment of **6** with 3 equivalents of the 2,2-difluorodiazoethane (Scheme 1C). The reaction was performed smoothly using chloroform as a solvent giving a good yield (94%). We also attempted to perform the reaction in an acetonitrile/water (1:1) mixture. However, only a very low yield (15%) was obtained with 2,2-difluorodiazoethane generated directly in the solvent mixture. Therefore, we tried generating 2,2-difluorodiazoethane in acetonitrile with subsequent addition of this solution to an aqueous solution of the substrate. Although the yield increased, it remained low (20%). The poor yields in the aqueous medium are explained by the high reactivity of the diazomethane species, which favors nonspecific reactions in water [52]. Very recently, more specific diazomethane reagents for water-tolerant esterification have also been developed [53]. During the revision of this paper Peng et al. reported on esterification of carboxylic acids using 2,2-difluorodiazoethane, which was performed in a number of aprotic organic solvents, including acetonitrile [54].

### Hydrolytic stability

The kinetic stability of the ester linkage in the aqueous medium is of critical importance, as it further defines the time window for the reactions with the corresponding esters under the biologically relevant conditions of aqueous buffers. Hydrolysis of the C-terminal esters has been fairly well described in the literature [55–57], indicating the hydrolysis occurs via a pseudo-first order reaction in a buffered medium assuming that [HO^−^] is constant (Equations 1–3):


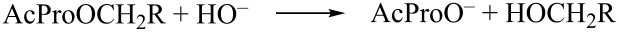


[1]
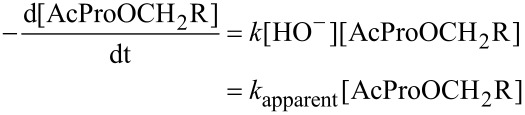


[2]
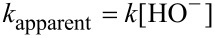


[3]
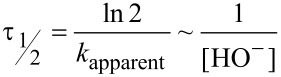


Particularly interesting is the dependence of the hydrolysis rate on the nature of the alkyl group of the ester; it has been demonstrated that ethyl esters hydrolyze approximately 2–3 times slower compared with the methyl esters [58], whereas methyl esters hydrolyze approximately 30 times slower compared with the 3’-tRNA esters in aminoacyl-tRNA [59]. Clearly, the hydrolysis rate depends on the electron donating/withdrawing effect of the alkyl moiety R, indicating a significantly compromised kinetic stability is likely for the partially fluorinated ester groups.

**Figure 2:**
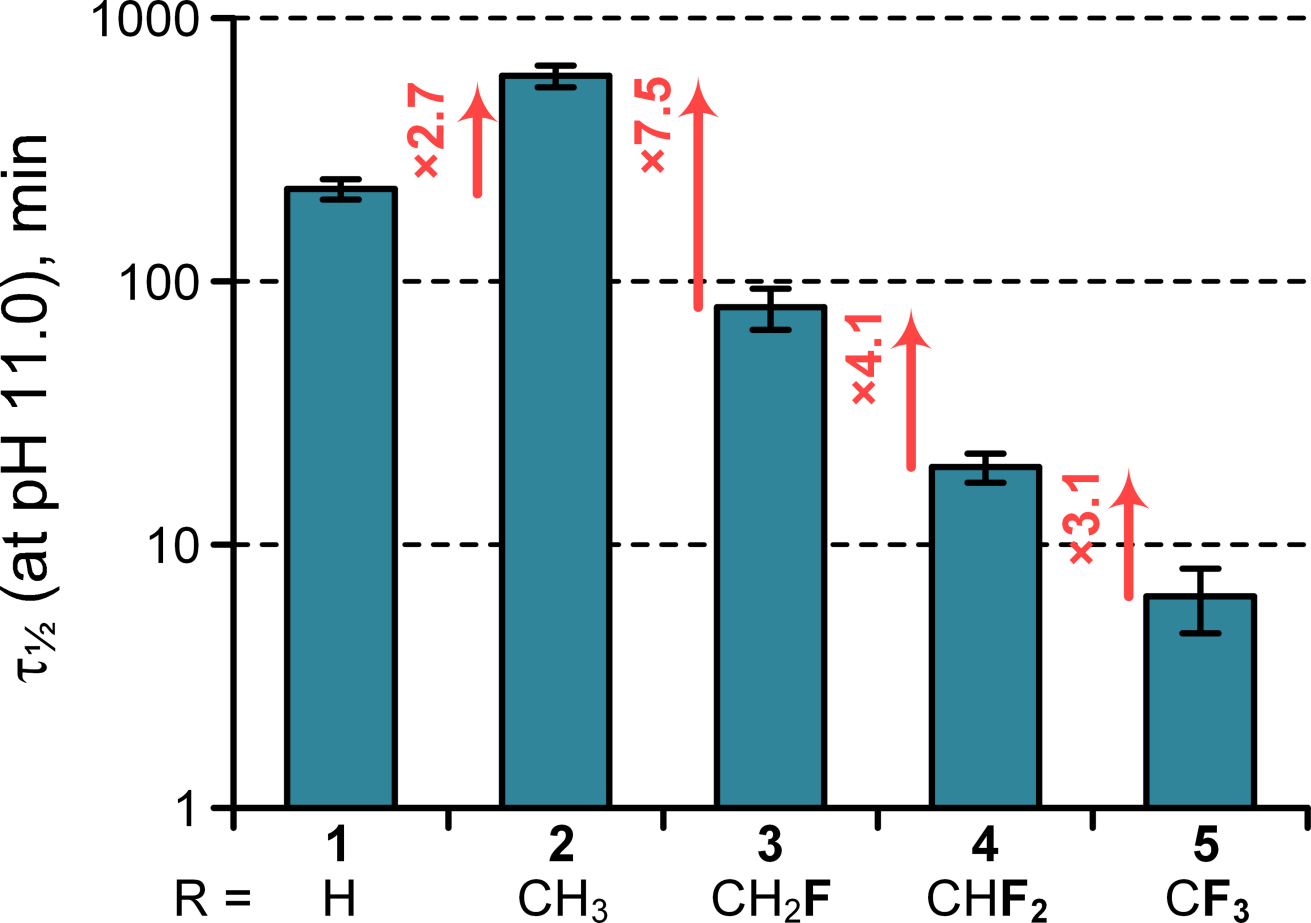
Kinetics of the C-terminal ester hydrolysis.

Next, we determined the half-life values following the above mentioned kinetic model for esters **1**–**5** in aqueous medium at pH 11 and 298 K (Figure 2). In accordance with previous reports, we found that the ethyl ester hydrolyzed 3 times slower compared with the methyl ester, whereas the introduction of the first fluorine atom dropped the half-life by a factor of approximately 8. For the subsequent fluorine atoms, the hydrolysis rate increased by a factor of 3–4 for each appended fluorine atom. The trifluoroethyl ester **5** delivered the fastest hydrolysis rate with a half-life of only 6.4 ± 1.7 min. Extrapolation to pH 8.0 gives a value of 107 ± 28 hours. (This procedure requires multiplication by 1000.) We experimentally determined the half-life of **5** at pH 8 to be 102 ± 2 hours, which agrees well with the extrapolation. The mutual consistency of the values determined with different kinetic modes (minutes at pH 11, days at pH 8) provides a good indication of their accuracy.

These data show that chemical modification with the terminally fluorinated ethyl esters **3**–**5** is possible over the course of days at physiologically relevant pH < 8. In fact, this stability enables further determination of several physicochemical properties for the examined compounds in water; for example, partitioning can be safely measured. It is also notable that the hydrolysis rate of the C-terminal trifluoroethyl or difluoroethyl esters may suggest a mechanism of self-cleavage for a potential drug molecule under physiological conditions, in addition to the most common path mediated by the esterases [60]. Though, the susceptibility of the fluorine-bearing esters towards natural esterases is still unknown, and systematic investigation of this issue is lacking in the literature. Notably, pH-programmed decomposition of fluoro-organic molecules has been recently reported for few other cases [61–62].

### Lipophilicity

**Figure 3:**
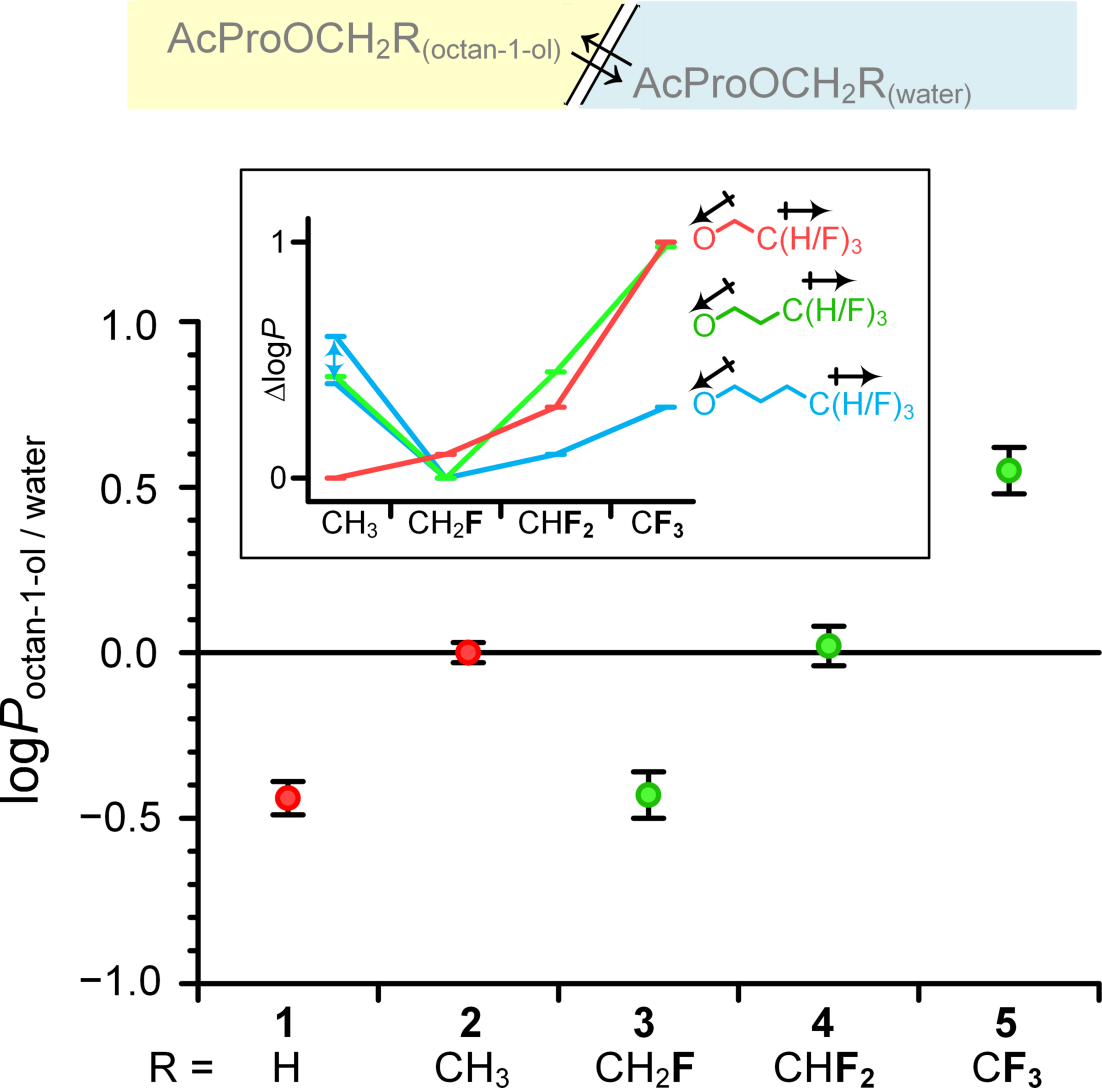
Partitioning of the esters **1**–**5** between octan-1-ol and water. Insert: comparison with the other partially fluorinated propoxy and methoxy groups from references [41,44].

To characterize the lipophilicity, esters **1**–**5** were subjected to 24 hours of partitioning between octan-1-ol and water at 298 K. The resulting partition is illustrated in Figure 3. In accordance with previous observations, the log*P* values exhibit the checkmark-shape: the lipophilicity decreased with the introduction of the first polar C–F bond, and with the introduction of subsequent fluorine atoms, the lipophilicity increased due to the increase in molecular volume. The lipophilicity of 2,2-difluoroethyl derivative **4** is nearly identical to that of parent ethyl ester **2**, and the relative difference in the log*P* from the increase (**5** vs **4**, Δlog*P* = +0.53 ± 0.13) and decrease (**3** vs **4**, Δlog*P* = −0.45 ± 0.13) in the number of fluorine atoms are equivalent to one methyl group difference (**2** vs **1**, Δlog*P* = +0.44 ± 0.08). The amplitude of the change is similar to that reported recently for the corresponding fluorinated ethanols [63]. Importantly, C-terminal esterification maintains a much higher lipophilicity compared with the C-terminal amide **7** (Table 1).

**Table 1:** Summarized properties of the analyzed compounds as determined by NMR at 298 K.

compound		τ_½_, min (aq buffer)	log*P* (octan-1-ol/water)

**1**	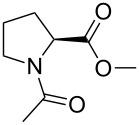	224 ± 20 (pH 11.0)	−0.44 ± 0.05
**2**	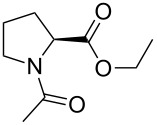	603 ± 58 (pH 11.0)	0.00 ± 0.03
**3**	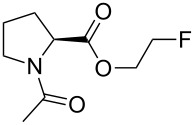	80 ± 14 (pH 11.0)	−0.43 ± 0.07
**4**	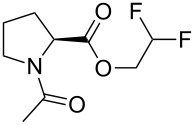	19.7 ± 2.5 (pH 11.0)	+0.02 ± 0.06
**5**	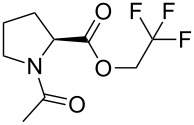	6.4 ± 1.7 (pH 11.0) 6112 ± 70 (pH 8.0)	+0.55 ± 0.07

**7**	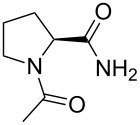	–	−1.57 ± 0.10

The observed checkmark shape differs from that previously reported for an isolated partially fluorinated methyl group at the end of a saturated aliphatic chain. In the latter case, the methyl group is the most lipophilic, more than a trifluoromethyl group; and the same has been reported for the partially fluorinated propyl ethers OCH_2_CH_2_CF*_n_*H_3−_*_n_* (log*P*: CH_3_ > CF_3_ > CHF_2_ ≥ CH_2_F) [41,44] (see insert on Figure 3). In contrast, among the partially fluorinated methyl ethers OCF*_n_*H_3−_*_n_*, the lipophilicity of the methoxy group is the lowest, while the fluoromethoxy group is the most lipophilic (log*P*: CF_3_ > CHF_2_ > CH_2_F ≈ CH_3_) [44,64]. These differences were explained by the mutual intramolecular compensation of the C–O dipole and the dipole from the partial fluorination [65]. Thus, the polar effect from the fluorine-bearing region becomes less prominent as the number of the C–C bonds to the polar fragments decreases. The partially fluorinated ethoxy moieties reported here represent an intermediate situation between the partially fluorinated methoxy and propoxy/higher alkoxy groups (log*P*: CF_3_ > CHF_2_ ≈ CH_3_ > CH_2_F).

### Amide isomerism

**Scheme 2:**
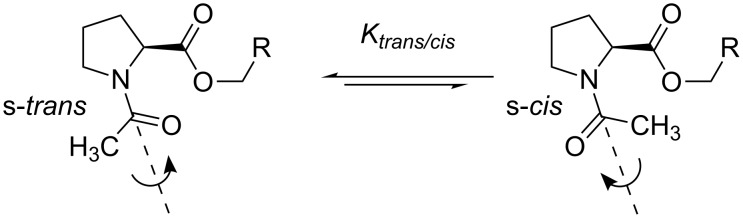
Amide isomerism in the *N*-acetylprolyl fragment.

The tertiary amide bond in *N*-acylprolyl can exist in two conformational states, s-*trans* and s-*cis* (Scheme 2), with the former being thermodynamically preferred in most cases [66–67]. The intrinsic contextual preference in the amide isomerism around the proline residue is usually characterized using small molecular models such as esters of *N*-acetylproline, similar to those in this study [35,68–69]. Within this model, the *trans*-amide prevalence is not large, and both rotameric states are readily observed in NMR spectra. In the *N*-acylprolyl models, the nature of the terminal groups can influence the amide isomerism. The most important factor is the presence of the C-terminal charge, which reduces the *trans*-amide stability [50,70]. Nonetheless, when the charge is eliminated, the influence of the C-terminal moiety may become negligible. For instance, we recently observed identical parameters for the amide rotation around the glycyl–prolyl amide bond in AcGlyGlyProGlyGlyNH_2_ [71] and AcGlyProOMe [68] compounds when measured in deuterium oxide.

**Figure 4:**
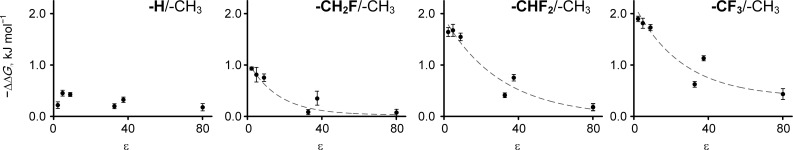
Enhancement of the *trans/cis* thermodynamic preferences in the ester models as a function of the solvent dielectric constant, ε. The *trans/cis* ratios were determined from the ^1^H and ^19^F NMR spectra at 298 K. Solvent set: C_6_D_6_, CDCl_3_, CD_2_Cl_2_, CD_3_OD, CD_3_CN, D_2_O. For details see Table S1 in Supporting Information File 1.

For **1**–**5**, we found the *trans/cis* ratios ≈ 5 for all five esters when measured in aqueous medium; the kinetic parameters of the amide rotation were also nearly identical (Table 2). We then noticed that the *trans/cis* ratios measured for the fluorine-labeled esters **3**–**5** in nonpolar solvents (such as benzene) were systematically higher relative to the reference compounds **1** and **2**. We tested the relative increase in the *trans*-amide preference ΔΔ*G* as a function of solvent (Equation 4) and found a dependence of this parameter on the dielectric constant, as would be expected from an electrostatic interaction between the polar alkoxy group and the amide moiety (Figure 4).

**Table 2:** Summarized conformational properties of the compounds **1**–**5** as measured by NMR.

	structure	amide isomerism						α-CH multiplicity:^a^ dd, *J*_HH_ = Hz			

			in D_2_O			in C_6_D_6_		in D_2_O		in C_6_D_6_	
						
		*K_trans/cis_*^a^	*k*∙10^3^, s^−1^ (310 K)^b^		*K_trans/cis_*^a^	k∙10^3^, s^−1^ (298 K)^b^					
					
			*trans*→ *cis*	*cis*→ *trans*		*trans*→ *cis*	*cis*→ *trans*	*trans*	*cis*	*trans*	*cis*

**1**	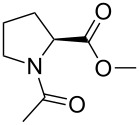	4.94 ± 0.05	7.0 ± 0.5^c^	33 ± 2^c^	5.10 ± 0.10	67 ± 2	342 ± 10	8.5, 4.7	8.7, 2.5	8.1, 3.7	8.6, 2.6
**2**	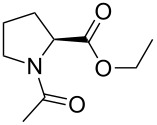	4.60 ± 0.08	6 ± 1	20 ± 5	4.67 ± 0.03	67 ± 2	309 ± 14	8.7, 4.5	8.8, 2.4	8.3, 3.6	8.5, 2.7
**3**	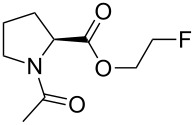	4.74 ± 0.04	7 ± 1	30 ± 2	6.80 ± 0.04	52 ± 2	348 ± 11	9.1, 4.6	8.9, 2.7	8.2, 4.2	8.4, 2.6
**4**	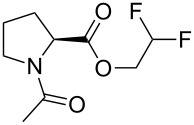	4.95 ± 0.05	7 ± 1	32 ± 4	9.06 ± 0.26	47 ± 2	430 ± 17	9.3, 4.7	8.8, 2.5	8.0, 3.9	8.7, 2.5
**5**	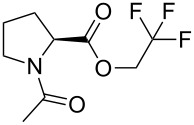	5.48 ± 0.14	6 ± 1	35 ± 5	10.04 ± 0.15	48 ± 2	477 ± 13	9.2, 4.7	8.8, 2.4	8.0, 4.1	8.7, 2.6

^a^Determined in ^1^H (and ^19^F) one-dimensional NMR spectra, analyte 50 ± 10 mM, 298 K; ^b^measured by ^1^H and ^19^F{^1^H} EXSY NMR at 50 ± 10 mM analyte concentration; ^c^as reported in [69].

[4]
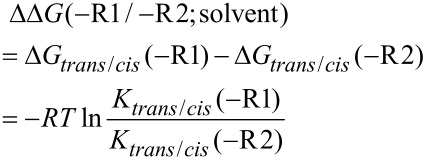


**Scheme 3:**
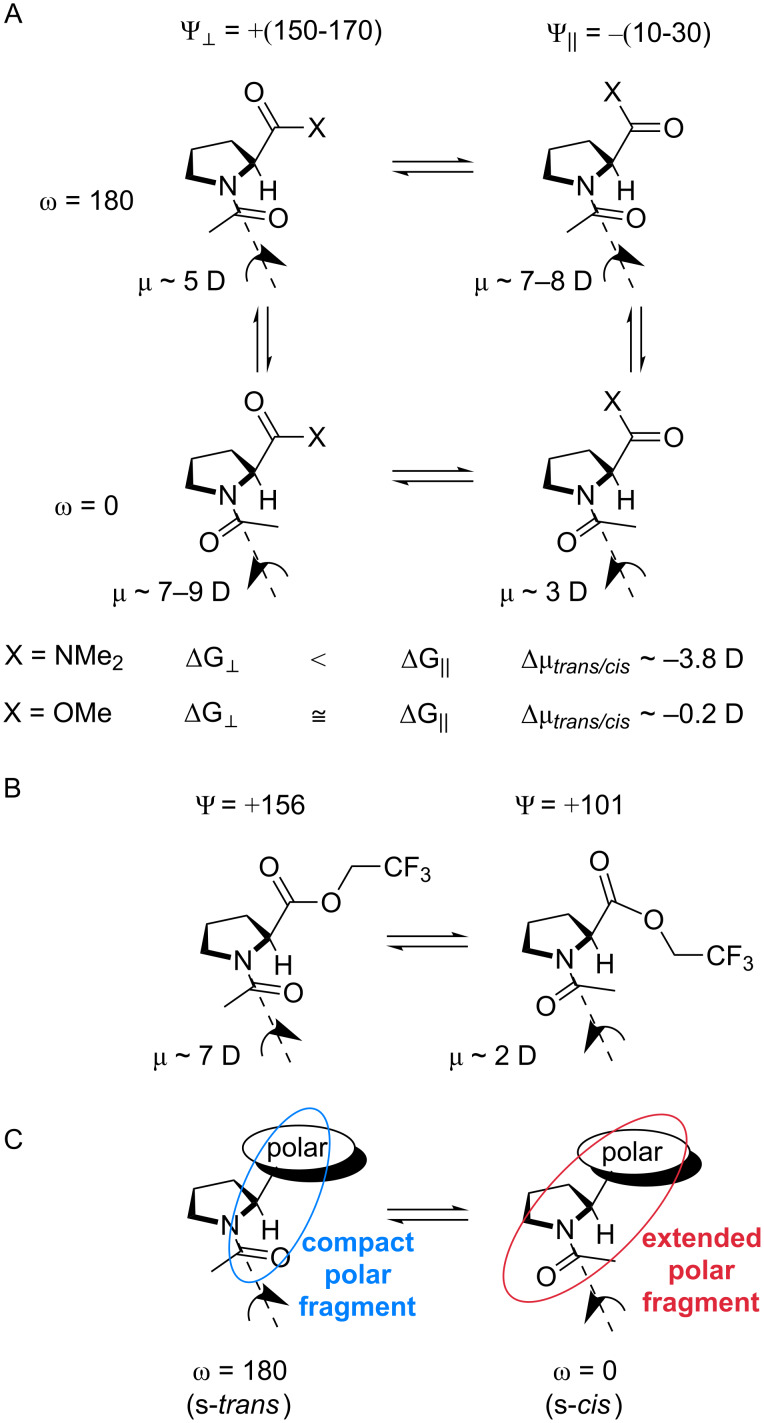
A. Four-state conformational equilibrium model used by Siebler et al. [72] for explanations of the elevated *trans/cis* ratio in nonpolar solvents, which occurs for X = N(CH_3_)_2_ and does not occur for X = OCH_3_. B. The two-state lowest energy model. C. The simplistic explanation of the C-terminal polarity effect observed for the partially fluorinated esters **3**–**5**.

A somewhat similar situation has been recently reported by Siebler et al. for the C-terminal amide in dimethylamido *N*-acetylproline, where the conformational equilibrium rendered the s-*cis* conformation remarkably more polar compared with the s-*trans* counterpart (Scheme 3A), which leads to an elevated *trans/cis* ratio in nonpolar solvents such as dioxane and chloroform [72]. In contrast to this situation, in methyl ester **1**, the dependence of the *trans/cis* ratio on the solvent is known to be marginal [73–74].

To explain the elevated *trans/cis* ratio observed with partially fluorinated esters **3**–**5**, we simulated the dipolar moment of compound **5** by DFT modelling. The dipolar moment for the lowest energy structure predicted a strong preference in favor of the less polar s-*cis* conformation (Ψ = +101, μ = 2.3 D) over the s-*trans* (Ψ = +157, μ = 5.8 D; Scheme 3B). Semi-empirical simulation of the four-state model (see Scheme 3A) also predicted a higher polarity for the s-*trans* conformational states (μ = || 4.6, **_┴_** 7.1 D) compared to the s-*cis* (μ = || 4.7, **_┴_** 3.7 D) counterpart due to the conformational contribution of the terminal fluorinated moiety. Both predictions clearly contradict the experimentally observed tendencies.

A possible problem with the existing models is that they assume a defined orientation for the fluorine-bearing moiety. However, this moiety may be rather flexible, similar to the parent esters **1** and **2**. This conclusion is suggested by the log*P* differences between esters **3**–**5**, which are nearly identical to those between the parent alcohols (mono, di- and trifluoroethanols) [63,75]. It is therefore apparent that in contrast to **1** and **2**, a more complex equilibrium should be considered for compounds **3**–**5** that involves at least three mutually orienting dipoles in a complex energy landscape. This may be very challenging for computational analysis since the amplitude of the effect is only ≤2 kJ mol^−1^. At this point, we can propose a simplistic perception of this complex interaction based on the fact that in the s-*trans* conformation, the amide carbonyl group is moved towards the carboxyalkyl moiety, which increases the interaction between the mutually orienting dipoles within a more compact structure (Scheme 3C). As the result, the gradual increase in the polarity from the most distant partially fluorinated moiety leads to an increase in the effect in the order **3** < **4** < **5** (Figure 4).

**Scheme 4:**
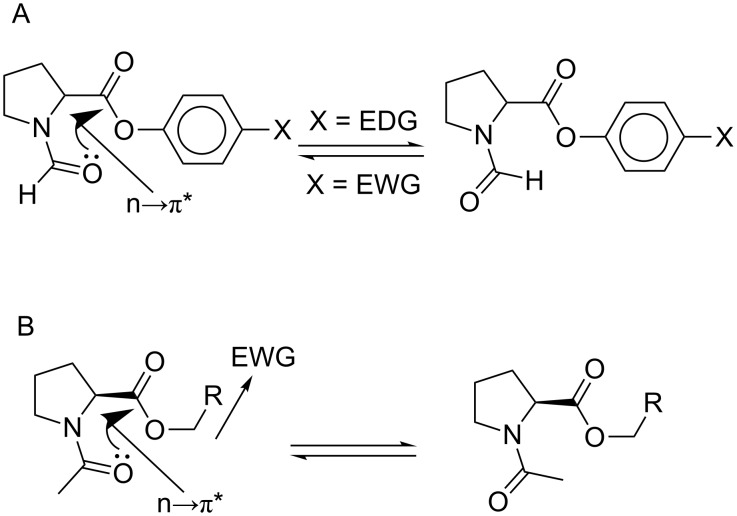
Elevation of the *trans/cis* ratio in derivatives of *N*-acyl proline may result from the enhanced n→π* interaction between the carbonyl-groups. EWG = electron-withdrawing group, EDG = electron-donating group.

An alternative explanation for the elevated *trans/cis* ratio is strengthening of the n→π* interaction between the carbonyl groups. For example, Hodges and Raines reported elevated *trans/cis* ratios in phenyl esters of *N*-formylproline containing electron withdrawing substituents (X in Scheme 4A) when measured in chloroform [76]. The *trans*-amide preference was enhanced by 2.2 kJ mol^−1^ for X = NO_2_ relative to X = N(CH_3_)_2_. Thus, it is possible that in the case of the esters **3**–**5**, the electron-withdrawing effect of fluorine atoms leads to a similar enhancement of the n→π* interaction between the carbonyl groups due to the higher electrophilicity of the carboxyl carbon atom (Scheme 4B). Nonetheless, this explanation does not explain why, despite the electrophilicity of the carboxyl group indicated by the hydrolysis experiments (Figure 2), the exchange of the ethyl group by the methyl group enhances the hydrolysis rate while only marginally impacts the *trans/cis* ratio. Furthermore, it is unclear why a polar solvent quenches the enhancement of the electrophilicity of the C-terminal carbonyl, which requires the differential solvation of two rotamers. This differential solvation may be explained by the polarity of the two rotamers; thus, the polarity model expressed in Scheme 3 cannot be dismissed.

Additional factors, such as multipolar interactions [77] and especially the polar surface exposure [78], may also be considered. Further understanding of the relevant interactions requires more detailed studies and experimental models. Nonetheless, the experimental observations and the simple description proposed herein (Scheme 3C) suggest that the amide bond of the *N*-acetylproline fragment can function as an intramolecular probe of the C-terminal ester group polarity. Notably, the predicted flexibility of the partially fluorinated alkoxy groups in the presence of other strong dipoles is inconsistent with the concept of conformational adaptors elaborated by Müller et al. quite recently [44,65].

### Side chain conformation

The side-chain conformations of proline are restricted by the pyrrolidine ring structure. The two main envelope conformations are *exo*- and *endo*- (alternatively designated as *up*-/*down*-, respectively), in which by the C^4^-ring atom is oriented toward or away from the carboxyl group orientation, respectively [79–80]. This conformational equilibrium manifests in the χ angles, which is reflected by the *J*-coupling observed between the α-CH and β-CH_2_ groups in the ^1^H NMR spectra (*J*_αβ_) [81–82]. A recent analysis of the *J*_αβ_ values by Braga et al. was used to quantify the pucker equilibrium in compound **1** [74]. We examined the values of *J*_αβ_ for compounds **1–5** in a polar (deuterium oxide) and nonpolar (benzene-*d*_6_) solvents. The observed *J*_αβ_ values are consistent with those reported previously [74,83], and the value differences likely reflect a change of only a few percent due to the contribution from the two conformations. This result indicates that the C-terminal partially fluorinated ester has a negligible influence on the side chain conformation of the prolyl moiety.

#### Conformation of short oligopeptides

We then examined how the properties observed this far impact the conformational properties of a peptide chain. As described above, the C-terminal charge has a large effect on the stability of the *trans*-amide alignment of the peptide bond. It has been demonstrated that removing the terminal charge enhances the stability of the polyproline-II helical fold in collagen-mimicking [84] or polyproline sequences [70] and significantly shifts the equilibrium of short model sequences in favor of the polyproline-II over the β-structure conformation [85]. Reaction with 2,2-difluorodiazoethane esterifies and thus eliminates the charge of the C-terminal carboxyl group, thereby enhancing the polyproline-II stability. This effect contrasts with the effects of other terminal modifications, such as aromatic amino acids, which reduce the polyproline-II fold stability [86].

**Scheme 5:**
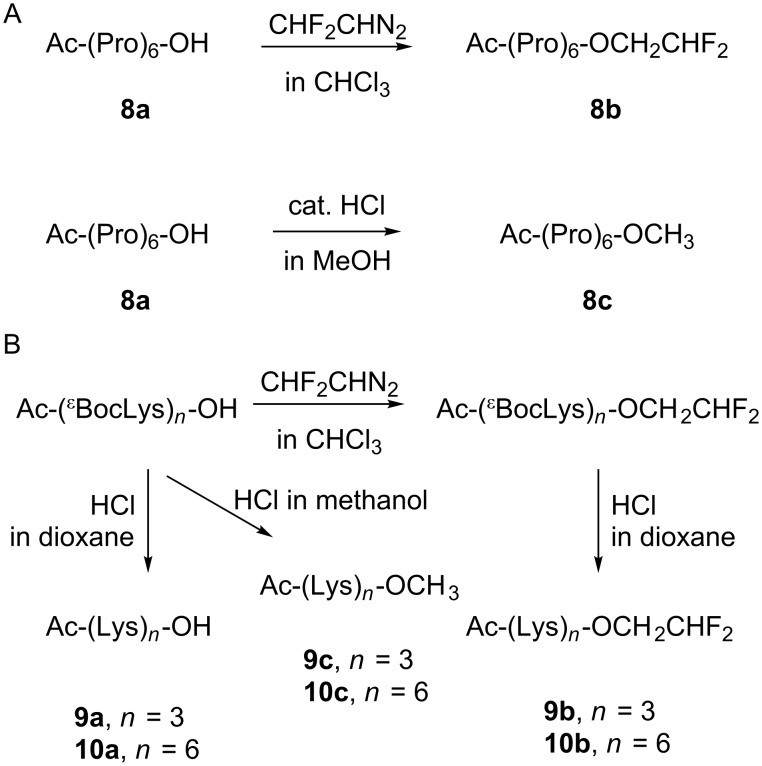
Synthesis of the model peptides.

**Figure 5:**
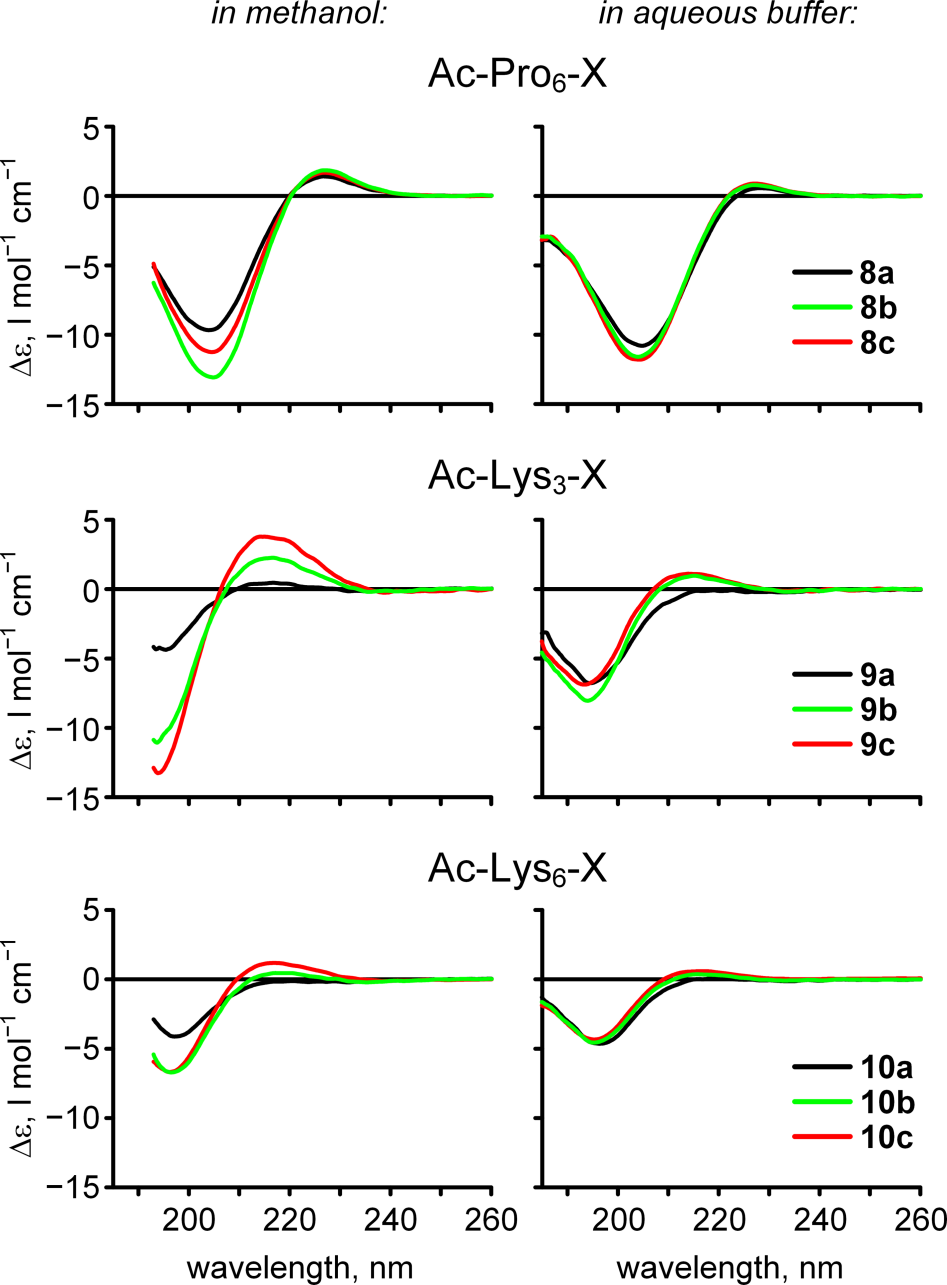
Mean residue molar circular dichroism (Δε) of peptides **8**–**10** in methanol (left) and aqueous phosphate buffer (right, 50 mM buffer, pH 7.0) samples. Recorded at 298 K and 100 μM peptide.

We then prepared hexaproline peptide **8a**, which was subsequently esterified with the diazomethane reagent to give **8b**. Methyl ester **8c** was also prepared for comparison (Scheme 5A). Measured circular dichroism (CD) spectra for the hexapeptides **8a**–**c** (Figure 5, top) demonstrate strong negative bands at 204 nm and weak positive bands at 227 nm, which indicate a polyproline-II fold. Remarkably, this spectral shape was observed for all three hexaproline peptides **8a**–**c**, while some increase of the negative band amplitude was only observed for the methanol samples. Overall, this model suggests a small conformational impact from the C-terminal 2,2-difluoroethylation or methylation as compared to the parent peptide **8a**.

These findings motivated assessment of a model system with a more dynamic structure. For example, it has been shown that oligolysine sequences favor a polyproline-II fold in short stretches [87]. We prepared tri- and hexalysine peptides without (**9a**, **10a**) and with (**9b**, **10b**) the C-terminal difluoroethyl ester, as well as the methyl esters (**9c**, **10c**) as shown in Scheme 5B. The CD spectra of these peptides (Figure 5) revealed a negative band at ≈195 nm and a band at ≈216 nm, thus enabling measurement of the conformational impact. This band was clearly positive for the esterified peptides **9b**,**c** and **10b**,**c** in contrast to the parent non-esterified peptides **9a** and **10a**. This is especially seen in the shortest trilysine sequence, where the esterification significantly increases the charge density, and thus the extended polyproline-II helix can be stabilized via the charge repulsion forces. Longer hexalysine sequences exhibited the same effect, albeit to a lower extent. Predictably, the C-terminal charge has a decreasing effect as the length of the peptide increases.

**Figure 6:**
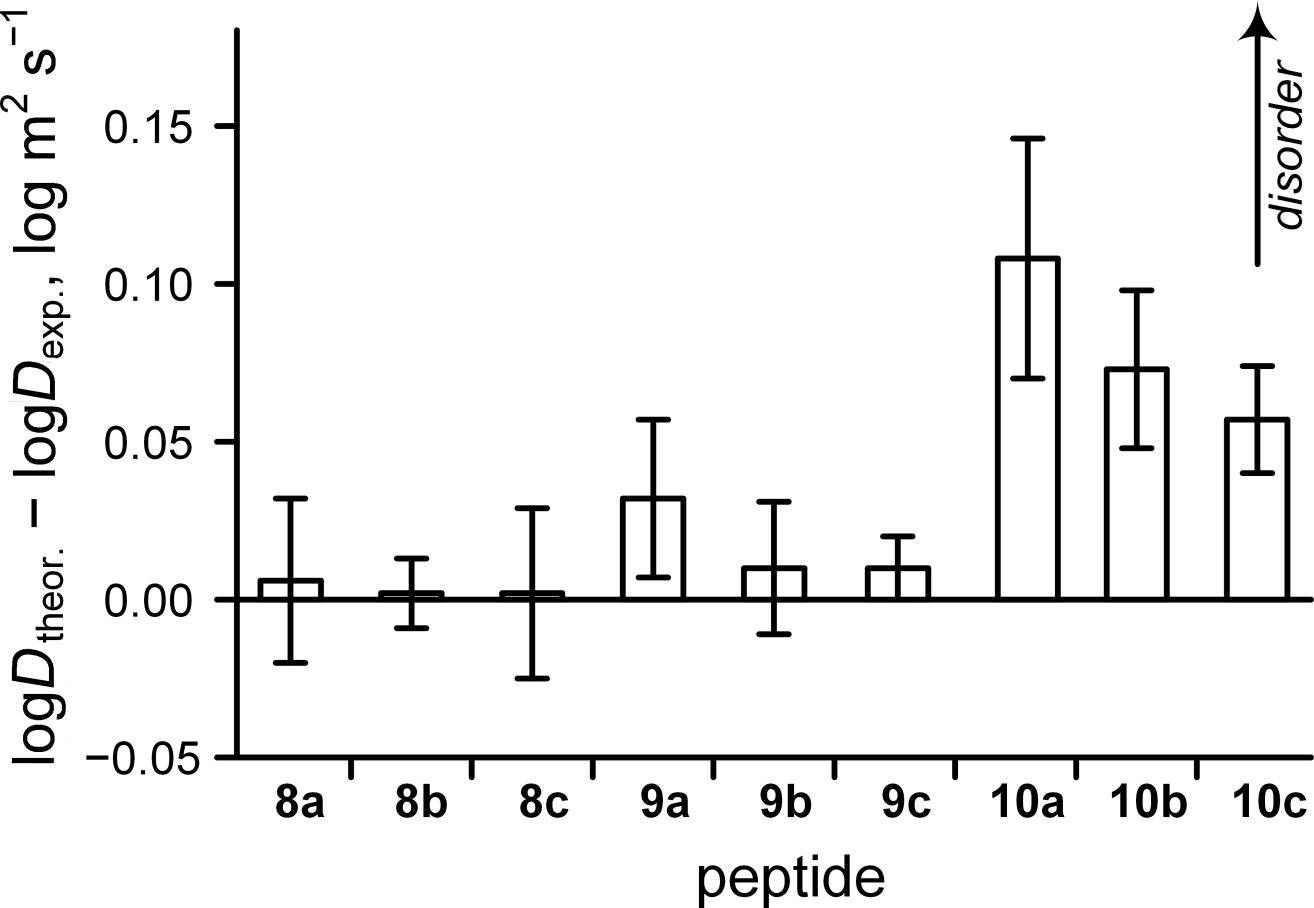
Conformational analysis of the peptides by ^1^H DOSY NMR (D_2_O, 298 K). The theoretical values were calculated according to Equation 5 assuming deuterium oxide viscosity and 298 K.

An independent refinement of these conclusions can be found in diffusion measurements conducted by ^1^H DOSY (diffusion ordered spectroscopy). We previously applied a simple set of equations for estimation of the diffusion coefficients of an oligomeric hydrophobic polyproline-II helix [71]. Here, we combined these equations in order to derive Equation 5, which describes diffusion as a function of molecular weight assuming pure deuterium oxide solution at 298 K. This equation considers molecules to be rigid spheres, as it is based on the Stokes–Einstein relationship. The ‘coil’ state significantly contributes to the conformational flexibility, which results in a diffusion deceleration [88]. We used the simple criterion of the difference between the experimental log*D* and the ‘theoretical’ log*D* derived from Equation 5 (MW – molecular weight in Da, log*D* in log m^2^ s^−1^) (Figure 6). The increase in these values indicates a ‘coil’ contribution in the molecular conformation. However, it should be kept in mind that the diffusion data describes the overall disorder of the peptide body, including the side chain conformations; in contrast, CD represents only the backbone.

[5]
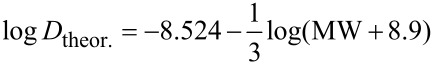


The diffusion data (Figure 6) indicated that the hexaproline peptides **8a**–**c** are rather rigid, the trilysine peptides **9a**–**c** are somewhat more flexible, and the hexalysine peptides **10a**–**c** are characterized by remarkable molecular disorder. In all cases, the C-terminal esterification reduced the level of disorder, although, the effect is hardly distinguishable from experimental error values. Overall, these results demonstrate that the side-chain disorder may play a more significant role in the peptide diffusion properties, than the backbone rigidity induced by an eliminated terminal charge.

The conformational impact of the C-terminal esterification described here is also expected to be generic, as the polyproline-II structure is preferred by various oligopeptide sequences [89–90] and is highly abundant in the unfolded protein states [91].

#### Hydrolytic stability in peptides

**Figure 7:**
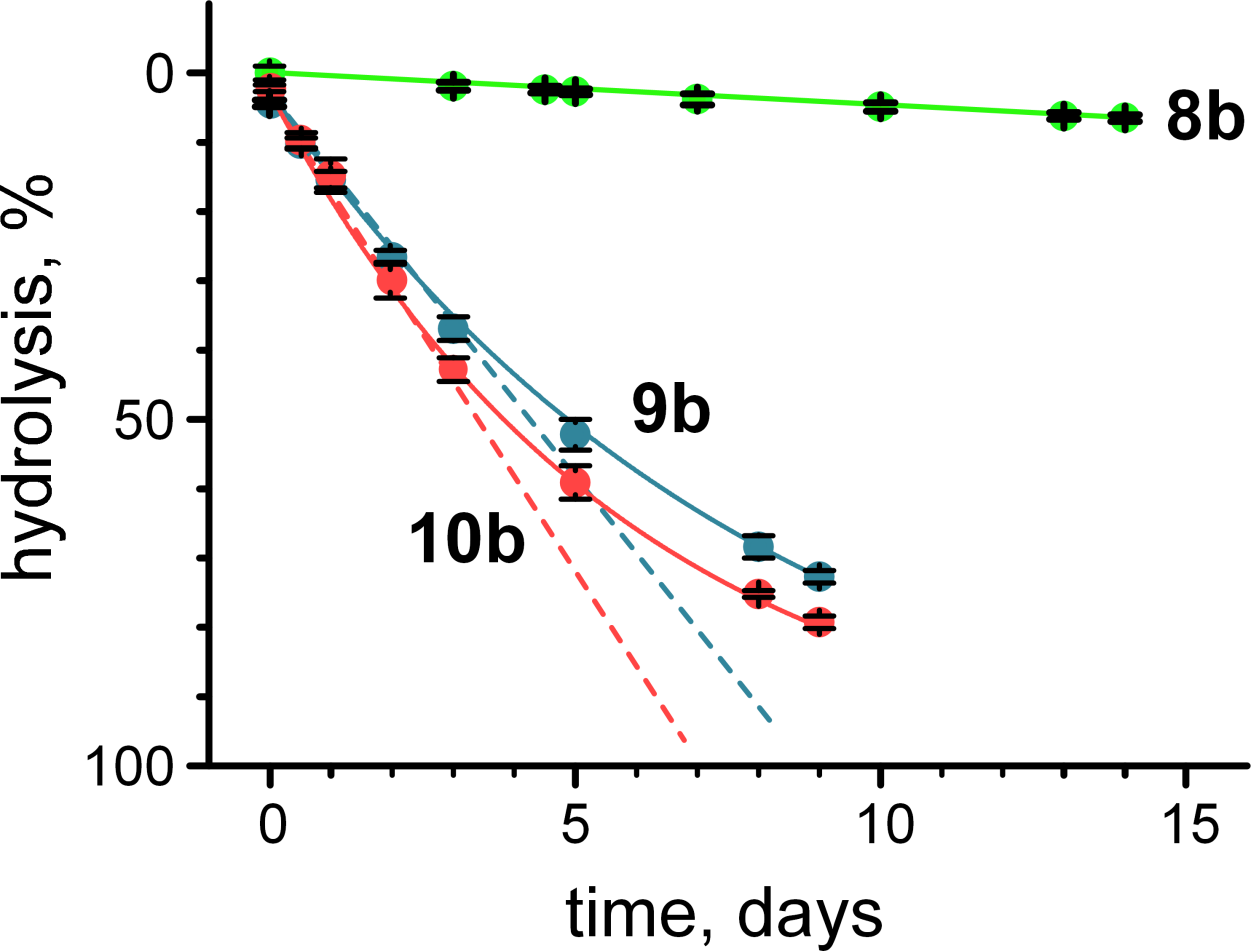
Hydrolysis of the C-terminal 2,2-difluoroethyl esters of the oligopeptides in buffered deuterium oxide at 298 K and pH 7. Dashed lines reflect the pseudo zero-order kinetic behavior of hydrolysis during the initial stages of this process.

Finally, we tested hydrolytic stability in esterified peptides **8b**, **9b** and **10b** by observing hydrolysis of the ester using ^19^F NMR in buffered deuterium oxide (pH 7). We expected pseudo-first order kinetics as for the hydrolysis of esters **1**–**5** at pH 11. Nonetheless, for the peptide esters **8b**, **9b** and **10b**, the experimental decay (Figure 7) of the ester concentration resembled pseudo-zero order kinetics, which was also observed for another amino acid ester hydrolysis not shown here.

Both fittings to the zero (Equations 6 and 7) and first order (Equations 1–3) kinetic models demonstrated fairly good agreement with each other (see Experimental).

[6]
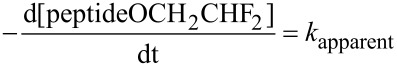


[7]
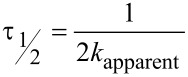


Notably, the half-life (Equation 7) of oligoproline **8b** (152 ± 14 days) demonstrated a good agreement with the half-life of monoproline peptide **4** extrapolated to pH 7.0 (137 ± 17 days). The half-life values for the oligolysine peptides **9b** and **10b** were much shorter at 5.0 ± 0.5 and 4.0 ± 0.3 days, respectively. These results can be explained by the increased local concentration of negatively charged hydroxide ions resulting from the positive charge, which causes ester hydrolysis. The accelerated hydrolysis of oligolysine esters can potentially lead to partial 2,2-difluoroethyl ester hydrolysis during the CD sample handling, and thus explain somewhat reduces CD intensity compared to the methyl ester samples (Figure 5).

This result also suggests that the hydrolysis rate of a C-terminal ester in peptides can be well tailored by installing proximal charges in the molecule. This conclusion is of particular importance due to growing interest in modifying potential peptide therapeutics with positively charged residues for improved cell permeability [92]. Though, the charge-induced hydrolysis acceleration from the proximal lysine charge (**9b**, **10b** vs **8b**, factor ≈ 30) is lower than the α-NH_2_/NH_3_^+^ hydrolysis acceleration in the classical studies of α-amino acid esters (factor ≈ 100–150) [58]. The hydrolysis rate can thus be further tuned by changing the proximity of the positive charge to the ester group. Further investigations of the peptide ester hydrolysis catalyzed by the proximal charges may be very beneficial for understanding of the role of ribosome electrostatics during translation termination [93–94], which is an ester hydrolysis reaction performed by bulk water [95].

## Conclusion

We have described the properties of C-terminal partially fluorinated ethyl esters of *N*-acetylproline. The hydrolytic stability decreases up to two orders of magnitude as the number of fluorine atoms in the ester group increases. Furthermore, the measured lipophilicity values follow the characteristic checkmark-shape, while the side chain conformation was not affected. The amide isomerism remained unchanged in polar aqueous medium. Conversely, in the nonpolar solvents, the equilibrium between the two conformers was shifted toward a more compact arrangement of the polar groups in the s-*trans* rotamer.

In oligopeptides, the C-terminal 2,2-difluoroethylation increased the polyproline-II structural contribution. The effect was seen most prominently in a short oligolysine peptide. The hydrolytic stability of the ester bond in the peptide depends on the charge of the peptide, as it was impaired in the positively charged oligolysine peptides compared with the neutral oligoproline. These results suggest the potential application of late-stage C-terminal esterification with partially fluorinated groups as a tool in peptide and therapeutic design, as well as in ^19^F NMR applications.

## Experimental

### Synthesis of model compounds

#### Esterification in acidic alcohols

*N*-Acetylproline (150 mg, 0.95 mmol) was dissolved in absolute alcohol (methanol, ethanol or 2,2,2-trifluoroethanol, 2 mL) and trimethylsilyl chloride (0.1 mL, 0.8 mmol) was added. The mixture was stirred for 21 hours at room temperature, solvent was removed under reduced pressure, and the crude material was purified on a short silica gel (≈11 g) column using an ethyl acetate/methanol 39:1 as the eluent. 143 mg of **1** was obtained as a clear oil (yield 88%), *R*_f_ = 0.53 (ethyl acetate/methanol 39:1). 167 mg of **2** was obtained as a clear oil (yield 94%), *R*_f_ = 0.61 (ethyl acetate/methanol 39:1). 10 mg of **5** was obtained as a clear oil (yield 4%), *R*_f_ = 0.74 (ethyl acetate/methanol 39:1).

#### Esterification via chloranhydrides

Compound **3**: *N*-acetylproline (101 mg, 0.64 mmol) was dissolved in anhydrous dichloromethane (3 mL) and the resulting solution was cooled down in an ice bath. Thionyl chloride (0.1 mL, 1.38 mmol, 2.2 equiv) was added, followed by 2-fluoroethanol (80 μl, 1.38 mmol, 2.2 equiv). The mixture was stirred at ambient temperature for 14 hours. The solvent was removed under reduced pressure, and the resulting crude material was subjected to a silica gel (20 g) column purification using an ethyl acetate/methanol 39:1 eluent. 55 mg of **3** was obtained as a clear oil (yield 42%), *R*_f_ = 0.63 (ethyl acetate/methanol 39:1).

Compound **5** was obtained in an analogous procedure to **3** starting from *N*-acetylproline (100 mg, 0.64 mmol), thionyl chloride (0.1 mL, 1.4 mmol, 2.2 equiv) and trifluoroethanol (0.12 mL, 1.6 mmol, 2.5 equiv). 141 mg of the product was obtained as a clear oil (yield 93%), *R*_f_ = 0.73 (ethyl acetate/methanol 39:1).

#### Esterification with diazoalkanes

No special precautions were applied when working with 2,2-difluorodiazoethane except for performing all work in a fume hood and maintaining conventional lab protection.

The procedure in chloroform involved mixing 2,2-difluoroethylamine (160 mg, 1.97 mmol, 3 equiv) in chloroform (5 mL), *tert*-butyl nitrite (90% purity, 260 mg, 2.27 mmol, 3.5 equiv) and acetic acid (2.5 μL, 0.044 mmol, 7 mol %). The resulting yellowish mixture was refluxed for 10 min. The reflux was stopped, and the mixture was allowed to stand at room temperature for 2 min. Then, a solution of *N*-acetylproline (100 mg, 0.64 mmol) in chloroform (1.5 mL) was added. The mixture was stirred at room temperature for the next 12 hours. Volatiles were removed under reduced pressure; the crude material was purified on a short (21 g) silica gel column using ethyl acetate/methanol 39:1 mixture as the eluent. 132 mg of **4** was obtained as a clear oil (yield 94%), *R*_f_ = 0.69 (ethyl acetate/methanol 39:1).

The procedure in acetonitrile/water was performed using same proportions as those in the chloroform reaction. The amine and nitrite were mixed in acetonitrile (2.5 mL), followed by the addition of *N*-acetylproline (2 mg) for activation. The mixture was refluxed for 10 min, and after the heating was stopped, this solution was added to a solution of *N*-acetylproline in water (2.5 mL). The mixture was stirred at room temperature for 16 hours. Then, trifluoroacetic acid (0.1 mL) was added, and the mixture was stirred for an additional hour to quench residual diazo compound. The solution was then freeze-dried. Purification of the crude material on a silica gel column afforded 30 mg of **4** as a clear oil (yield 20%). When the diazo compound was generated in an acetonitrile/water (1:1) mixture (4.5 mL), no heating was applied, and *N*-acetylproline was added to the yellowish mixture 5 min after mixing the amine with the nitrite. From this run, 21 mg of **4** was isolated after silica gel purification (yield 15%).

### Physical chemistry

#### Hydrolytic stability

Aqueous potassium phosphate buffer (100 mM) at pH 11.0 or 8.0 was mixed with 1:10 (v/v) deuterium oxide to give 550 μL of 91 mM buffer. The analyte **1**–**5** was added from a 200 mM stock solution in deuterium oxide to give 0.5 mM analyte. The first NMR spectra were collected within 4 min following addition of the analyte. The monitoring was continued by recording ^1^H and ^19^F NMR spectra at regular time points until >50% hydrolysis. Ambient temperature was maintained at 298 ± 1 K. The decay of the integral intensity of the analyte was quantified by integration. Plotting the logarithm of the intensity versus time delivered the kinetic constant, which was converted to the half-life time (squared correlation coefficient > 0.90). The experiments were performed in triplicate.

#### Partitioning

To 4–5 mg analyte, octan-1-ol (1.00 mL) and deionized water (1.00 mL) were added. The mixture was shaken gently at ambient temperature (298 ± 2 K) for 24 hours. Each phase (200 μL) was sampled using identical NMR tubes, and acetonitrile-*d*_3_ (300 μL) was added to each sample. The NMR measurements were performed by ^1^H or ^19^F detection at 298 K using calibrated 90-degree pulses and one-scan experiments in order to ensure complete pre-relaxation. The same acquisition parameters were used for the octan-1-ol and water samples; for processing, only the zero phase was adjusted. The spectra were baseline corrected, and equivalent resonances were integrated. The ratio between the signal intensities was considered as the partitioning constant. The partitioning experiments were performed in triplicate. Subsequent addition of water and octan-1-ol was performed in forward and reverse manner, and the resulting partitioning constants were identical within experimental error.

#### Conformational analysis

The amide rotational thermodynamic and kinetic measurements were performed by NMR as described in [50,68]. The equilibrium ratio between the rotameric states was obtained from the ^1^H, ^19^F and ^19^F{^1^H} (inverse-gated decoupling) spectra at 298 K by integration. The kinetics was measured in ^1^H and/or ^19^F{^1^H} z-cross-relaxation experiments at either 310 K (for deuterium oxide samples) or 298 K (for benzene samples). The *J*_αβ_-coupling values were obtained by visual inspection of the α-CH resonances in the ^1^H NMR spectra, and the accuracy was defined by the length of the time domain spectrum at approximately 0.3 Hz. The semi-empirical calculations were performed by using the PM6 algorithm from the MOPAC package. DFT geometry optimization of the **5** amide rotamers was performed using B88-PW91 GGA with the DZVP basis set.

#### Characterization of model compounds

^1^H and ^13^C NMR spectra were assigned using ^1^H{^19^F} inverse-gated decoupled, ^1^H{^13^C} dept45, ^1^H{^13^C} HSQC, ^1^H-^13^C HMBC, ^1^H NOESY/EXSY and ^19^F{^1^H} HOESY experiments. For the minor s-*cis* conformation, only chemical shifts and, when possible, multiplicities are given. The enantiomeric ratio (er) was measured in ^19^F{^1^H} inverse-gated decoupled NMR spectra of the dichloromethane-*d*_2_ solutions containing 40 mM analyte (550 μL) recorded upon addition of an equivalent amount of europium(III) tris-[3-(heptafluoropropylhydroxymethylene)-D-camphorate] in the same solvent (200 μL) as the shifting reagent. The spectra were measured at 298 K.

**Methyl *N*-acetylprolinate (1): **^1^H NMR (D_2_O, 700 MHz) δ s-*trans*, 4.37 (dd, *J*_HH_ = 8.5, 4.7 Hz, α-CH), 3.69 (s, 3H, OCH_3_), 3.60 and 3.56 (two m, 2H, δ-CH_2_), 2.23 and 1.95 (two m, β-CH_2_), 2.05 (s, 3H, CH_3_C=O), 1.94 (m, 2H, γ-CH_2_); s-*cis*, 4.63 (dd, *J*_HH_ = 8.7, 2.5 Hz, α-CH), 3.73 (s, OCH_3_), 3.47 (ddd, *J*_HH_ = 11.6, 8.5, 3.5 Hz, δ-CH), 3.41 (dt, *J*_HH_ = 11.4, 8.4 Hz, δ-CH), 2.27 and 2.15 (two m, β-CH_2_), 1.93 (s, CH_3_C=O), 1.89 and 1.79 (two m, γ-CH_2_); ^13^C{^1^H} NMR (D_2_O, 126 MHz) δ s-*trans*, 175.0 (s, CO_2_), 173.0 (s, N-C=O), 59.0 (s, α-CH), 52.9 (s, OCH_3_), 48.4 (s, δ-CH_2_), 29.2 (s, β-CH_2_), 24.2 (s, γ-CH_2_), 21.2 (s, CH_3_); s-*cis*, 174.6 (s, CO_2_), 173.4 (s, N-C=O), 60.7 (s, α-CH), 53.2 (s, OCH_3_), 46.6 (s, δ-CH_2_), 30.6 (s, β-CH_2_), 22.3 (s, γ-CH_2_), 21.2 (s, CH_3_); HRMS (ESI-orbitrap): [M + H]^+^ calcd for C_8_H_14_NO_3_, 172.0968; found, 172.0968; [α]_D_^25^ −83 (*c* 2.0, CHCl_3_).

**Ethyl *N*-acetylprolinate (2): **^1^H NMR (D_2_O, 700 MHz) δ s-*trans*, 4.34 (dd, *J*_HH_ = 8.7, 4.5 Hz, α-CH), 4.15 (m, 2H, OCH_2_), 3.59 and 3.56 (two m, 2H, δ-CH_2_), 2.24 and 1.95 (two m, β-CH_2_), 2.04 (s, 3H, CH_3_C=O), 1.94 (m, 2H, γ-CH_2_), 1.19 (t, *J*_HH_ = 7.2 Hz, 3H, CH_3_); s-*cis*, 4.61 (dd, *J*_HH_ = 8.8, 2.4 Hz, α-CH), 4.20 (q, *J*_HH_ = 7.2 Hz, OCH_2_), 3.47 (ddd, *J*_HH_ = 11.5, 8.7, 3.7 Hz, δ-CH), 3.41 (dt, *J*_HH_ = 11.5, 8.3 Hz, δ-CH), 2.28 and 2.15 (two m, β-CH_2_), 1.93 (s, CH_3_C=O), 1.90 and 1.80 (two m, γ-CH_2_); ^13^C{^1^H} NMR (D_2_O, 176 MHz) δ s-*trans*, 174.7 (s, CO_2_), 173.0 (s, N-C=O), 62.6 (s, OCH_2_), 59.3 (s, α-CH), 48.5 (s, δ-CH_2_), 29.3 (s, β-CH_2_), 24.3 (s, γ-CH_2_), 21.2 (s, CH_3_), 13.2 (s, CH_3_); s-*cis*, 174.2 (s, CO_2_), 173.3 (s, N-C=O), 63.0 (s, OCH_2_), 60.8 (s, α-CH), 46.7 (s, δ-CH_2_), 30.7 (s, β-CH_2_), 22.3 (s, γ-CH_2_), 21.2 (s, CH_3_), 13.2 (s, CH_3_); HRMS (ESI-orbitrap): [M + H]^+^ calcd for C_9_H_16_NO_3_, 186.1125; found, 186.1120; [α]_D_^25^ −81 (*c* 2.0, CHCl_3_).

**2-Fluoroethyl *N*-acetylprolinate (3): **^1^H NMR (D_2_O, 500 MHz) δ s-*trans*, 4.63 (dm, *J*_HF_ = 47 Hz, 2H, CH_2_F), 4.41 (dd, *J*_HH_ = 9.1, 4.6 Hz, 1H, α-CH), 4.37 (ddd, *J*_HH_ = 5.1, 3.0 Hz, *J*_HF_ = 30 Hz, 2H, OCH_2_), 3.62 and 3.59 (two m, 2H, δ-CH_2_), 2.26 and 1.98 (two m, 2H, β-CH_2_), 2.05 (s, 3H, CH_3_C=O), 1.96 (m, 2H, γ-CH_2_); s-*cis*, 4.69 (dd, *J*_HH_ = 8.9, 2.7 Hz, α-CH), 4.65 (m, CH_2_F), 4.43 (m, OCH_2_), 3.49 (ddd, *J*_HH_ = 11.6, 8.6, 3.5 Hz, δ-CH), 3.42 (dt, *J*_HH_ = 11.4, 8.2 Hz, δ-CH), 2.31 and 2.20 (two m, β-CH_2_), 1.95 (s, CH_3_C=O), 1.91 and 1.81 (two m, γ-CH_2_); ^13^C{^1^H} NMR (D_2_O, 126 MHz) δ s-*trans*, 174.2 (s, CO_2_), 173.0 (s, N-C=O), 81.9 (d, *J*_CF_ = 165 Hz, CH_2_F), 64.9 (d, *J*_CF_ = 19 Hz, OCH_2_), 59.1 (s, α-CH), 48.4 (s, δ-CH_2_), 29.2 (s, β-CH_2_), 24.3 (s, γ-CH_2_), 21.2 (s, CH_3_); s-*cis*, 173.8 (s, CO_2_), 173.4 (s, N-C=O), 82.3 (m, CH_2_F), 65.2 (d, *J*_CF_ = 19 Hz, OCH_2_), 60.7 (s, α-CH), 46.6 (s, δ-CH_2_), 30.6 (s, β-CH_2_), 22.3 (s, γ-CH_2_), 21.2 (s, CH_3_); ^19^F NMR (D_2_O, 471 MHz) δ −224.3 (tt, *J*_FH_ = 47, 30 Hz, s-*trans*), −224.6 (tt, *J* = 47, 30 Hz, s-*cis*); HRMS (ESI-orbitrap): [M + H]^+^ calcd for C_9_H_15_FNO_3_, 204.1030; found, 204.1028; [α]_D_^25^ −45 (*c* 2.0, CHCl_3_), er 74:26.

**2,2-Difluoroethyl *N*-acetylprolinate (4): **^1^H NMR (D_2_O, 500 MHz) δ s-*trans*, 6.07 (tt, *J*_HH_ = 3.4 Hz, *J*_HF_ = 54 Hz, 1H, CHF_2_), 4.43 (dd, *J*_HH_ = 9.3, 4.7 Hz, 1H, α-CH), 4.38 (ddt, *J*_HH_ = 3.4, 2.1 Hz, *J*_HF_ = 15 Hz, 2H, OCH_2_), 3.61 and 3.58 (two m, 2H, δ-CH_2_), 2.27 and 1.99 (two m, 2H, β-CH_2_), 2.05 (s, 3H, CH_3_C=O), 1.96 (m, 2H, γ-CH_2_); s-*cis*, 6.11 (tt, *J*_HH_ = 3.2 Hz, *J*_HF_ = 54 Hz, CHF_2_), 4.74 (dd, *J*_HH_ = 8.8, 2.5 Hz, α-CH), 4.44 (m, OCH_2_), 3.50 (ddd, *J*_HH_ = 11.6, 8.6, 3.4 Hz, δ-CH), 3.43 (dt, *J*_HH_ = 11.5, 8.3 Hz, δ-CH), 2.32 and 2.20 (two m, β-CH_2_), 1.95 (s, CH_3_C=O), 1.92 and 1.80 (two m, γ-CH_2_); ^13^C{^1^H} NMR (D_2_O, 126 MHz) δ s-*trans*, 173.4 (s, CO_2_), 173.0 (s, N-C=O), 113.0 (t, *J*_CF_ = 240 Hz, CHF_2_), 63.0 (t, *J*_CF_ = 27 Hz, OCH_2_), 58.9 (s, α-CH), 48.4 (s, δ-CH_2_), 29.2 (s, β-CH_2_), 24.3 (s, γ-CH_2_), 21.1 (s, CH_3_); s-*cis*, 173.4 (s, N-C=O), 173.0 (s, CO_2_), 113.0 (m, CHF_2_), 63.3 (d, *J*_CF_ = 27 Hz, OCH_2_), 60.6 (s, α-CH), 46.6 (s, δ-CH_2_), 30.6 (s, β-CH_2_), 22.3 (s, γ-CH_2_), 21.2 (s, CH_3_); ^19^F NMR (D_2_O, 471 MHz) δ −126.92 (dtd, *J*_FF_ = 292 Hz, *J*_FH_ = 53, 15 Hz, 1F, s-*trans*), −126.93 (dtd, *J*_FF_ = 292 Hz, *J*_FH_ = 53, 15 Hz, 1F, s-*trans*), −127.20 (dt, *J*_FH_ = 54, 15 Hz, s-*cis*); HRMS (ESI-orbitrap): [M + H]^+^ calcd for C_9_H_14_F_2_NO_3_, 222.0936; found, 222.0933; [α]_D_^25^ −76 (*c* 2.0, CHCl_3_).

**2,2,2-Trifluoroethyl *N*-acetylprolinate (5): **^1^H NMR (D_2_O, 700 MHz) δ s-*trans*, 4.78 (q, *J*_HF_ = 8.7 Hz, 2H, ΟCH_2_), 4.59 (dd, *J*_HH_ = 9.2, 4.7 Hz, 1H, α-CH), 3.63 and 3.59 (two m, 2H, δ-CH_2_), 2.29 and 2.01 (two m, 2H, β-CH_2_), 2.06 (s, 3H, CH_3_C=O), 1.98 (m, 2H, γ-CH_2_); s-*cis*, 4.78 (dd, *J*_HH_ = 8.8, 2.4 Hz, α-CH), 4.71 (m, OCH_2_), 3.50 (ddd, *J*_HH_ = 11.6, 8.8, 3.3 Hz, δ-CH), 3.43 (dt, *J*_HH_ = 11.5, 8.1 Hz, δ-CH), 2.33 and 2.22 (two m, β-CH_2_), 1.97 (s, CH_3_C=O), 1.94 and 1.82 (two m, γ-CH_2_); ^13^C{^1^H} NMR (D_2_O, 176 MHz) δ s-*trans*, 173.4 (s, N-C=O), 172.6 (s, CO_2_), 122.9 (q, *J*_CF_ = 277 Hz, CF_3_), 60.9 (q, *J*_CF_ = 36 Hz, OCH_2_), 58.8 (s, α-CH), 48.3 (s, δ-CH_2_), 29.1 (s, β-CH_2_), 24.3 (s, γ-CH_2_), 21.1 (s, CH_3_); s-*cis*, 173.4 (s, N-C=O), 172.2 (s, CO_2_), 122.9 (q, *J*_CF_ = 276 Hz, CF_3_), 61.3 (d, *J*_CF_ = 36 Hz, OCH_2_), 60.5 (s, α-CH), 46.6 (s, δ-CH_2_), 30.6 (s, β-CH_2_), 22.3 (s, γ-CH_2_), 21.2 (s, CH_3_); ^19^F NMR (D_2_O, 471 MHz) δ −73.6 (t, *J*_FH_ = 9 Hz, s-*trans*), −73.7 (t, *J*_FH_ = 8 Hz, s-*cis*); HRMS (ESI-orbitrap): [M + H]^+^ calcd for C_9_H_13_F_3_NO_3_, 240.0842; found, 240.0837; [α]_D_^25^ −13 (*c* 2.0, CHCl_3_), er 59:41.

#### Synthesis of the peptides

Ac(Pro)_6_OH (**8a**) and Ac(^ε^BocLys)*_n_*OH (*n* = 3, 6) peptides were synthesized on pre-loaded 2-chlorotrityl resin conventionally. The esterification with 2,2-difluorodiazoethane was performed as described in [46]. Alternatively, the procedure was performed as follows: *tert*-Butyl nitrite (≈ 6 μL) and 2,2-difluoroethylamine (≈ 11 μL) were mixed in chloroform (100 μL), and this mixture was shaken at room temperature for 10 min. It was then added to the peptide (25 μmol; in this example 18.4 mg of Ac(^ε^BocLys)_3_OH) soaked in chloroform (100 μL). The resulting mixture was shaken at room temperature for approximately 10 hours. Volatiles were removed with nitrogen gas, and the residue was freeze-dried from an acetonitrile/water mixture to afford the target peptides. The Boc-protected peptides were purified on a silica gel column using a dichloromethane/methanol 9:1 → 1:0 gradient elution. The Boc deprotection was performed as follows. The peptide (3–4 mg) was mixed with 4 M hydrogen chloride in dioxane (100 μL), and the turbid mixture was shaken at room temperature for 20 min. Water (100 μL) was added, resulting a clear solution, which was shaken for an additional 20 min. The mixture was then freeze-dried from water to afford target peptides **9a**,**b** or **10a**,**b**.

The esterification of **8a** with methanol was performed as follows. Ac(Pro)_6_OH (**8a**, 5.6 mg) was dissolved in methanol (0.5 mL), and trimethylsilyl chloride (0.08 mL) was added. The mixture was shaken at room temperature for 24 hours. Volatiles were removed with nitrogen gas, and the residue was freeze-dried from water. No HPLC purification has been applied in order to avoid ion-pairing agent contaminations and hydrolysis of the esters. Additionally, full conversion was observed in the synthesis of peptides **8b** and **8c**.

Oligolysine peptides were esterified as follows. A peptide Ac(^ε^BocLys)*_n_*OH (*n* = 3, 6; 3 mg) was dissolved in methanol (1 mL), and trimethylsilyl chloride (0.08 mL) was added. The mixture was shaken at ambient temperature for 20 hours. Solvent was removed with nitrogen gas, and the residues were freeze-dried from deuterium oxide (0.2 mL) to afford target peptides **9c**, **10c**.

#### Characterization of the peptides

Mass spectra were recorded using high-resolution electrospray-orbitrap mass spectrometry ionization (HRMS) for all peptides except hexalysine peptides **10a**–**c**, which produced no distinguishable molecular ions. For cationic peptides **9a**–**c** and **10a**–**c**, additional MALDI–TOF analysis confirmed the molecular assignment.

Diffusion coefficients were determined in a ^1^H stimulated echo experiment with bipolar gradients and a spoil pulse. The settings were as follows: 298 K, deuterium oxide, 700 MHz, Δ = 30 ms, δ/2 = 2 ms, spoil pulse 0.5 ms, 128 array experiments with linear gradient increase. The reported error value is the distance between the midpoint and the top of the log*D* projection peak. Experimental log*D* values were compared to the calculated ones (Equation 5).

Circular dichroism spectra were recorded in a 1 mm quartz cell at 298 K in methanol (HPLC grade) or aqueous potassium phosphate buffer (50 mM, pH 7.01 at 297 K). The peptide concentration was 100 μM as determined gravimetrically. The mean residue absorption difference Δε was calculated assuming 6 residues for peptides **8a**–**c**, **10a**–**c** and 3 residues for peptides **9a**–**c**. Hydrolysis was measured in deuterium oxide solution of a 150 mM potassium phosphate buffer. The buffer pH 7.01 was adjusted in water at 298 K. It was then lyophilized, then dissolved in deuterium oxide. The peptides were dissolved in the buffer, and the resulting samples were kept at 298 ± 2 K, while the ^19^F NMR measurements were conducted at several time points. The starting concentrations of the analytes were 8 mM for **8b**, 5 mM for **9b** and 2.5 mM for **10b**. The half-life was calculated using the first (zero) order kinetic model.

Ac(Pro)_6_OH, **8a**. HRMS: calcd for [M + H]^+^ 643.3450, for [M + Na]^+^ 665.3269; found, 643.3443 and 665.3257; log*D*, calcd for [M] −9.462; found, −9.468 ± 0.026.

Ac(Pro)_6_OCH_2_CHF_2_, **8b**. HRMS: calcd for [M + H]^+^ 707.3574, for [M + Na]^+^ 729.3394; found, 707.3566 and 729.3377; log*D*, calcd for [M] −9.475; found, −9.477 ± 0.011; τ_½_ = 152 ± 14 (139 ± 20) days (buffered deuterium oxide, pH 7.0).

Ac(Pro)_6_OCH_3_, **8c**. HRMS: calcd for [M + H]^+^ 657.3606, for [M + Na]^+^ 679.3426; found, 657.3599 and 679.3416; log*D*, calcd for [M] −9.465; found, −9.467 ± 0.027.

Ac(Lys)_3_OH∙3HCl, **9a**. HRMS: calcd for [M + 3H]^3+^ 149.1093; found, 149.1096; MALDI–TOF: calcd for [M + H]^+^ 445.3; found, 445.2; log*D*, calcd for [M + 3H^+^] −9.410; found, −9.442 ± 0.025.

Ac(Lys)_6_OH∙6HCl, **10a**. MALDI–TOF: calcd for [M + H]^+^ 829.6; found, 829.5; log*D*, calcd for [M + 6H^+^] −9.499; found, −9.607 ± 0.038.

Ac(Lys)_3_OCH_2_CHF_2_∙3HCl, **9b**. HRMS: calcd for [M + 3H]^3+^ 170.4468; found, 170.4474; MALDI–TOF: calcd for [M + H]^+^ 509.3; found, 509.2; log*D*, calcd for [M + 3H^+^] −9.429; found, −9.439 ± 0.021; τ_½_ = 5.0 ± 0.3 (4.5 ± 0.5) days (buffered deuterium oxide, pH 7.0).

Ac(Lys)_6_OCH_2_CHF_2_∙6HCl, **10b**. MALDI–TOF: calcd for [M + H]^+^ 893.6; found, 893.6; log*D*, calcd for [M + 6H^+^] −9.510; found, −9.583 ± 0.025; τ_½_ = 4.0 ± 0.3 (3.7 ± 0.3) days (buffered deuterium oxide, pH 7.0).

Ac(Lys)_3_OCH_3_·3HCl, **9c**. HRMS: calcd for [M + 3H]^3+^ 153.7812; found , 153.7812. MALDI–TOF: calcd for [M + H]^+^ 459.3; found, 459.6; log*D*, calcd for [M + 3H^+^] −9.415; found, −9.425 ± 0.010.

Ac(Lys)_6_OCH_3_·6HCl, **10c**. MALDI–TOF: calcd for [M + H]^+^ 843.6; found, 844.3; log*D*, calcd for [M + 6H^+^] −9.502; found, −9.559 ± 0.017.

## Supporting Information

File 1 Amide equilibrium constants (Table S1) and copies of the NMR and CD spectra.
