# Supplementary material for: Hydrolysis, polarity, and conformational impact of C-terminal partially fluorinated ethyl esters in peptide models
Source: Beilstein J Org Chem. 2017 Nov 16;13:2442–57. doi: 10.3762/bjoc.13.241 (PMC5704756; doi:10.3762/bjoc.13.241)

Supporting Information

for

Hydrolysis, polarity, and conformational impact of C-terminal partially fluorinated ethyl esters in peptide models

Vladimir Kubyshkin* and Nediljko Budisa*

Address: Biocatalysis group, Institute of Chemistry, Technical University of Berlin, Müller-Breslau-Strasse 10, Berlin 10623, Germany

Email: Vladimir Kubyshkin - kubyshkin@win.tu-berlin.de; Nediljko Budisa - nediljko.budisa@tu-berlin.de

*Corresponding author

Amide equilibrium constants (Table S1) and copies of the NMR and CD spectra

Table of contents

Table S1 S2

Copies of the NMR spectra for compounds **1–5** S3–S25

^{1}H NMR spectrum of compound **7** S26

NMR spectra of **3–5** with the europium shift reagent S27–S29

NMR spectra of the peptides S30–S36

Circular dichroism spectra for the peptides S37–S39

Hydrolysis of the peptides S40–S44

Remark: We use the notation ✔ (checkmark)-shape for description of the log*P* tendencies. On our opinion, this should be distinguished from more simple notation 'V-shape' due to the asymmetry of both the dip position and the edge highs.

Table S1: Amide equilibrium constants for compounds **1–5** as determined by ^{1}H and ^{19}F NMR at 298 K in different solvents.

compound	$K_{trans/cis}$					
	D_2O	CD_3CN	CD_3OD	CD_2Cl_2	$CDCl_3$	C_6D_6
	ε 80.1	ε 32.7	ε 32.7	ε 8.93	ε 4.81	ε 2.27
**1**	4.95±0.05	4.08±0.04	3.78±0.03	4.18±0.03	3.84±0.04	5.10±0.10
**2**	4.60±0.08	3.58±0.04	3.49±0.04	3.52±0.03	3.20±0.04	4.67±0.03
**3**	4.74±0.04	4.12±0.17	3.61±0.03	4.77±0.10	4.44±0.20	6.80±0.04
**4**	4.95±0.05	4.85±0.07	4.12±0.03	6.57±0.14	6.29±0.22	9.06±0.26
**5**	5.48±0.14	5.65±0.05	4.49±0.05	7.07±0.11	6.65±0.18	10.04±0.15

Copies of the NMR spectra for compounds 1–5

^{1}H NMR spectrum of **1** in deuterium oxide at 700 MHz

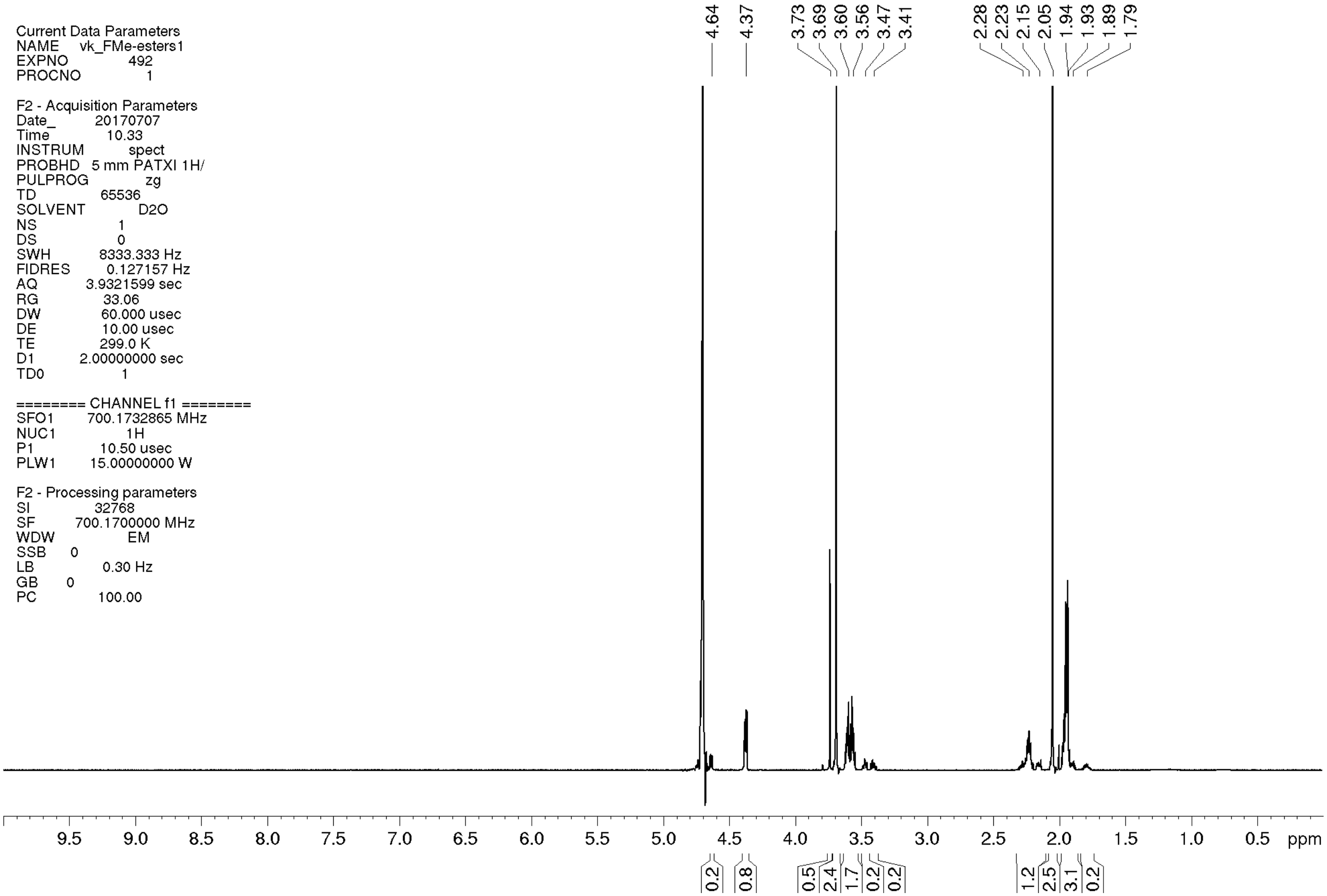

$^{13}C\{^{1}H\}$ NMR spectrum of **1** in deuterium oxide at 126 MHz

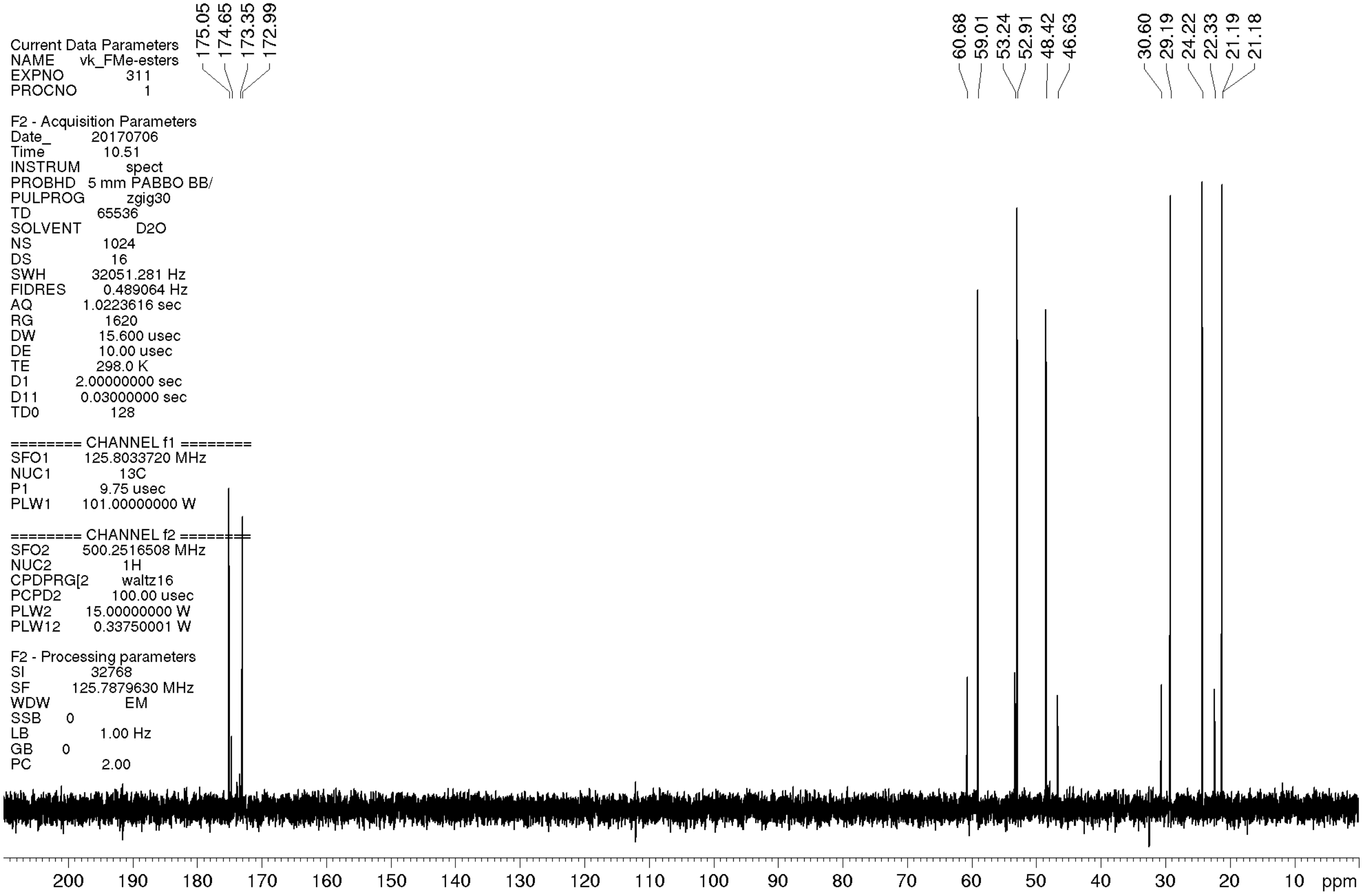

^{1}H NMR spectrum of **1** in benzene-d_6 at 700 MHz

Current Data Parameters
NAME vk_FMe-esters1
EXPNO 480
PROCNO 1

F2 - Acquisition Parameters
Date_ 20170706
Time 21.59
INSTRUM spect
PROBHD 5 mm PATXI 1H/
PULPROG zg
TD 65536
SOLVENT C6D6
NS 1
DS 0
SWH 8333.333 Hz
FIDRES 0.127157 Hz
AQ 3.9321599 sec
RG 51.58
DW 60.000 usec
DE 10.00 usec
TE 299.0 K
D1 2.00000000 sec
TD0 1

======== CHANNEL f1 ========
SFO1 700.1732865 MHz
NUC1 1H
P1 10.40 usec
PLW1 15.00000000 W

F2 - Processing parameters
SI 32768
SF 700.1700000 MHz
WDW EM
SSB 0
LB 0.30 Hz
GB 0
PC 100.00

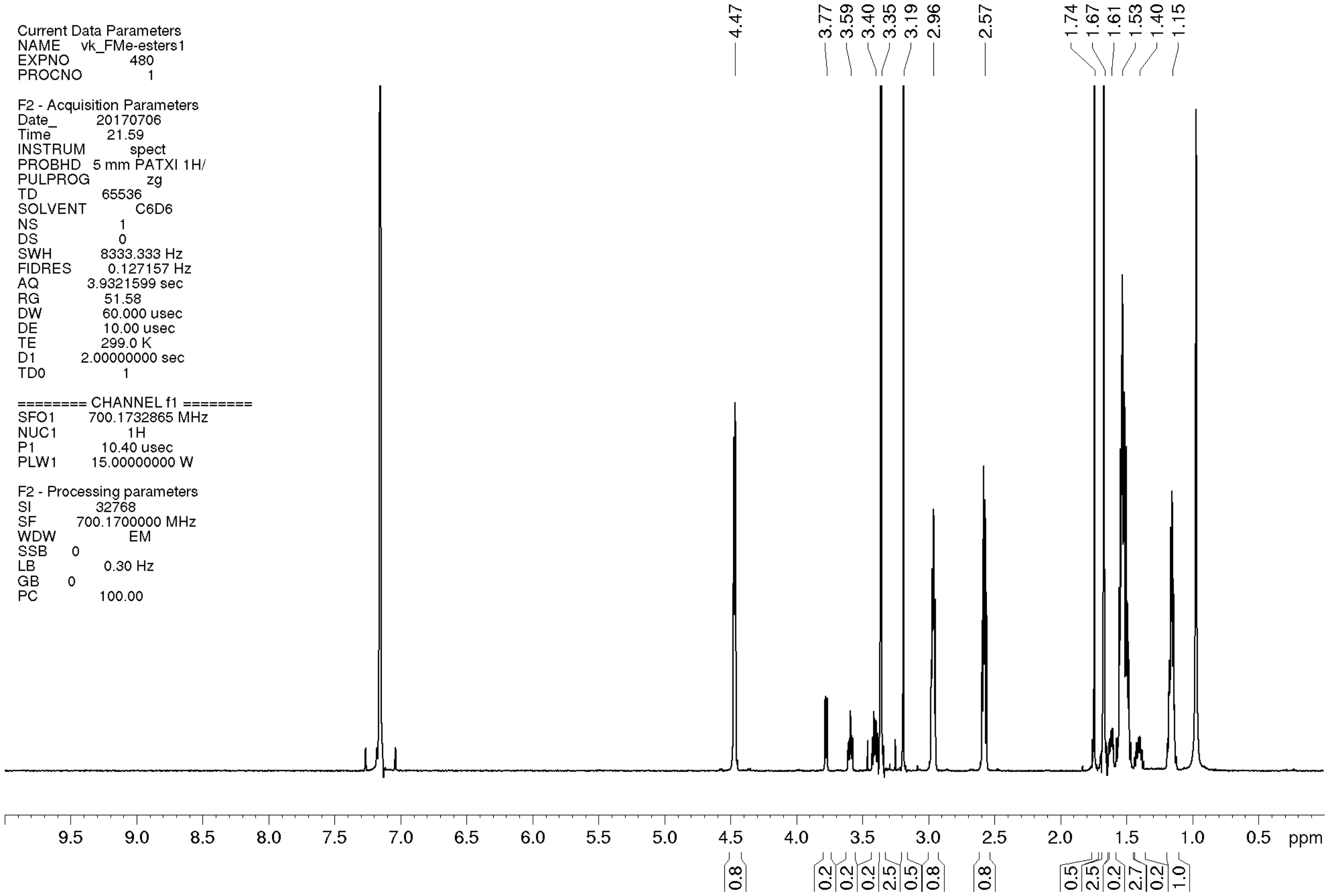

$^{13}C\{^{1}H\}$ NMR spectrum of **1** in benzene-d_6 at 176 MHz

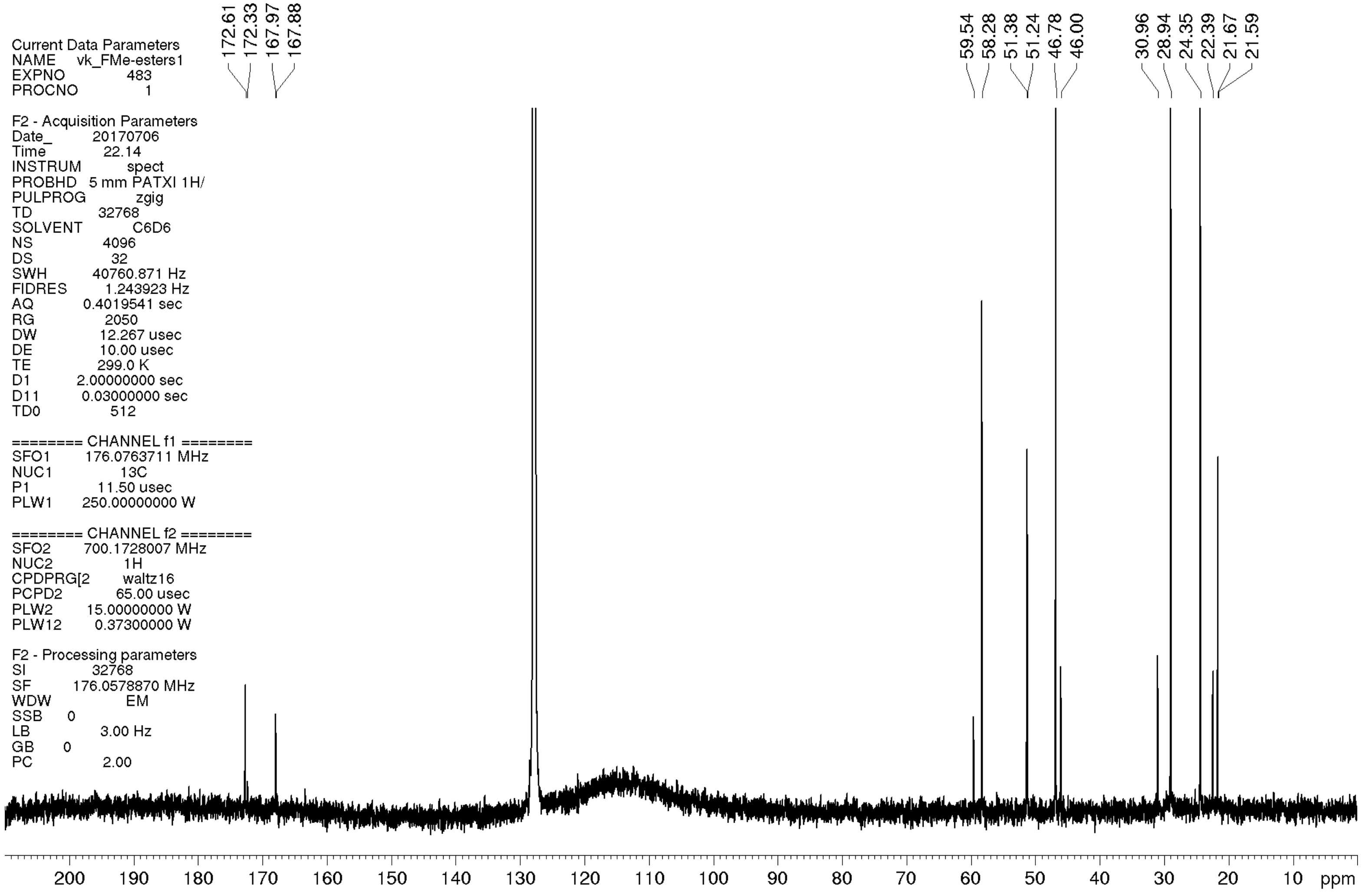

^{1}H NMR spectrum of **2** in deuterium oxide at 700 MHz

Current Data Parameters
NAME vk_FMe-esters1
EXPNO 70
PROCNO 1

F2 - Acquisition Parameters
Date_ 20161109
Time 17.02
INSTRUM spect
PROBHD 5 mm PATXI 1H/
PULPROG zg
TD 65536
SOLVENT D2O
NS 1
DS 0
SWH 8333.333 Hz
FIDRES 0.127157 Hz
AQ 3.9321599 sec
RG 41.2
DW 60.000 usec
DE 10.00 usec
TE 299.0 K
D1 2.00000000 sec
TD0 1

======== CHANNEL f1 ========
SFO1 700.1732865 MHz
NUC1 1H
P1 10.25 usec
PLW1 15.00000000 W

F2 - Processing parameters
SI 32768
SF 700.1700000 MHz
WDW EM
SSB 0
LB 0.30 Hz
GB 0
PC 100.00

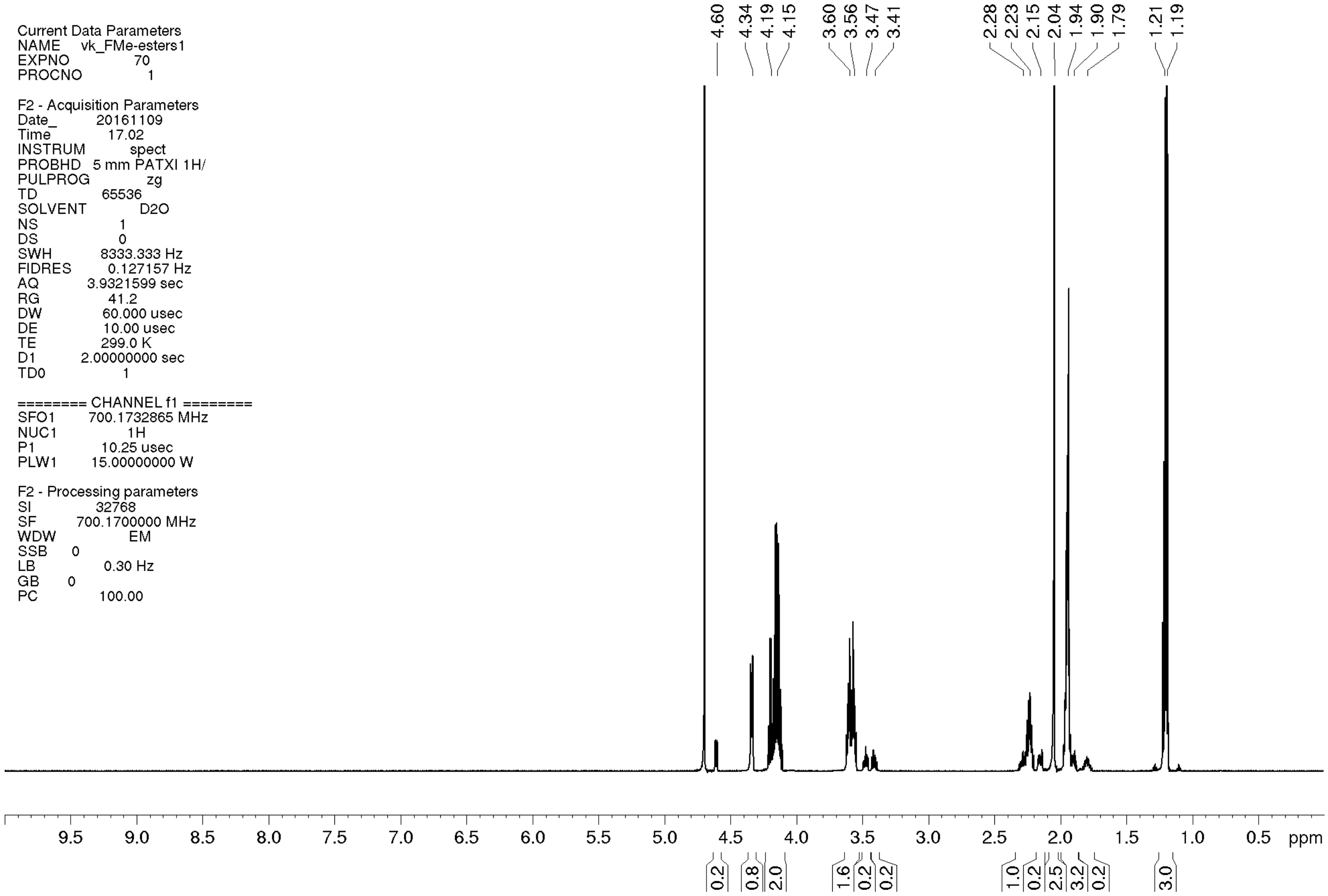

$^{13}C\{^{1}H\}$ NMR spectrum of **2** in deuterium oxide at 176 MHz

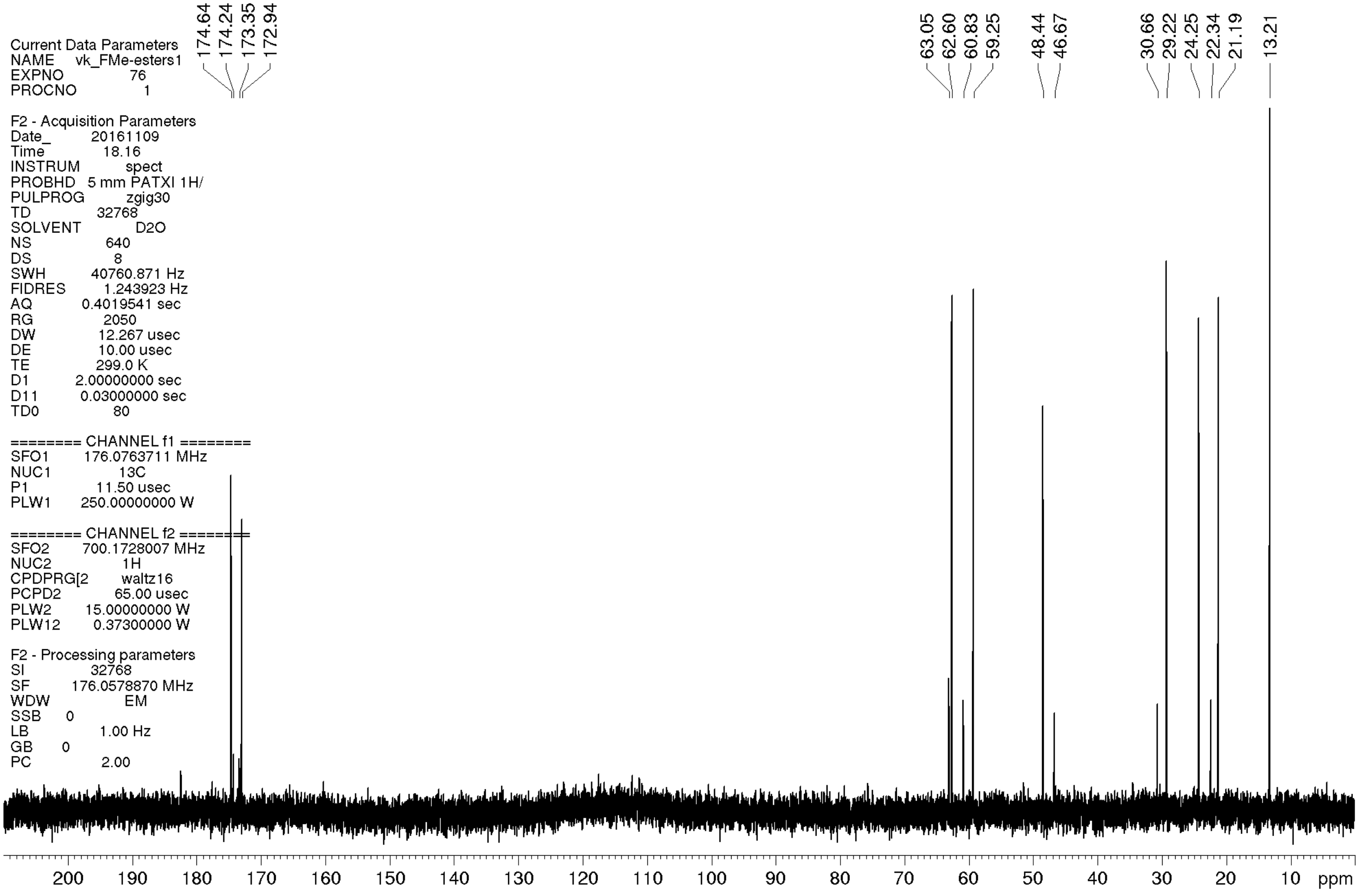

^{1}H NMR spectrum of **2** in benzene-d_6 at 700 MHz

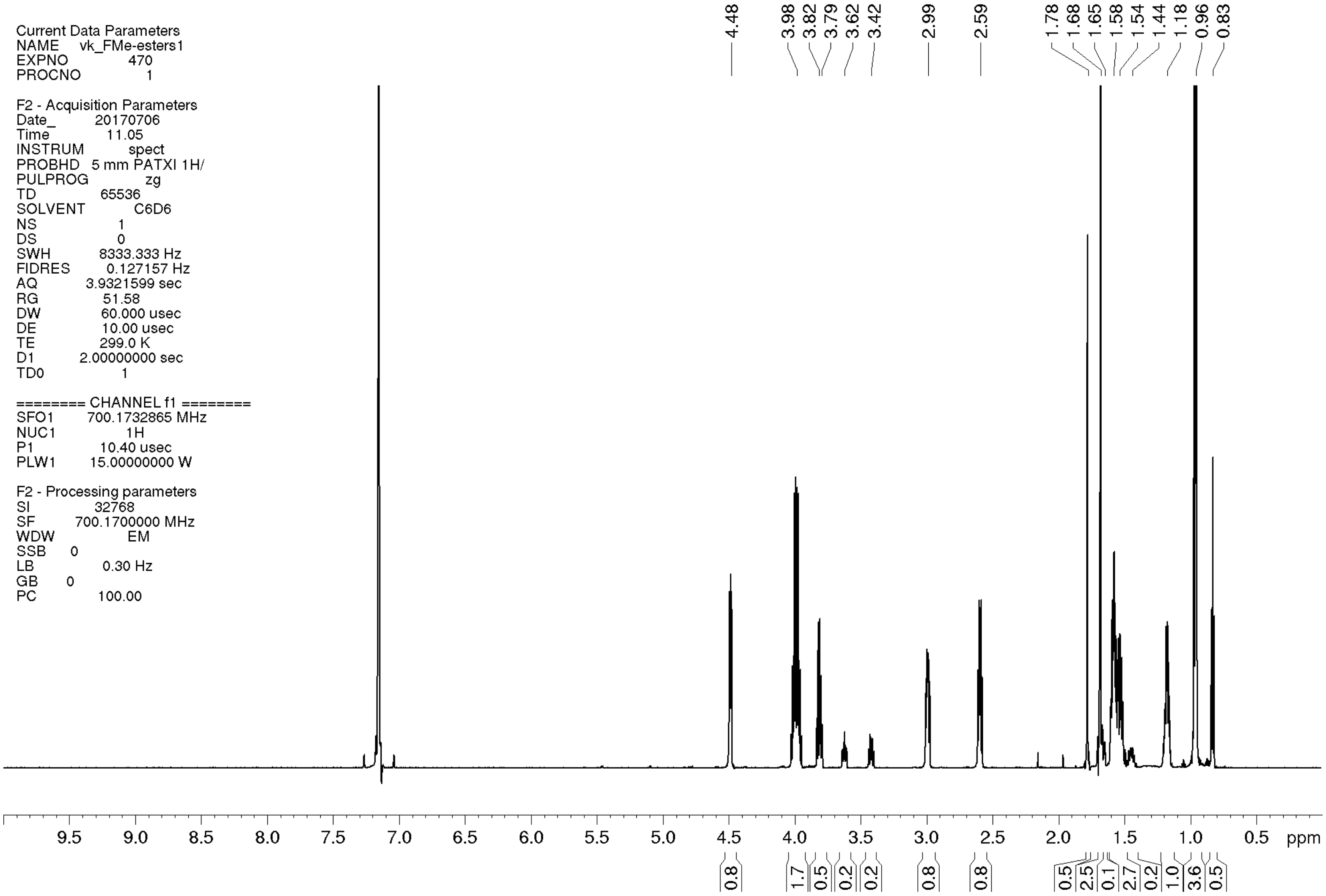

$^{13}C\{^1H\}$ NMR spectrum of **2** in benzene-d_6 at 176 MHz

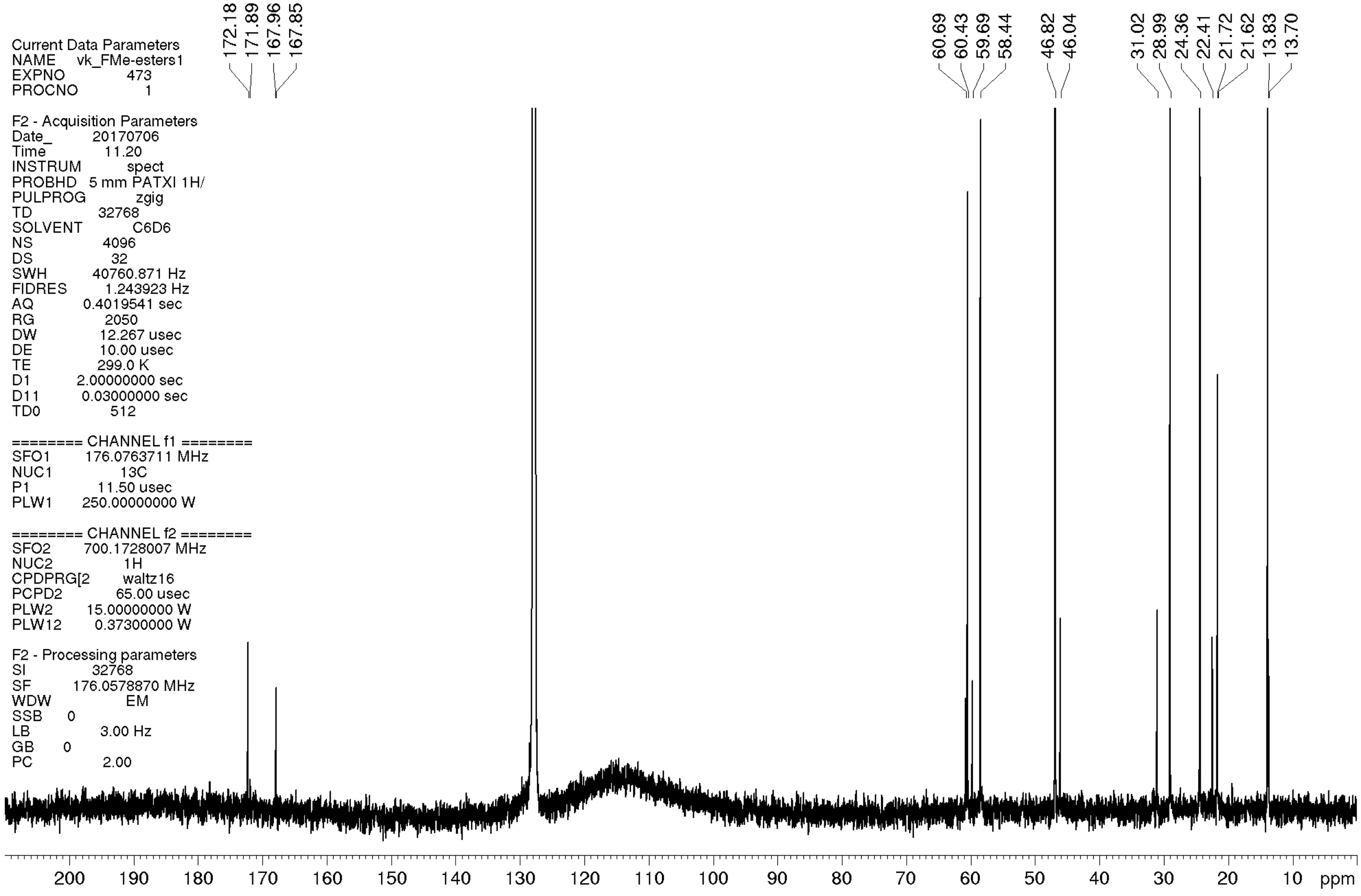

^{1}H and ^{1}H{^{19}F} NMR spectra of **3** in deuterium oxide at 500 MHz

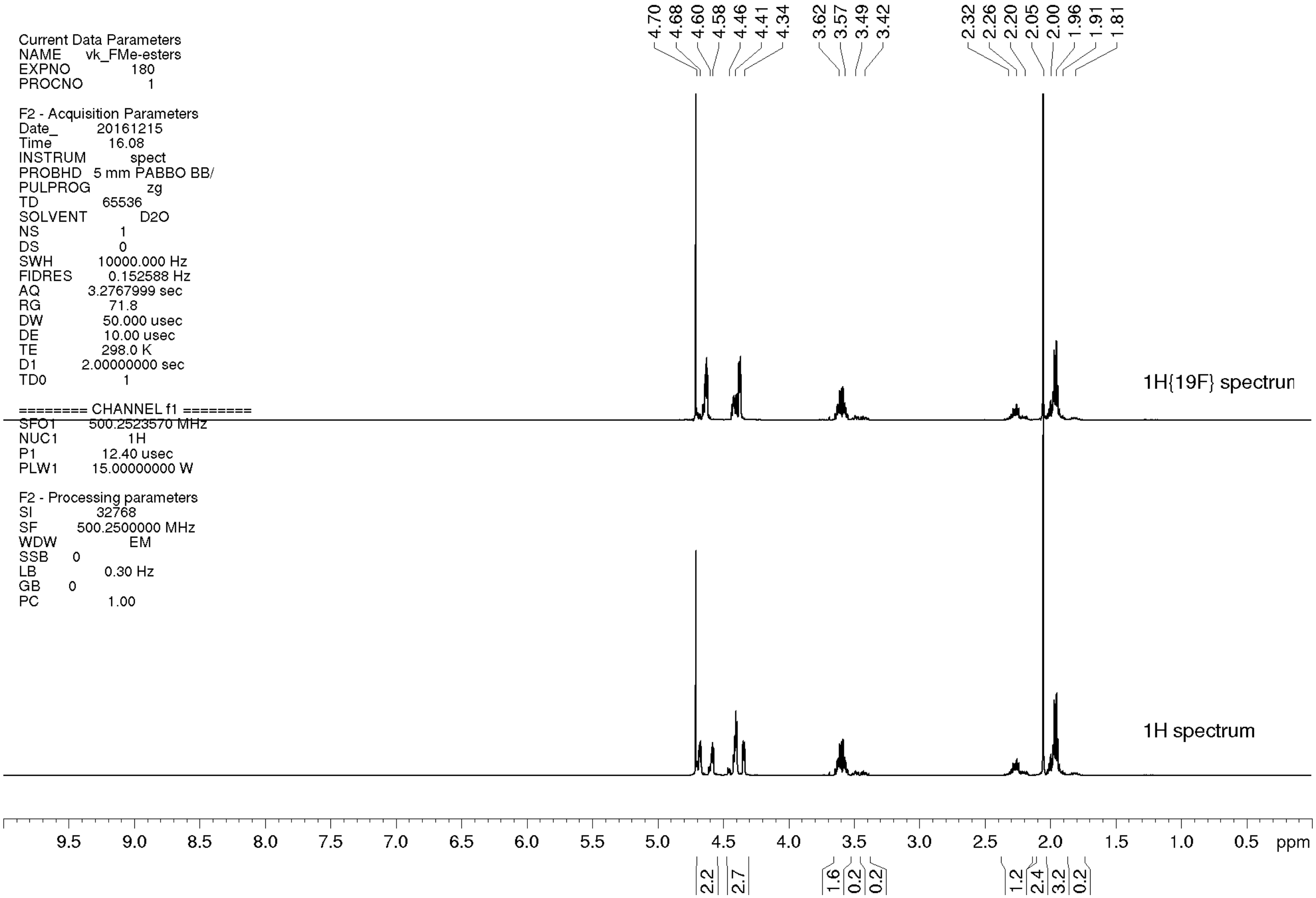

$^{13}C\{^1H\}$ NMR spectrum of **3** in deuterium oxide at 126 MHz

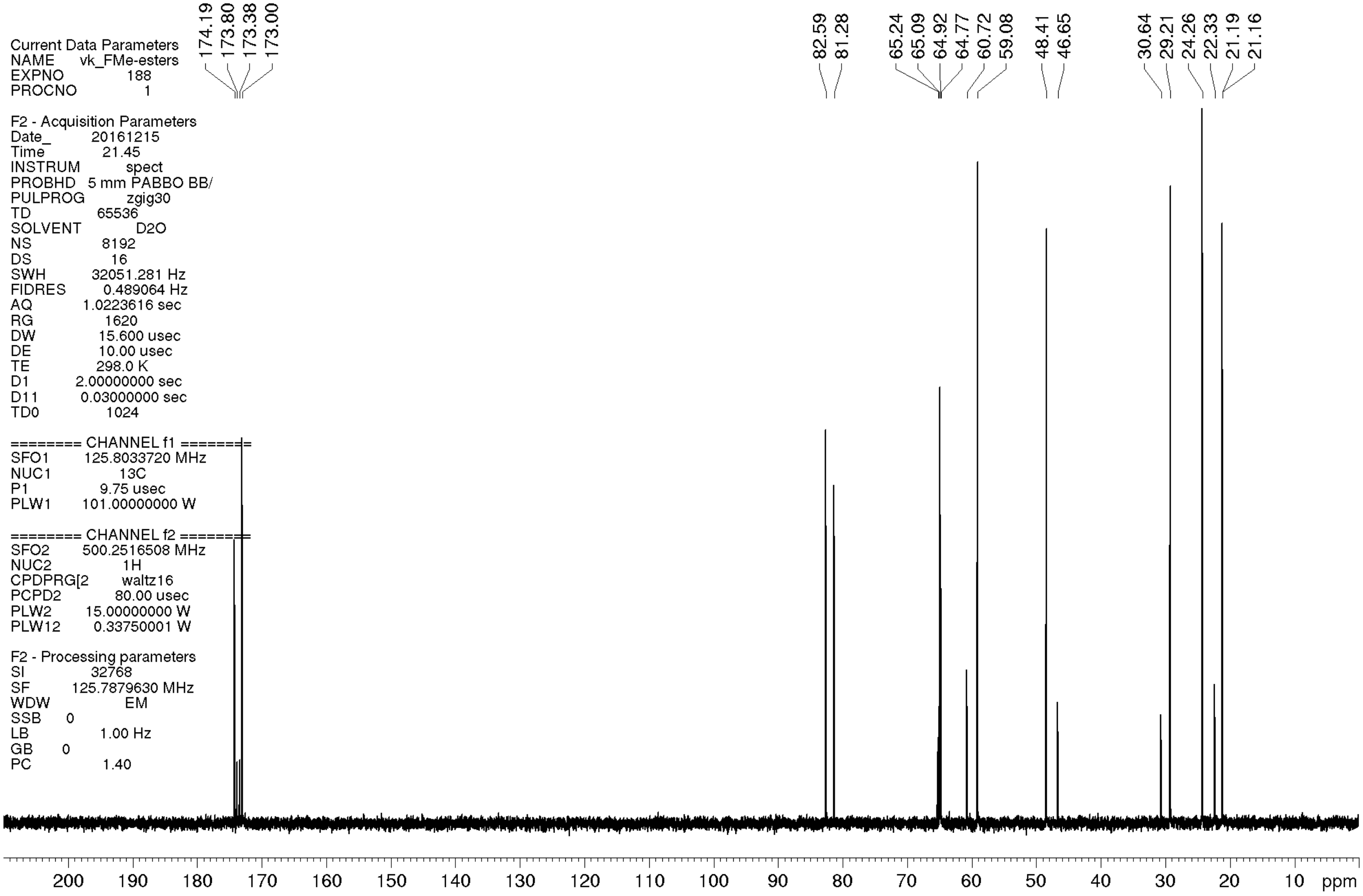

^{19}F and $^{19}F\{^1H\}$ NMR spectra of **3** in deuterium oxide at 471 MHz

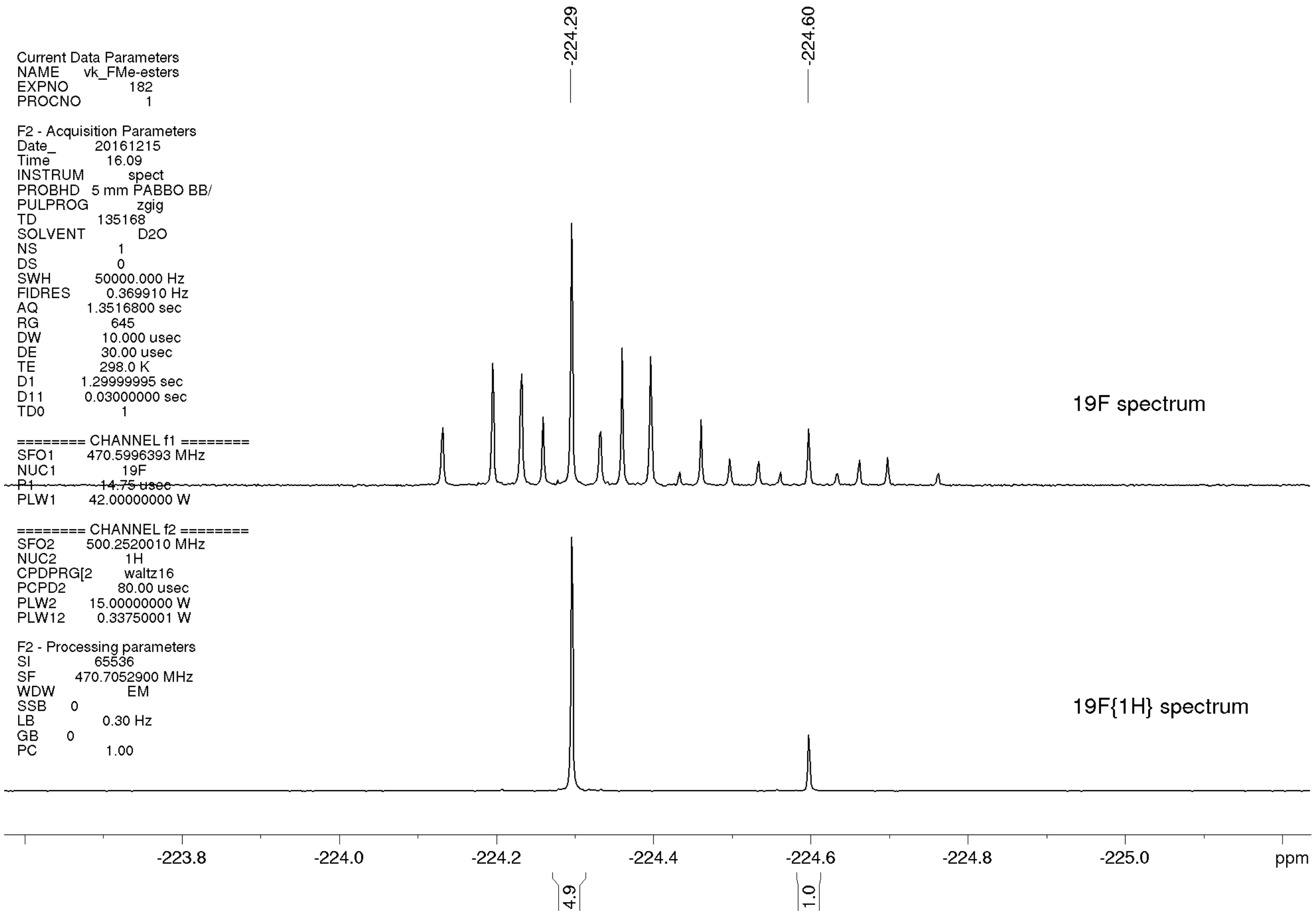

^{1}H NMR spectrum of **3** in benzene-d_6 at 700 MHz

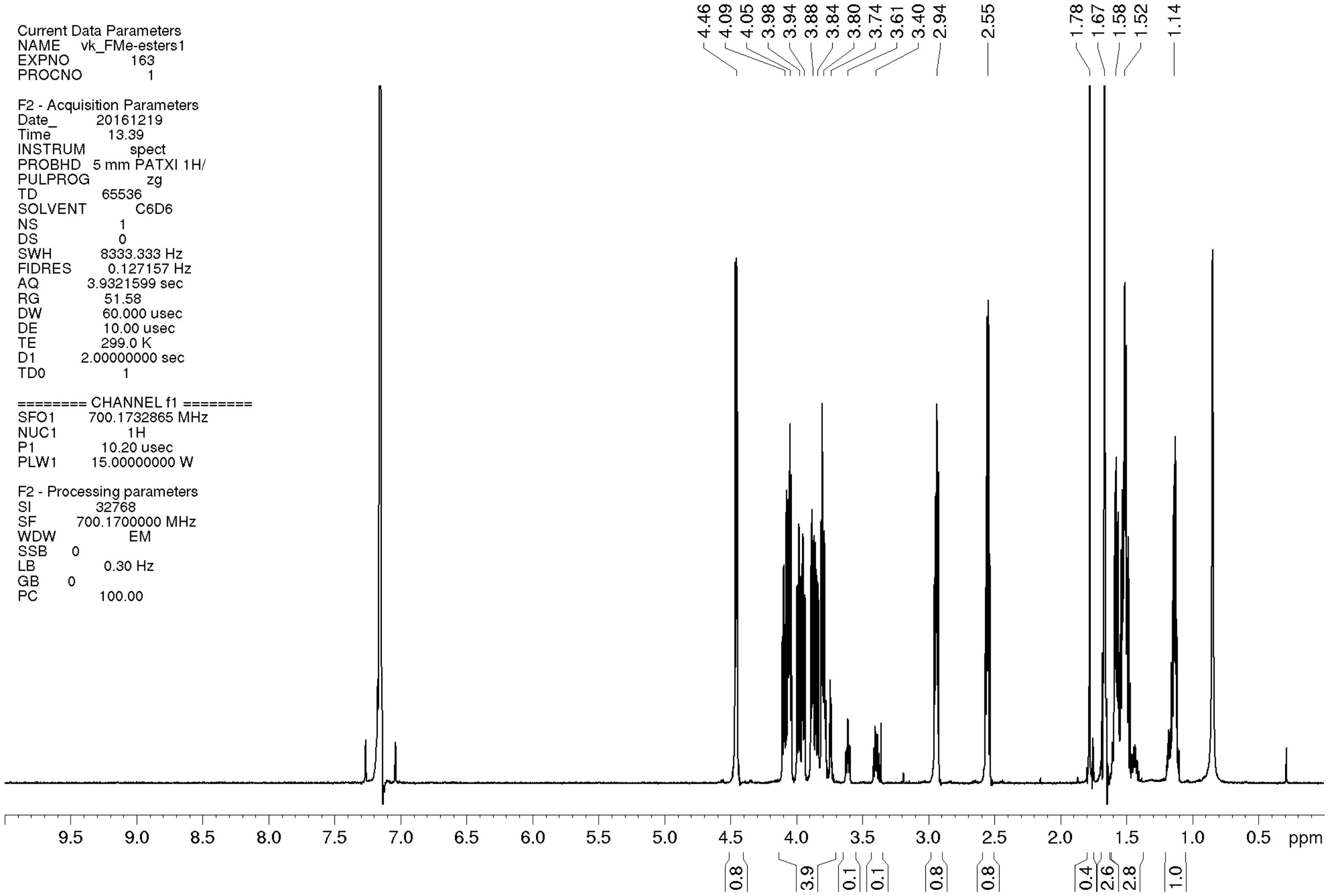

$^{13}C\{^{1}H\}$ NMR spectrum of **3** in benzene-d_6 at 176 MHz

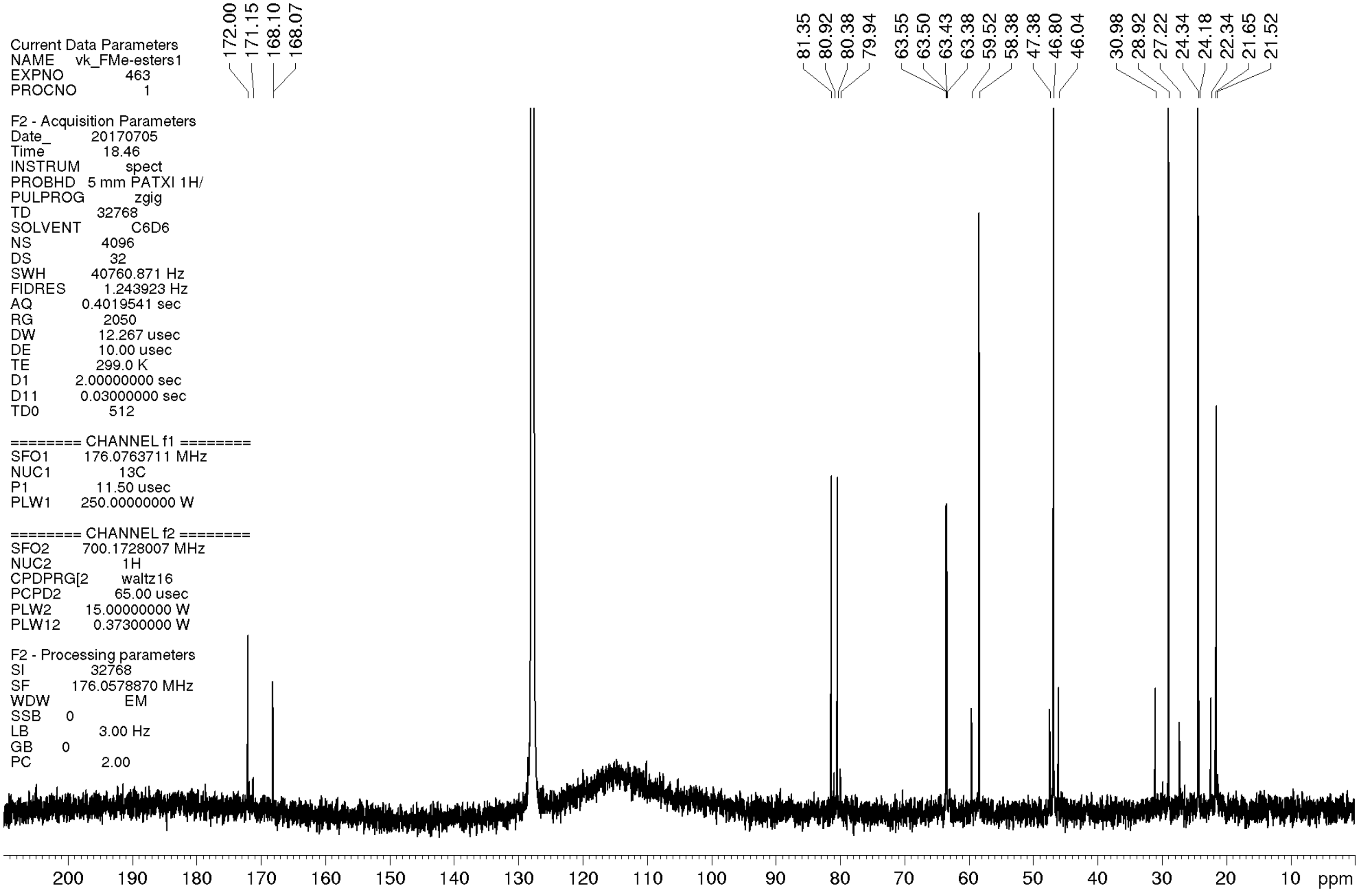

^{1}H and $^{1}H\{^{19}F\}$ NMR spectra of **4** in deuterium oxide at 500 MHz

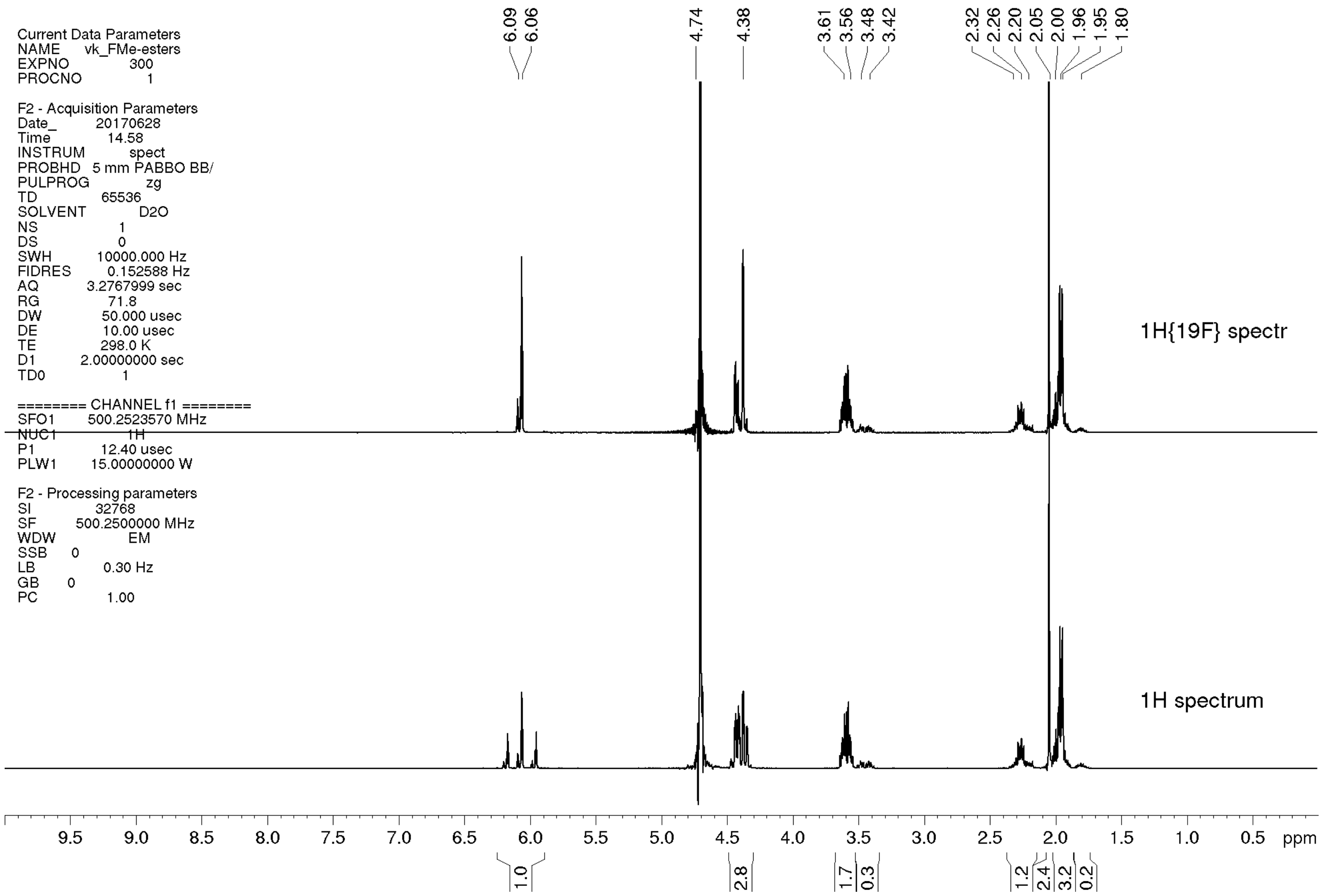

^{19}F and $^{19}F\{^1H\}$ NMR spectra of **4** in deuterium oxide at 471 MHz

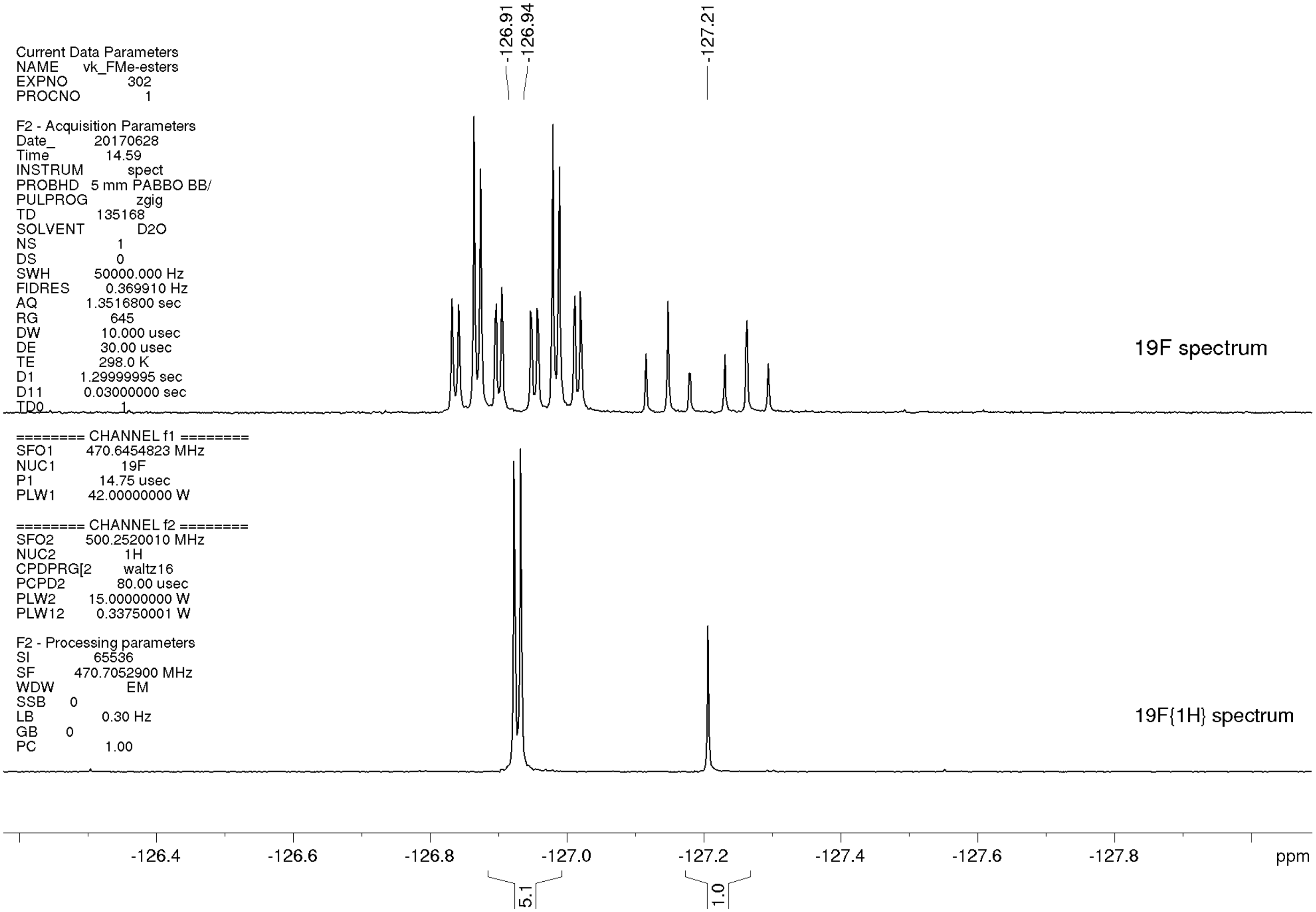

^{1}H NMR spectrum of **4** in benzene-d_6 at 700 MHz

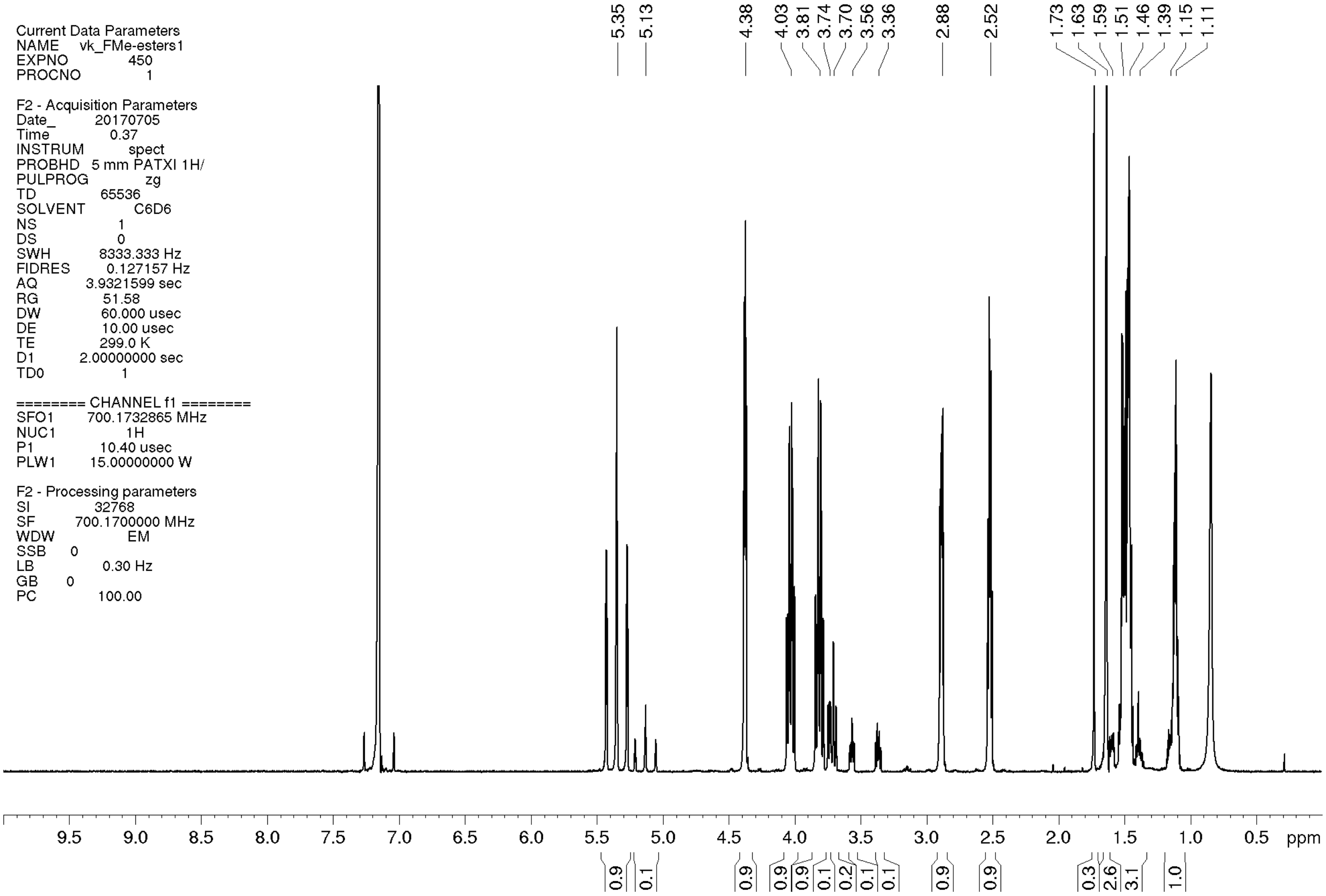

$^{13}C\{^{1}H\}$ NMR spectrum of **4** in benzene-d_6 at 176 MHz

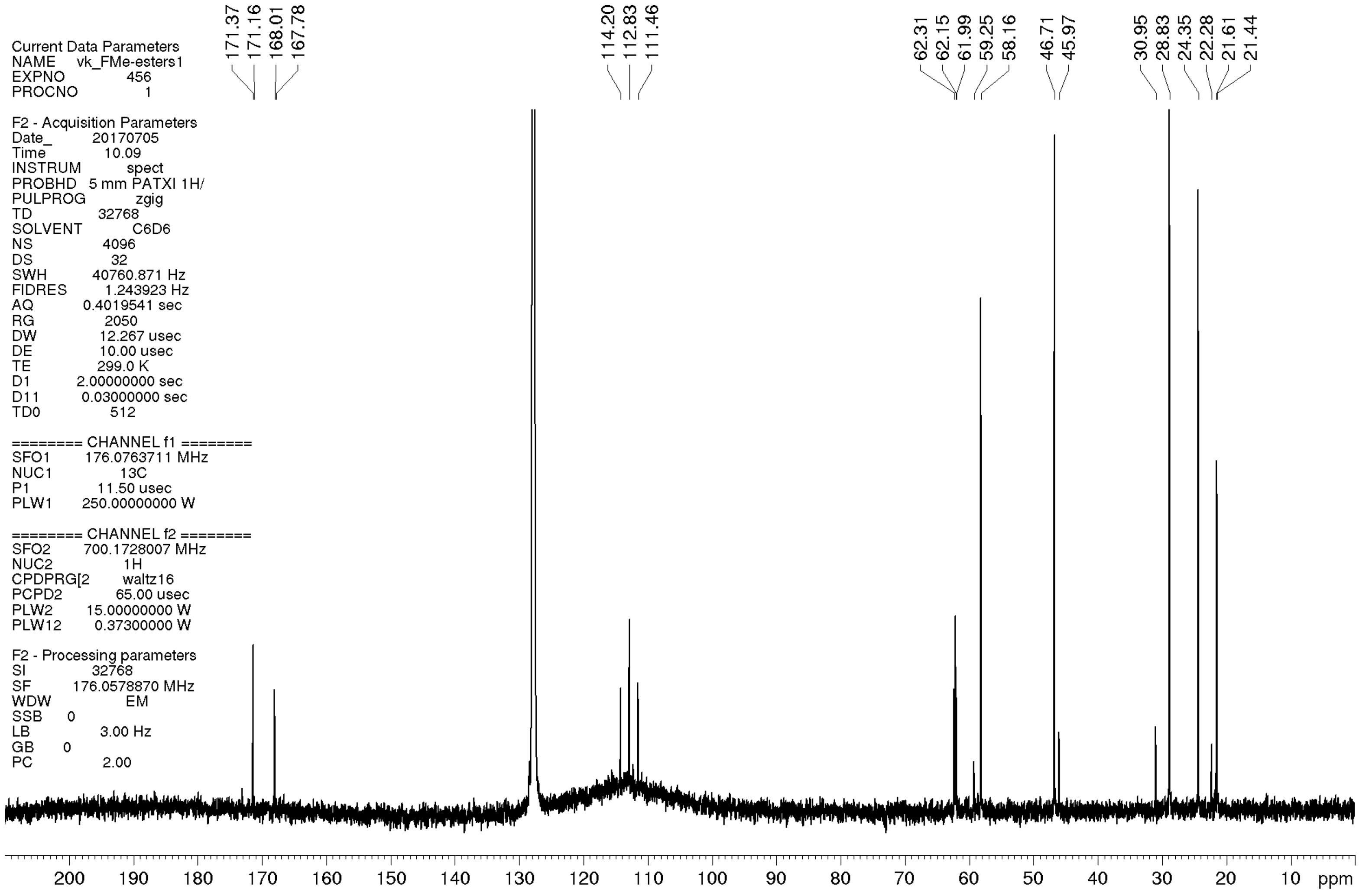

^{1}H NMR spectrum of **5** in deuterium oxide at 700 MHz

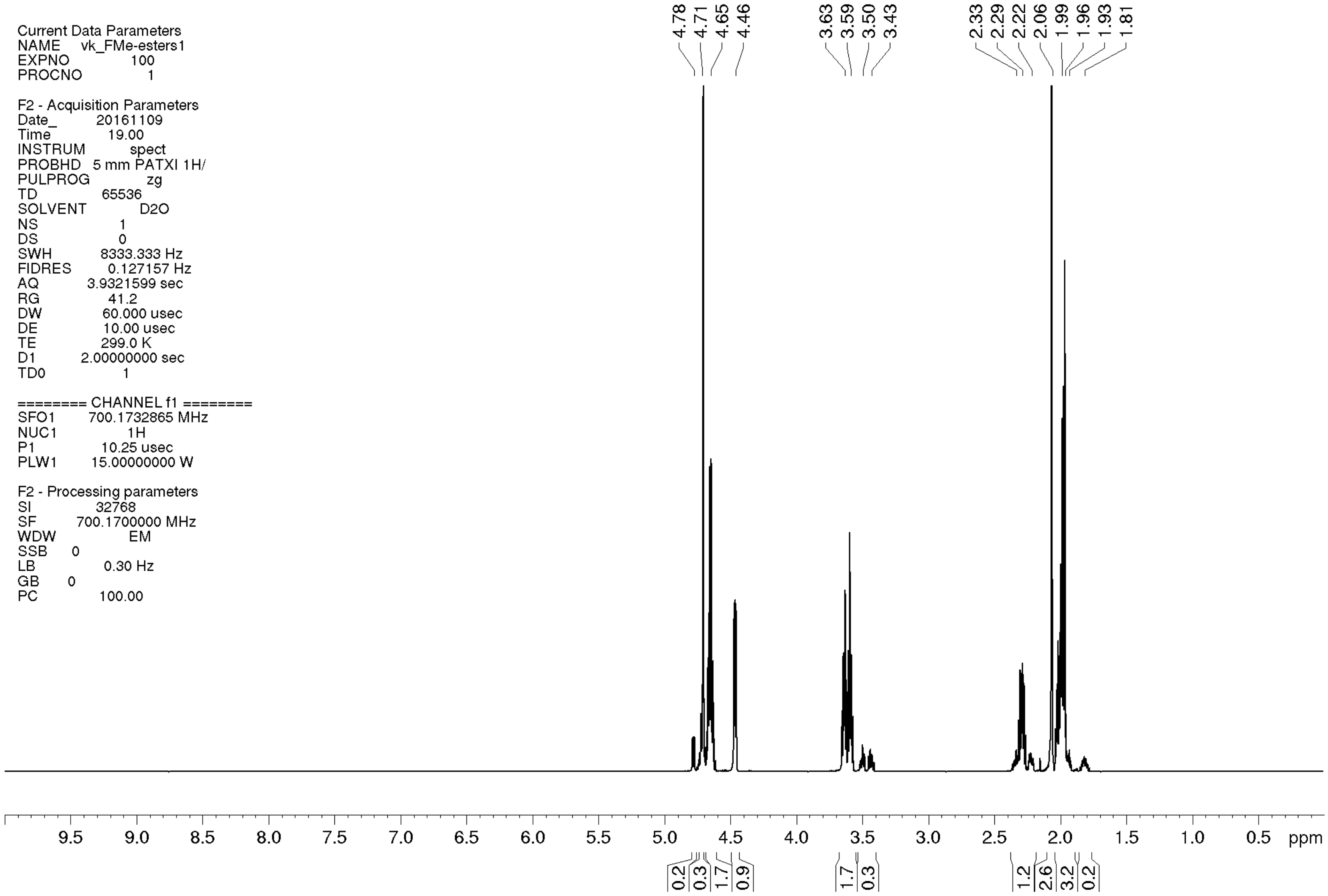

$^{13}C\{^1H\}$ NMR spectrum of **5** in deuterium oxide at 176 MHz

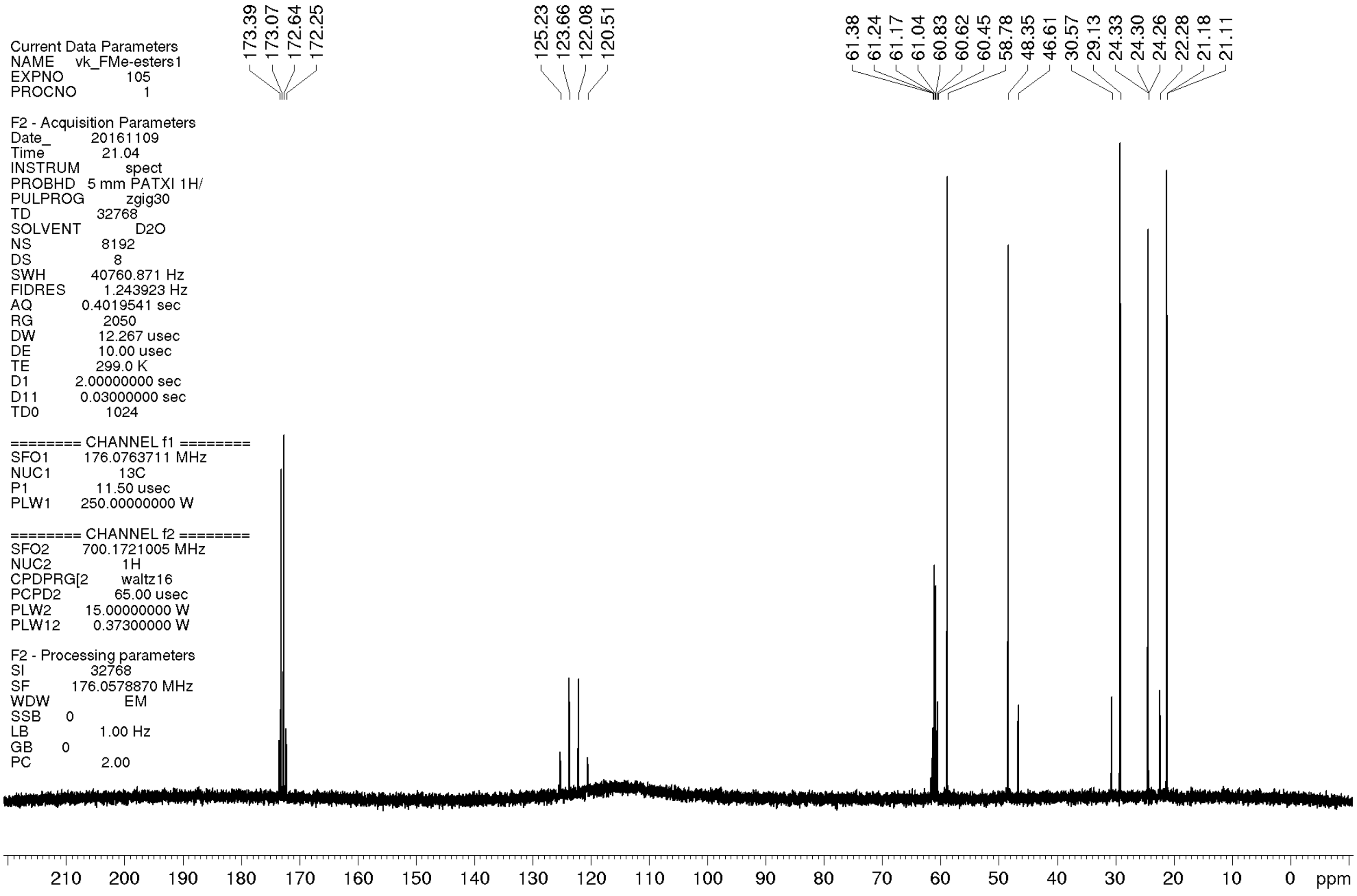

^{19}F and $^{19}F\{^1H\}$ NMR spectra of **5** in deuterium oxide at 471 MHz

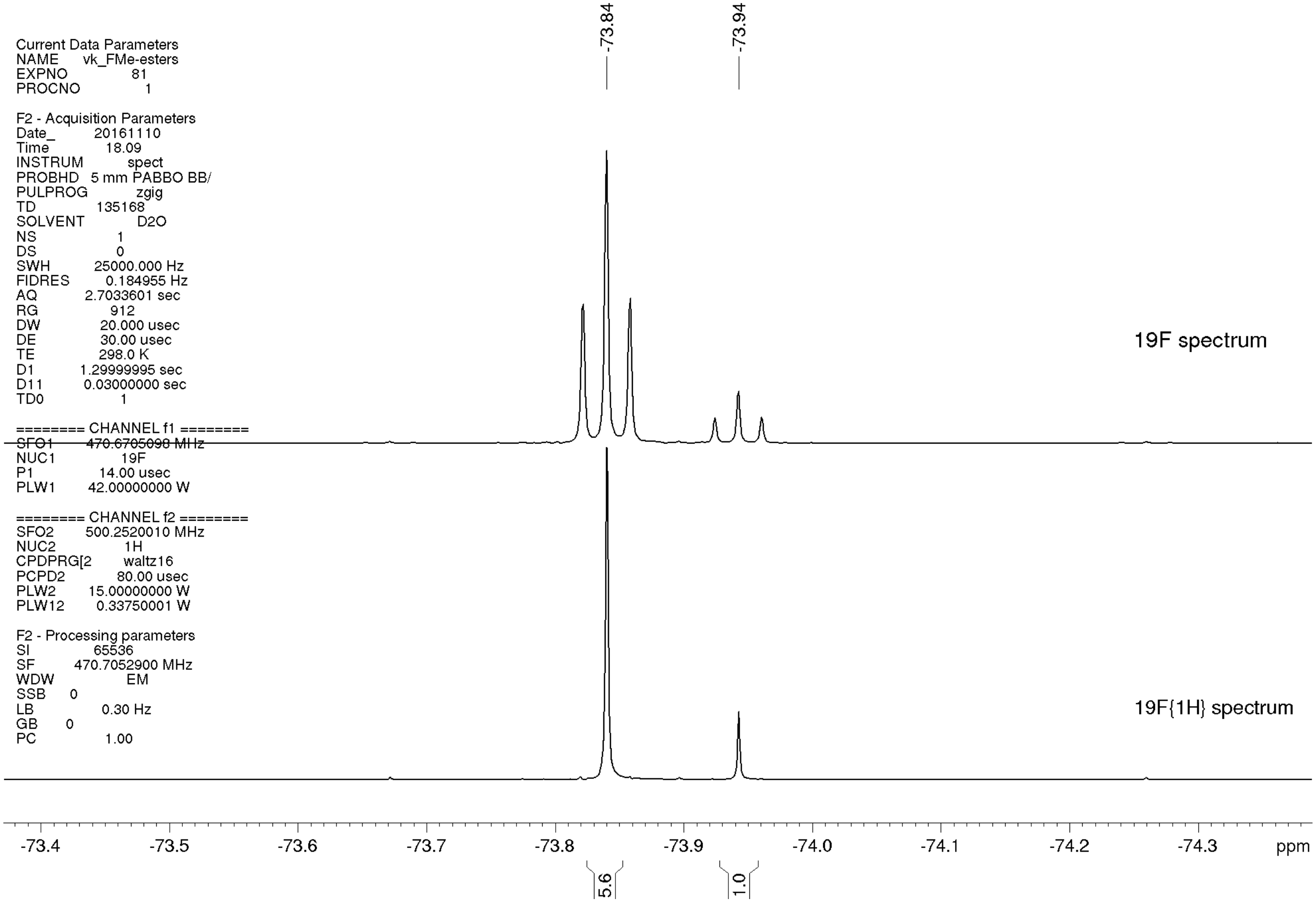

^{1}H NMR spectrum of **5** in benzene-d_6 at 700 MHz

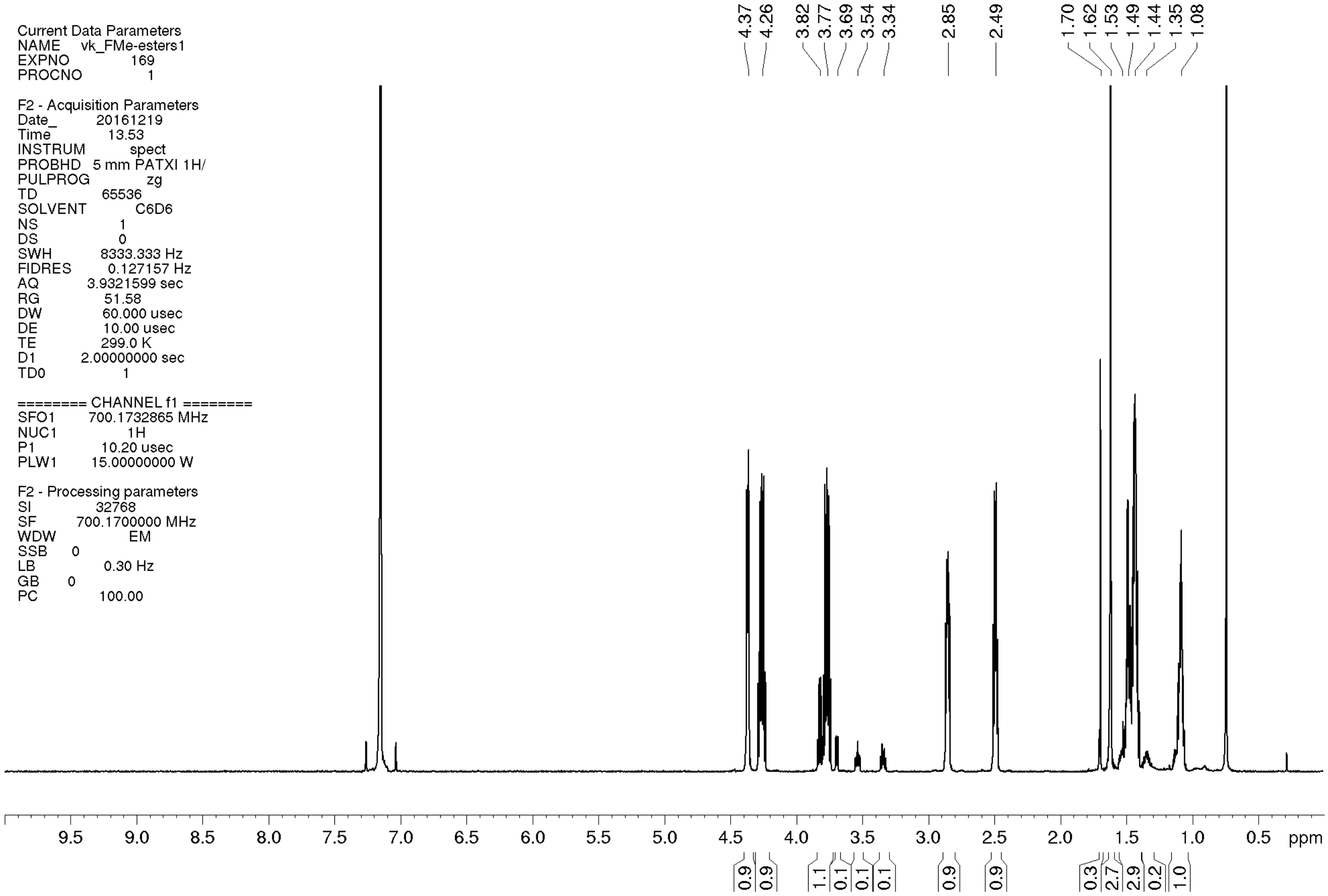

$^{13}C\{^{1}H\}$ NMR spectrum of **5** in benzene-d_6 at 176 MHz

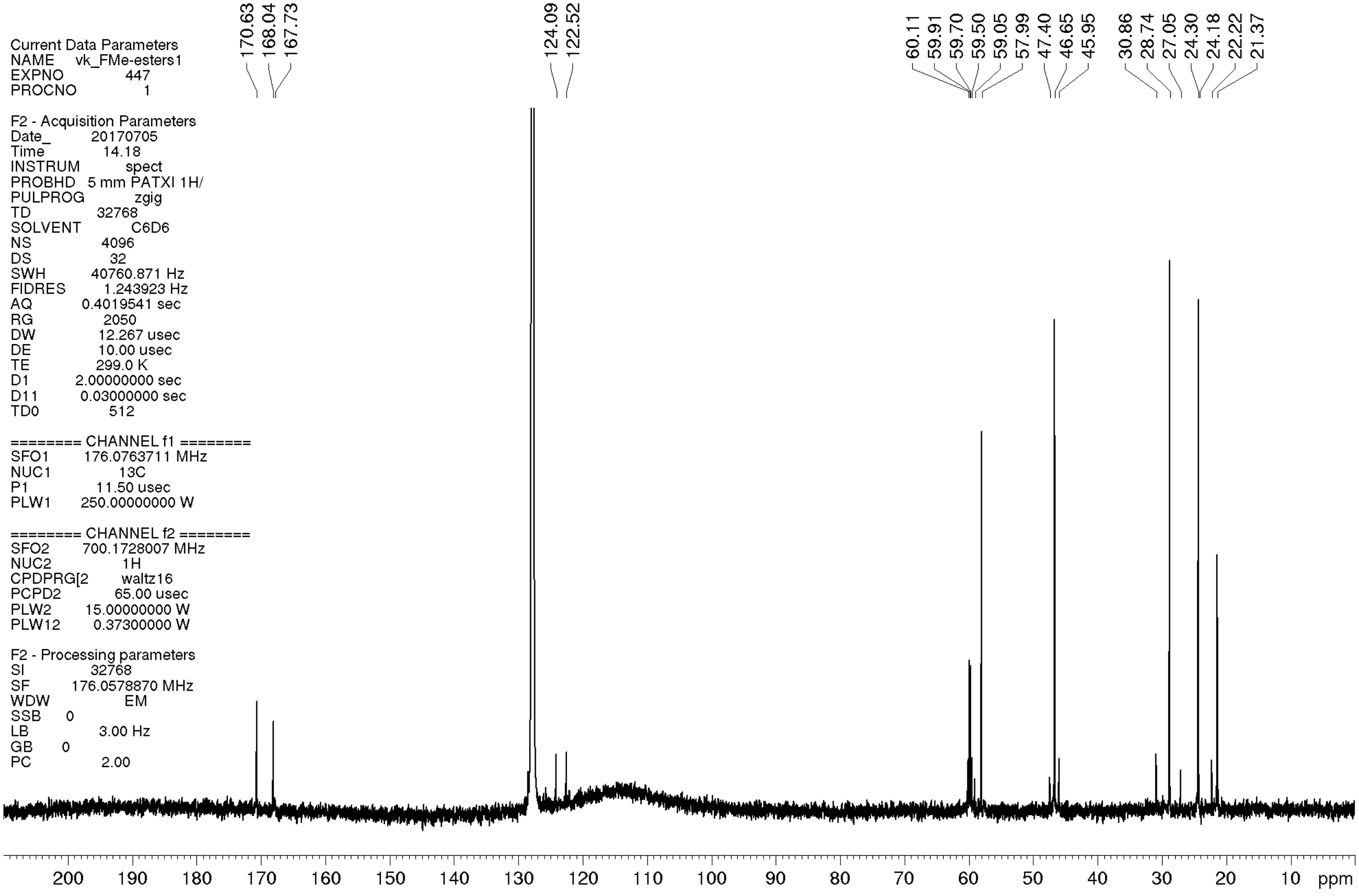

Spectra of **5** (in methanol-d$_4$, 700 MHz) obtained after esterification via chloranhydride (bottom) and in acidic trifluoroethanol (top)

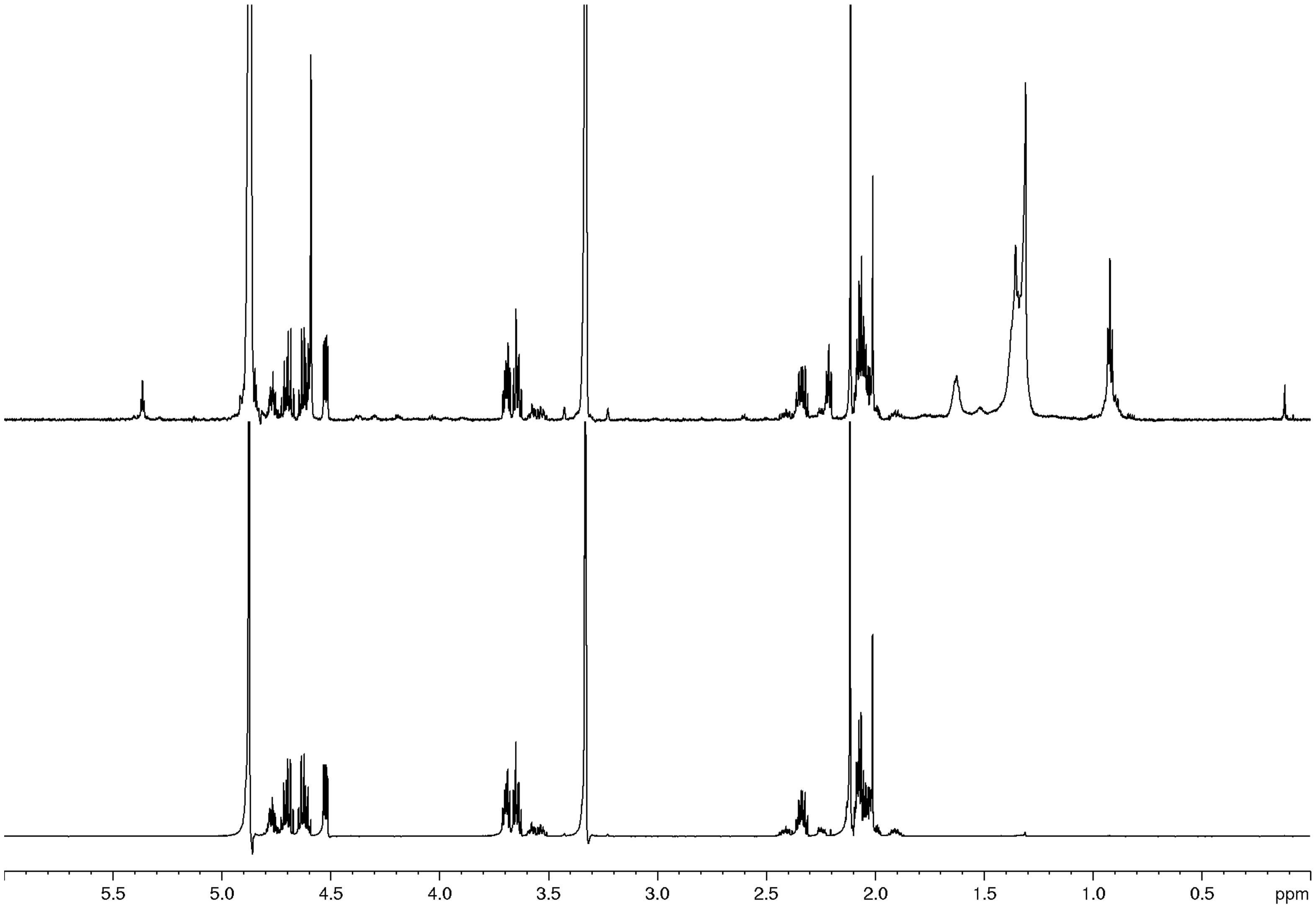

^{1}H NMR spectrum of compound 7

^{1}H NMR spectrum of compound **7** in deuterium oxide at 700 MHz

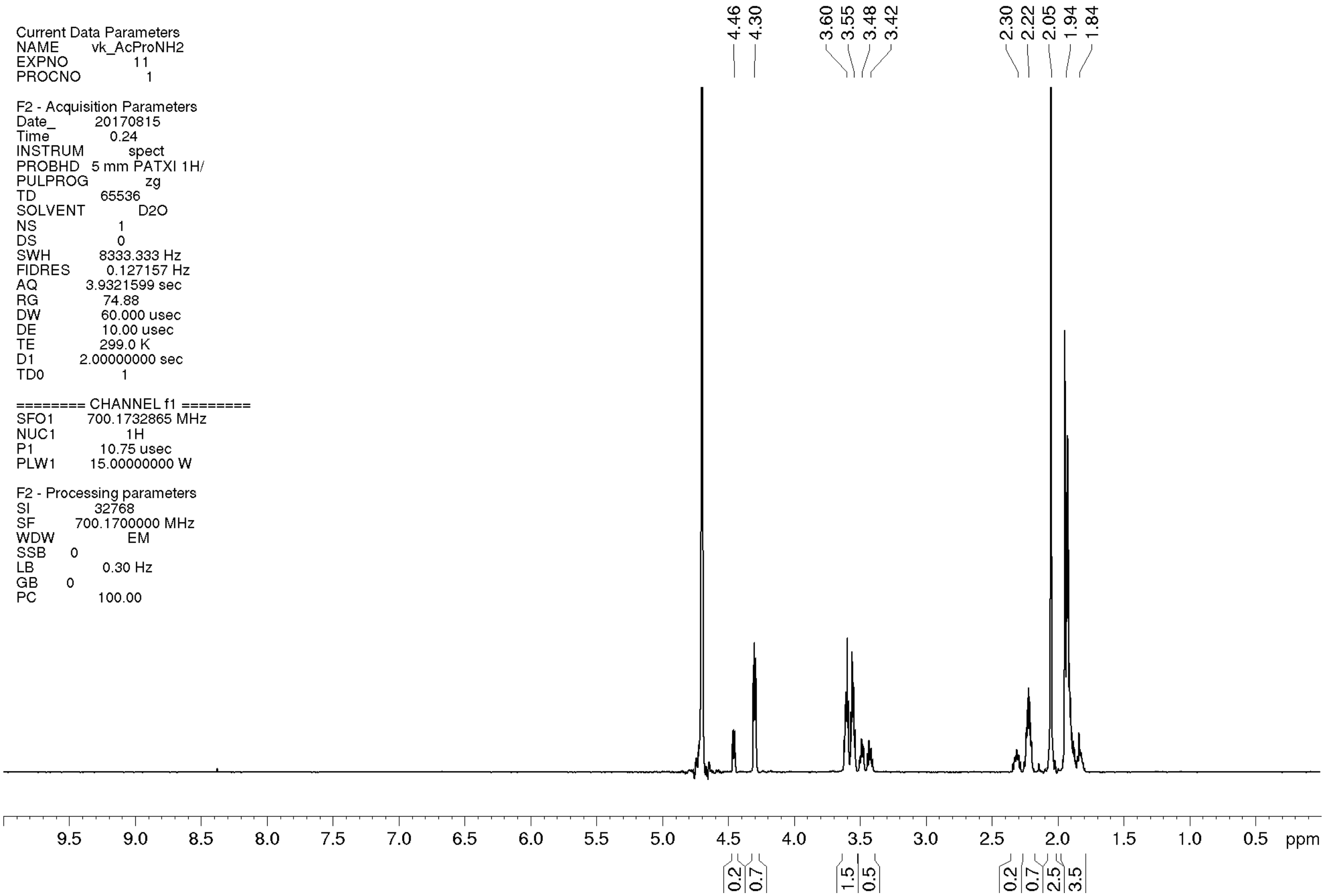

NMR spectra of 3-5 with the europium shift reagent

$^{19}F\{^{1}H\}$ NMR spectra (inverse-gated decoupling) of **3** in dichloromethane-d_2 upon addition of Eu^{3+} shifting reagent (two enantiomers)

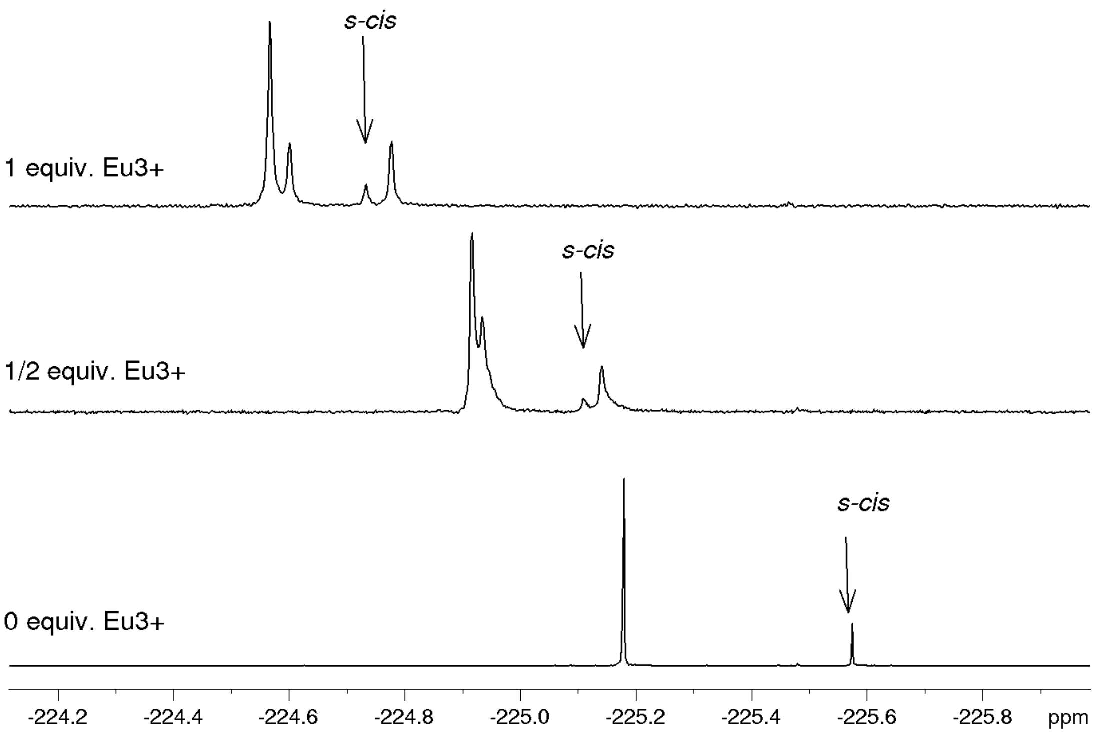

$^{19}F\{^1H\}$ NMR spectra (inverse-gated decoupling) of **4** in dichloromethane-d_2 upon addition of Eu^{3+} shifting reagent (single enantiomer)

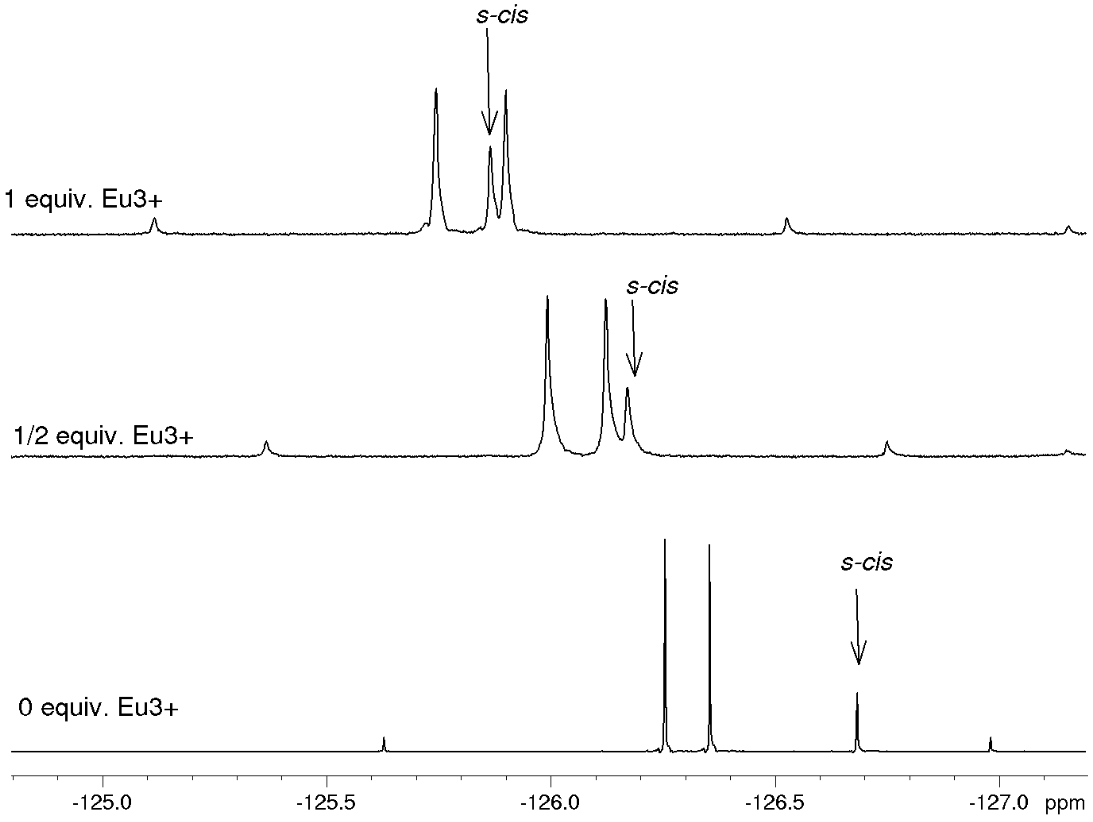

$^{19}F\{^1H\}$ NMR spectra (inverse-gated decoupling) of **5** in dichloromethane-d_2 upon addition of Eu^{3+} shifting reagent (two enantiomers)

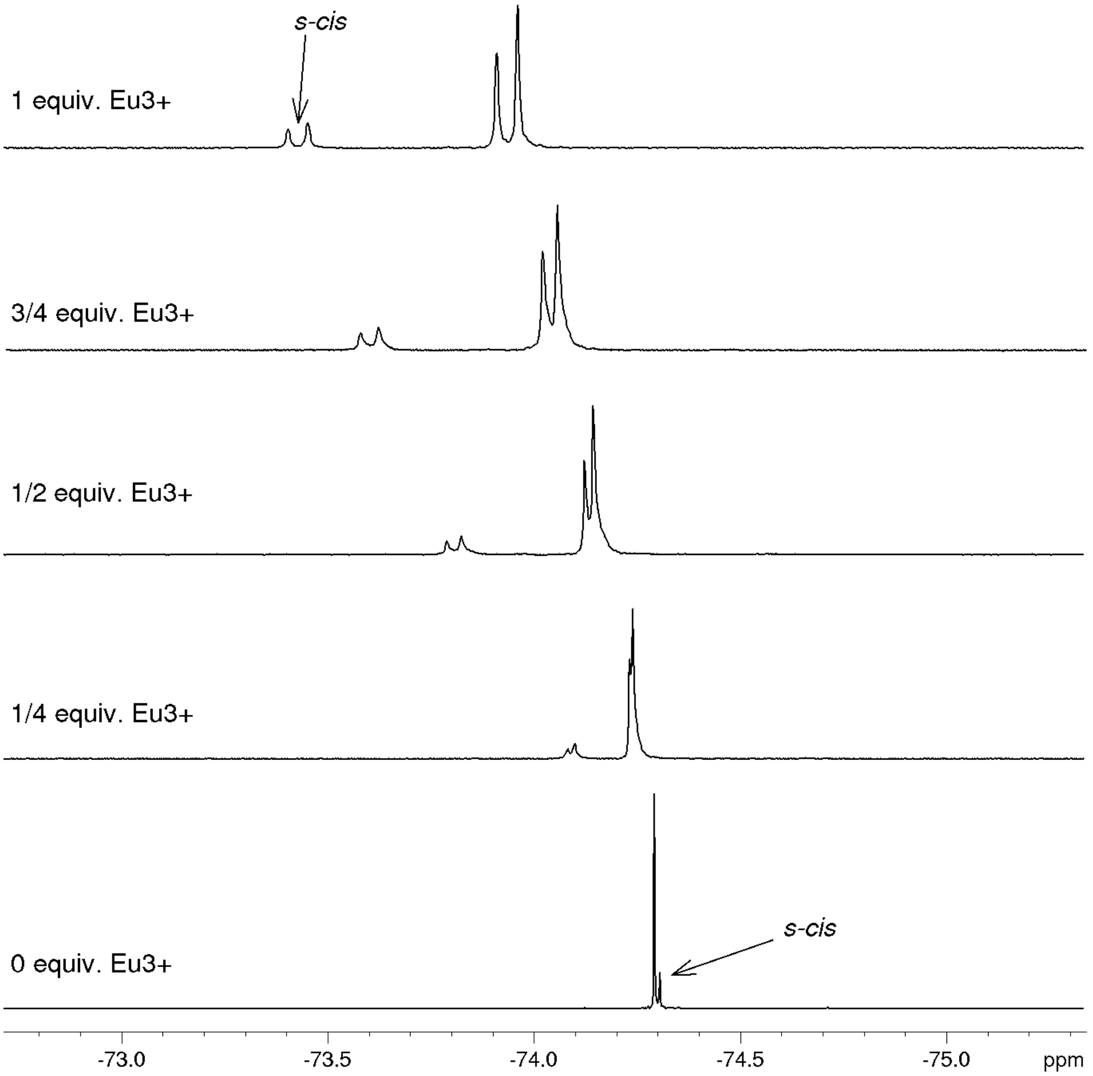

NMR spectra of the peptides

^{1}H 90-pulse NMR spectra in deuterium oxide

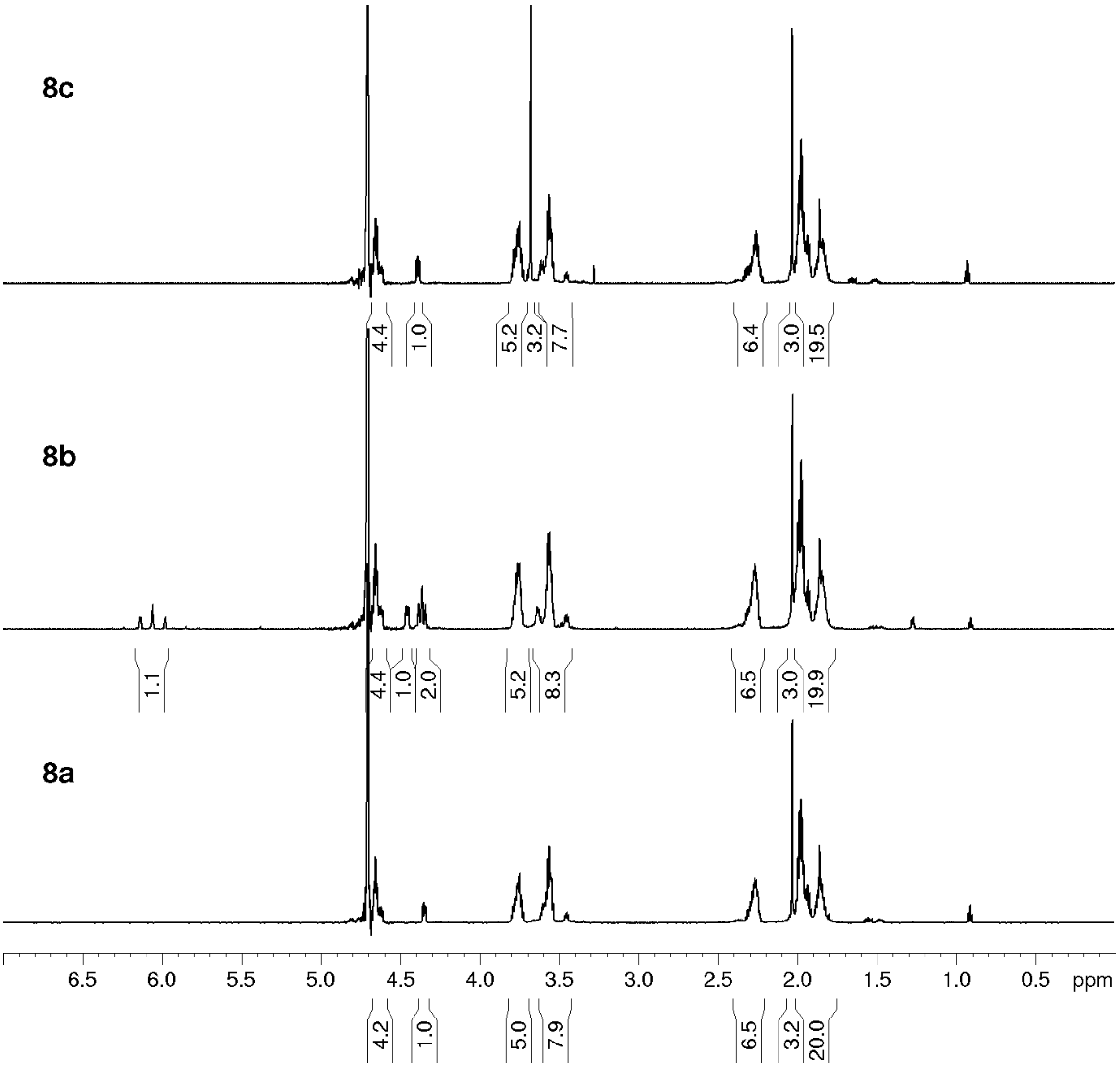

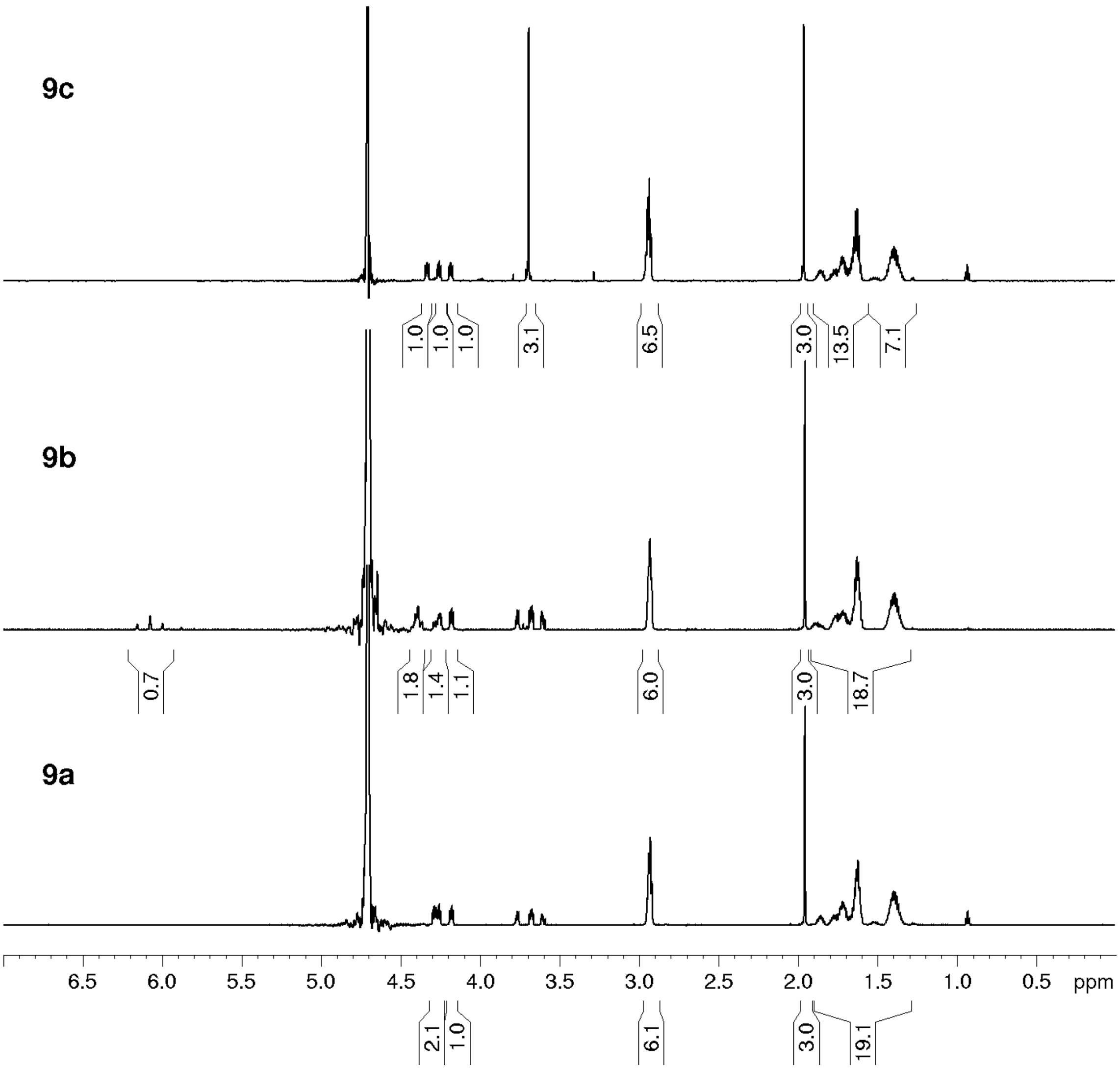
9c
1.0
1.0
1.0
3.1
6.5
3.0
13.5
7.1
9b
0.7
1.8
1.4
1.1
6.0
3.0
18.7
9a
6.5
6.0
5.5
5.0
4.5
4.0
3.5
3.0
2.5
2.0
1.5
1.0
0.5
ppm
2.1
1.0
6.1
3.0
19.1

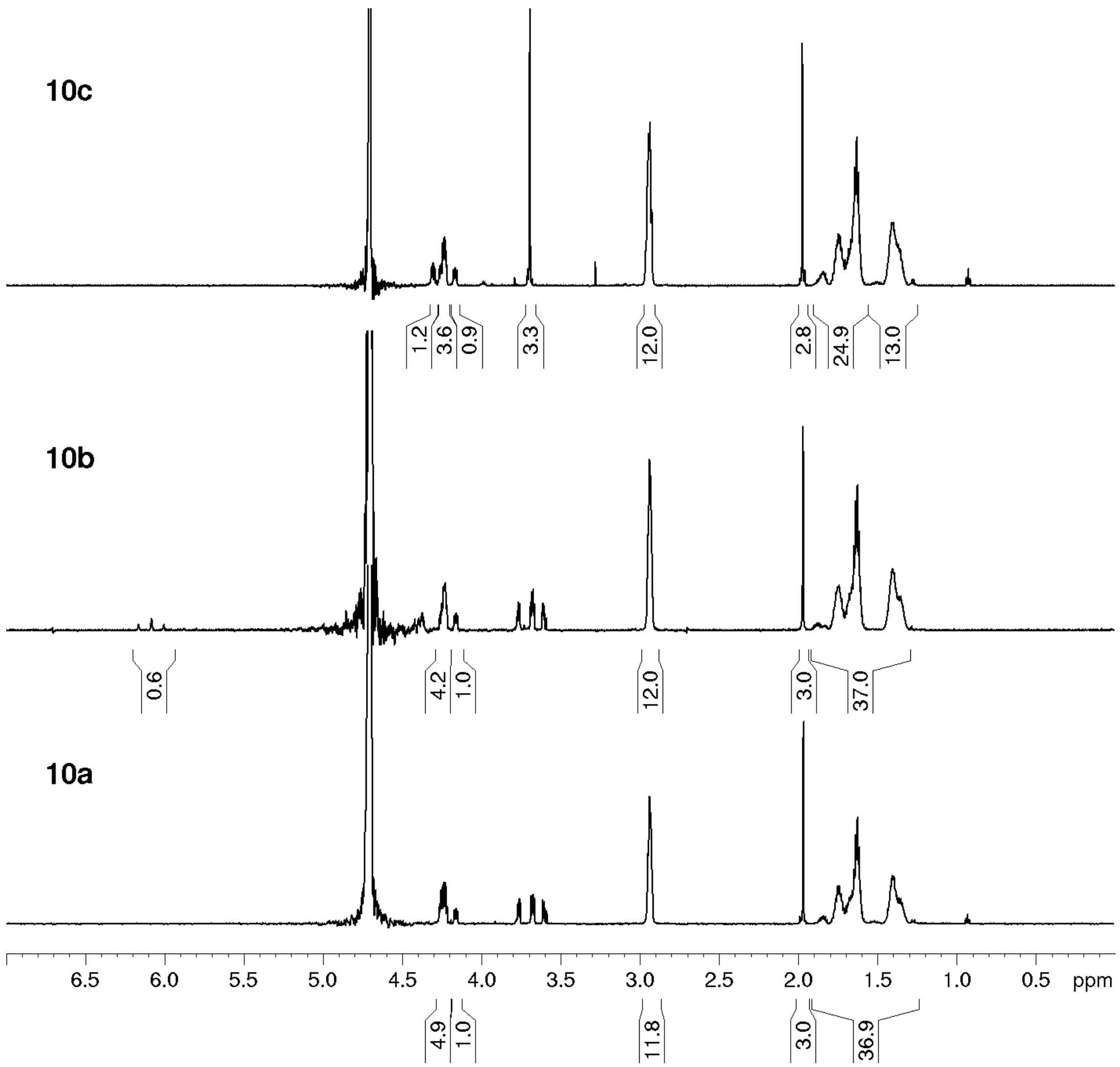
10c
10b
10a
1.2
3.6
0.9
3.3
12.0
2.8
24.9
13.0
0.6
4.2
1.0
12.0
3.0
37.0
4.9
1.0
11.8
3.0
36.9
6.5 6.0 5.5 5.0 4.5 4.0 3.5 3.0 2.5 2.0 1.5 1.0 0.5 ppm

^{1}H 1D stimulated echo NMR spectra in deuterium oxide at 700 MHz

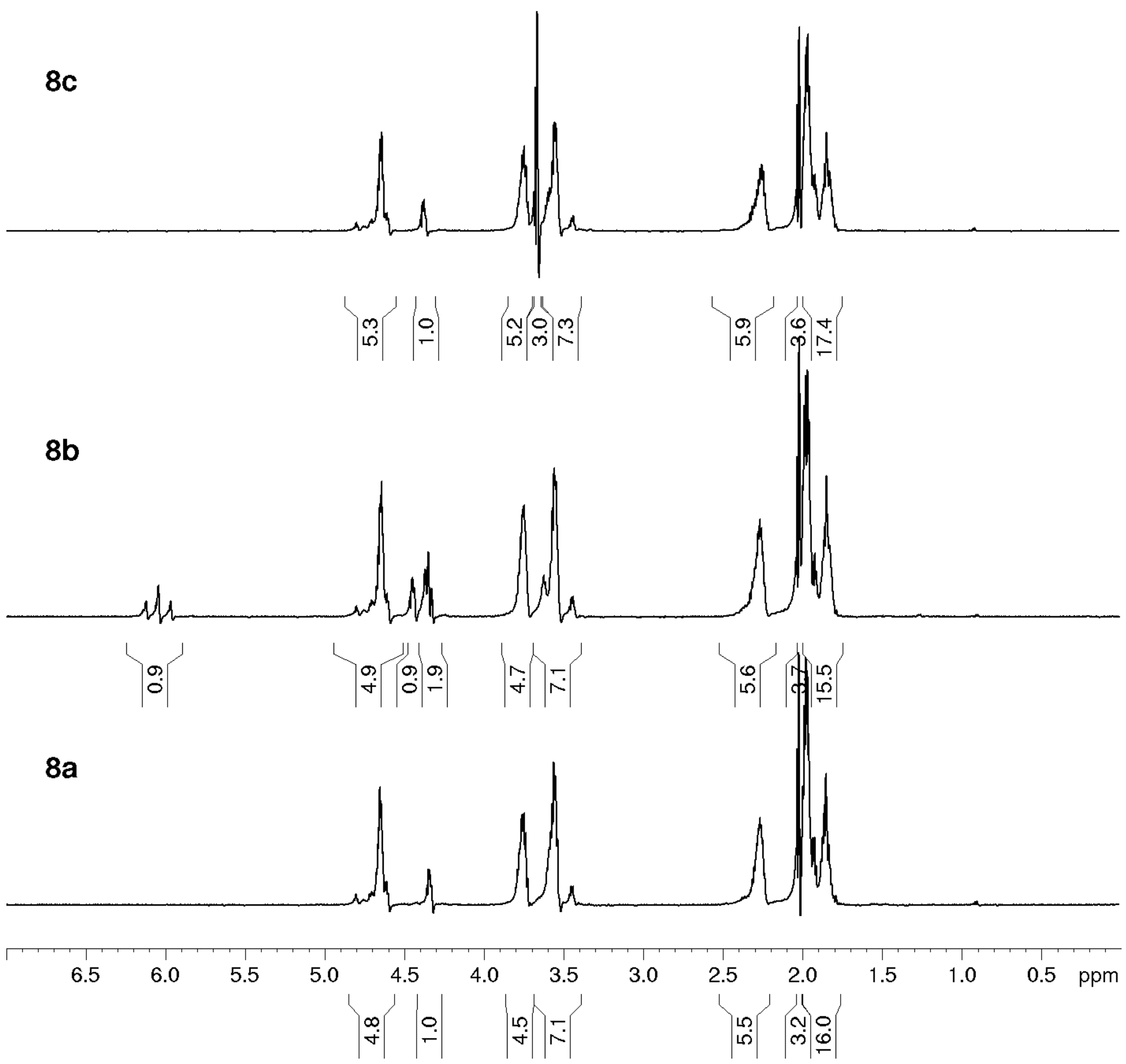

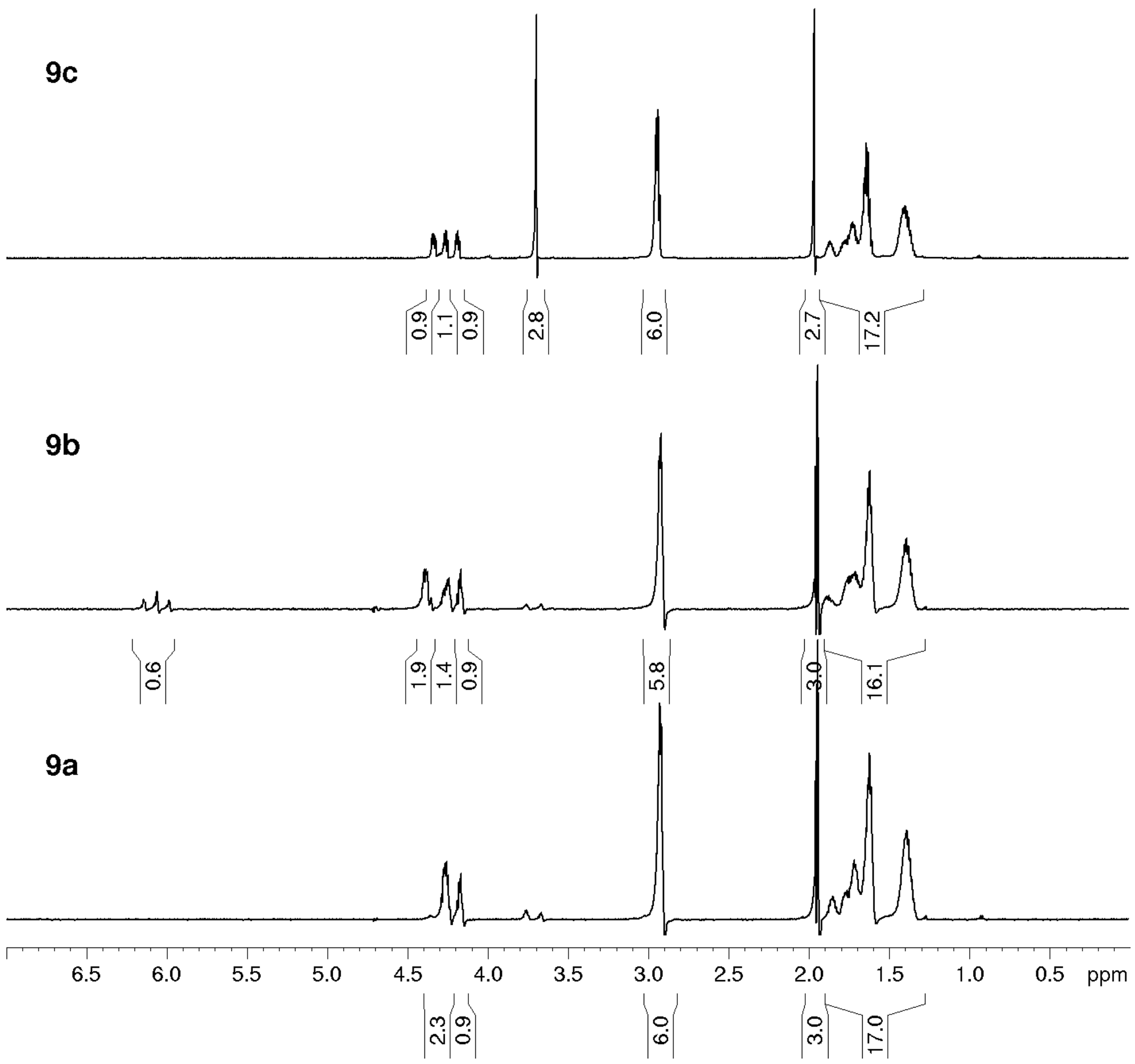
9c
0.9
1.1
0.9
2.8
6.0
2.7
17.2
9b
0.6
1.9
1.4
0.9
5.8
3.0
16.1
9a
6.5
6.0
5.5
5.0
4.5
4.0
3.5
3.0
2.5
2.0
1.5
1.0
0.5
ppm
2.3
0.9
6.0
3.0
17.0

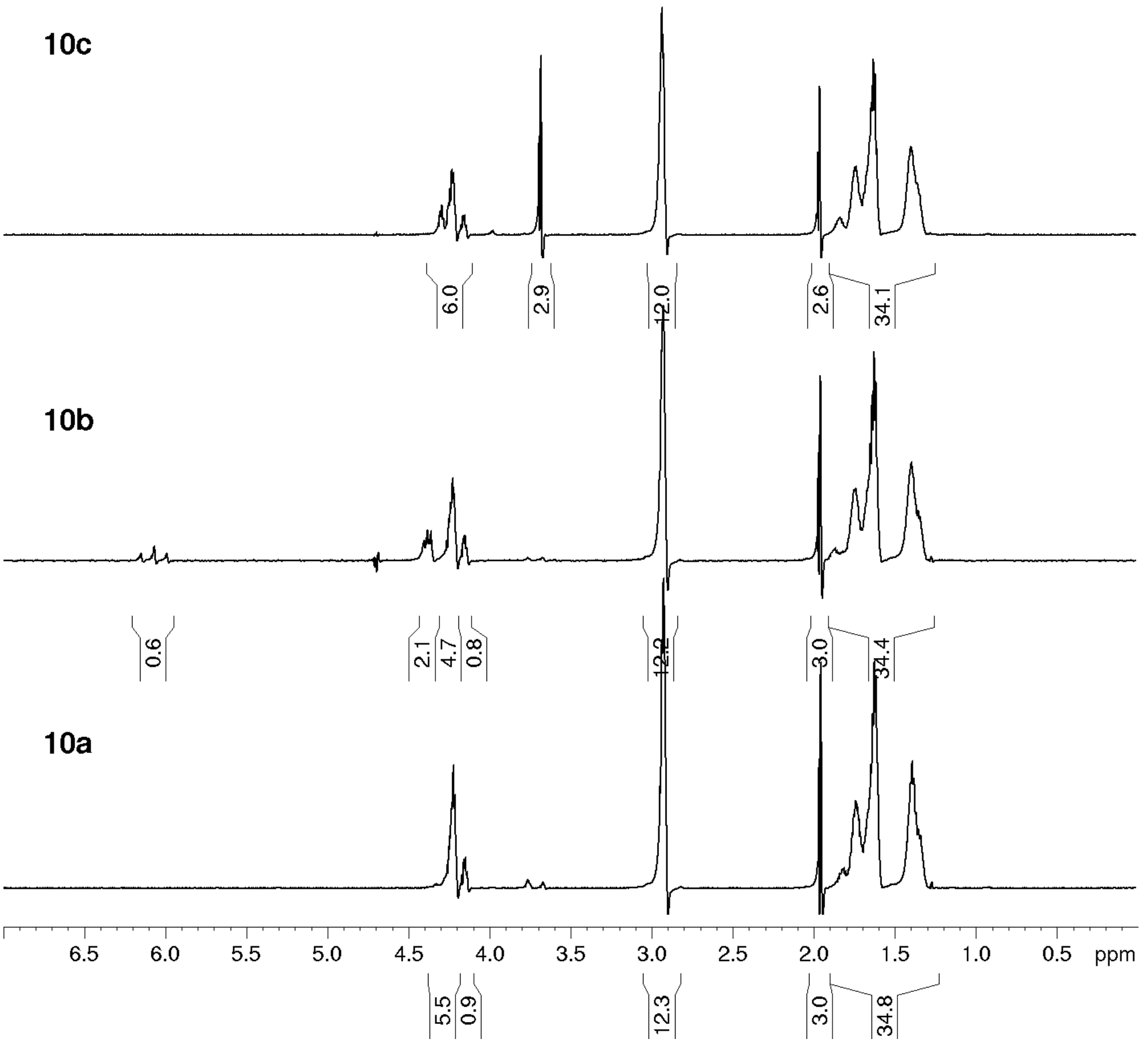
10c
6.0
2.9
12.0
2.6
34.1
10b
0.6
2.1
4.7
0.8
12.2
3.0
34.4
10a
6.5
6.0
5.5
5.0
4.5
4.0
3.5
3.0
2.5
2.0
1.5
1.0
0.5
ppm
5.5
0.9
12.3
3.0
34.8

^{19}F NMR spectra of the peptides in deuterium oxide at 471 MHz

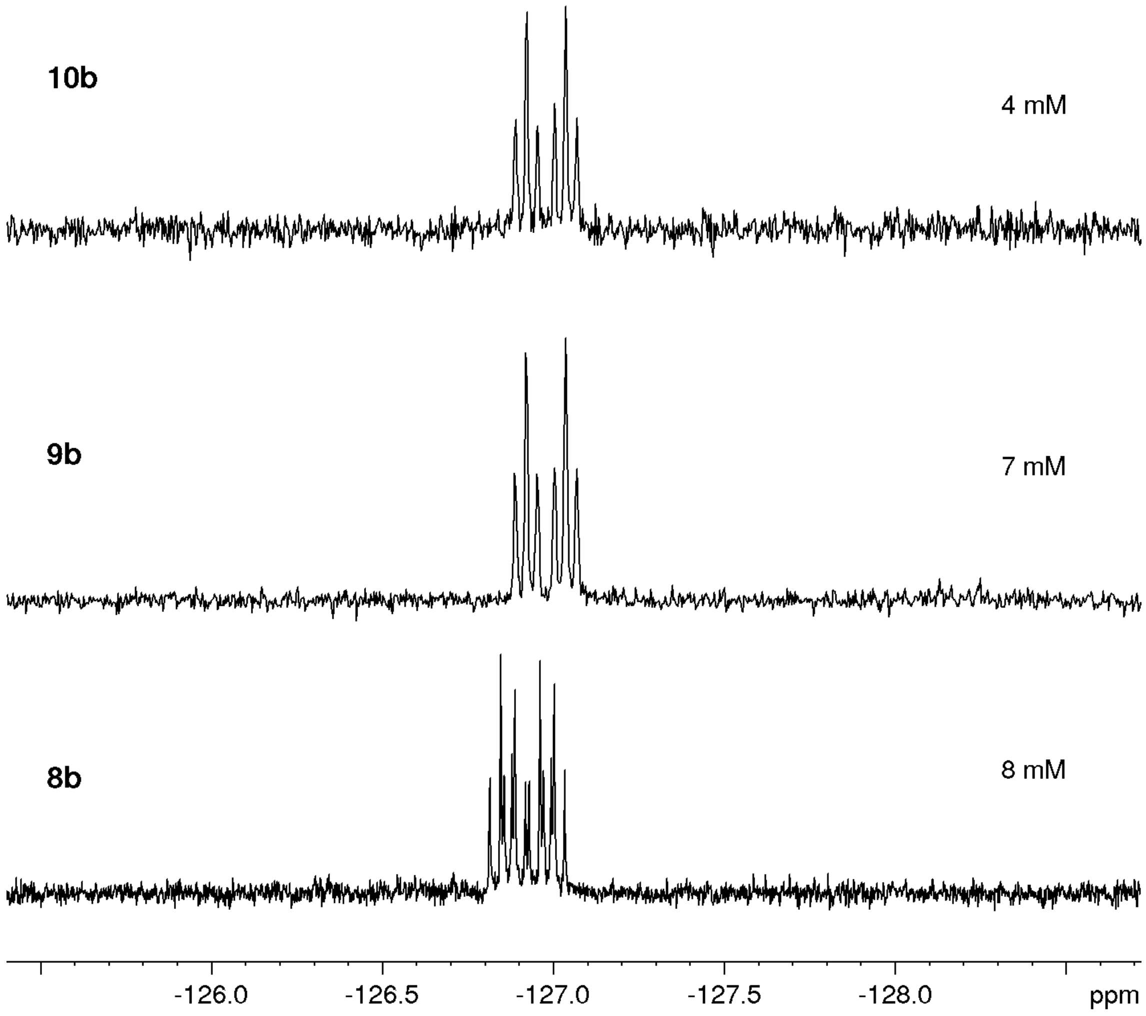

Circular dichroism spectra for the peptides

in methanol:

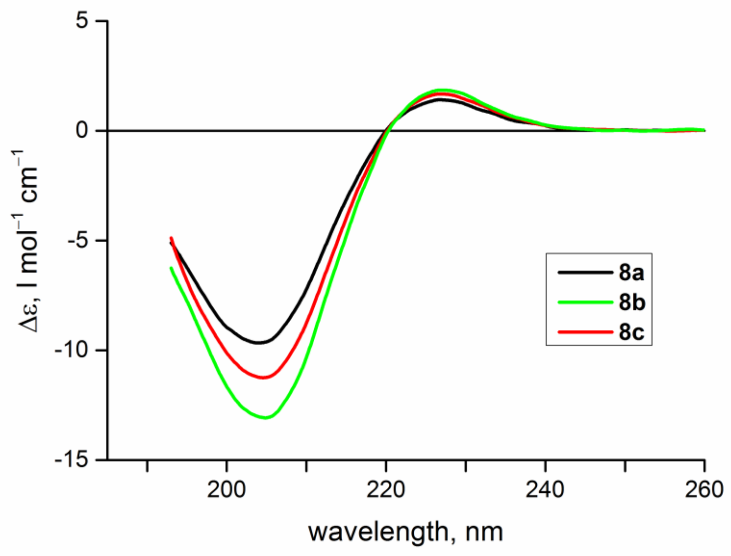

in aqueous buffer:

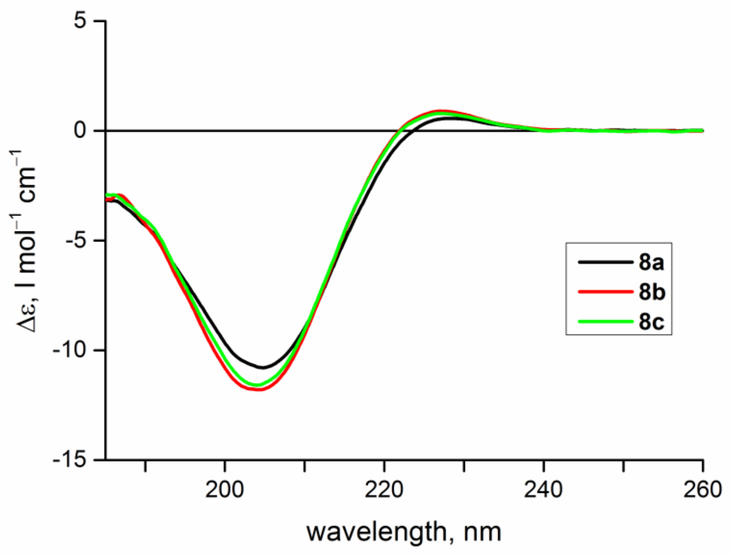

in methanol:

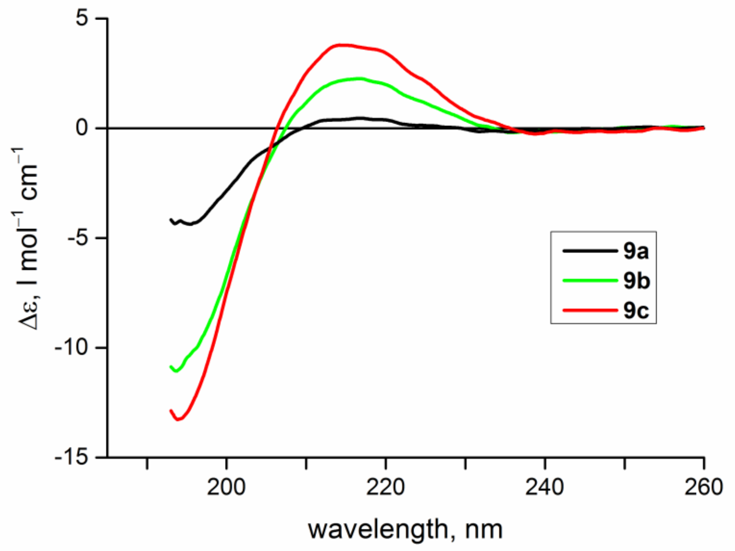

in aqueous buffer:

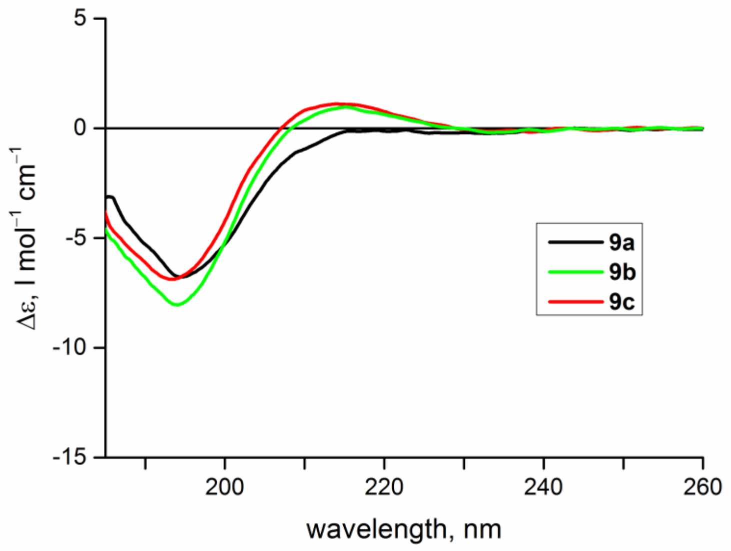

in methanol:

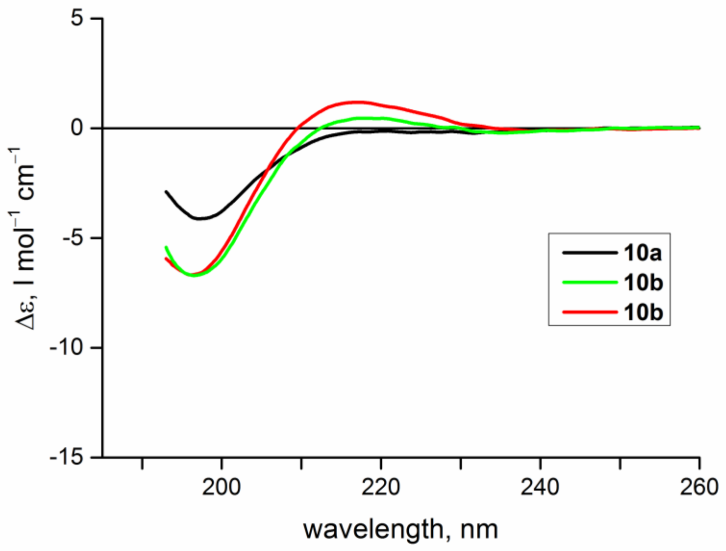

in aqueous buffer:

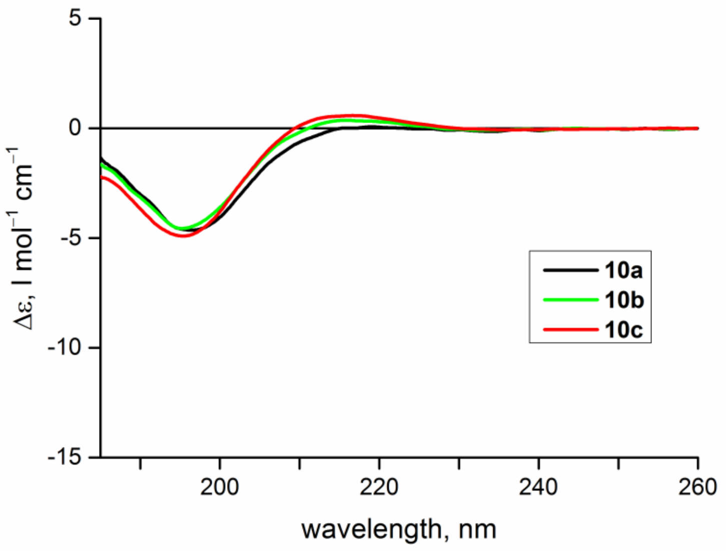

Hydrolysis of the peptides

Starting peptide concentrations **8b** – 5 mM, **9b** – 5 mM, **10b** – 2.5 mM

peptide **8b**, series # 1

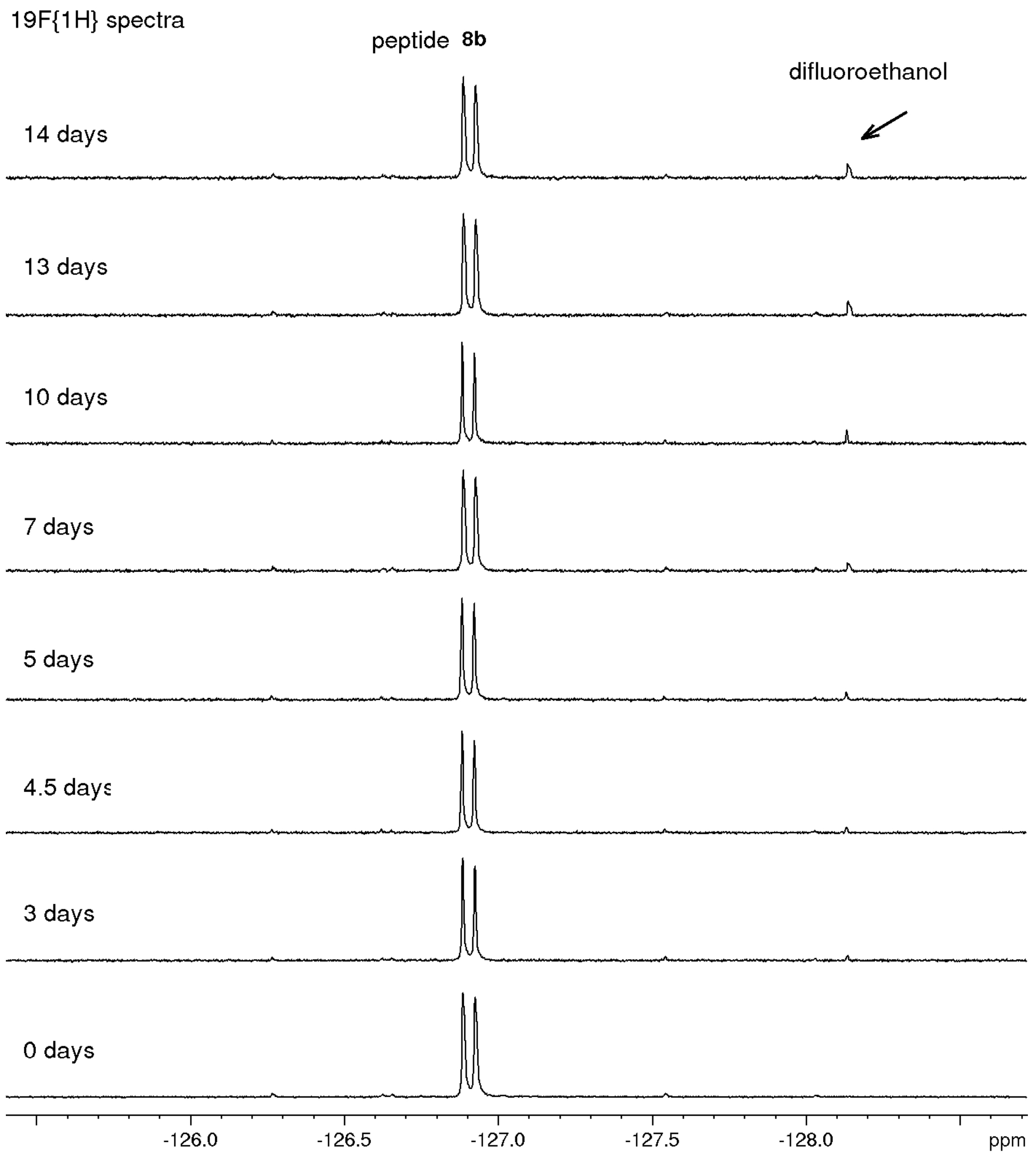

peptide **9b**, series # 1

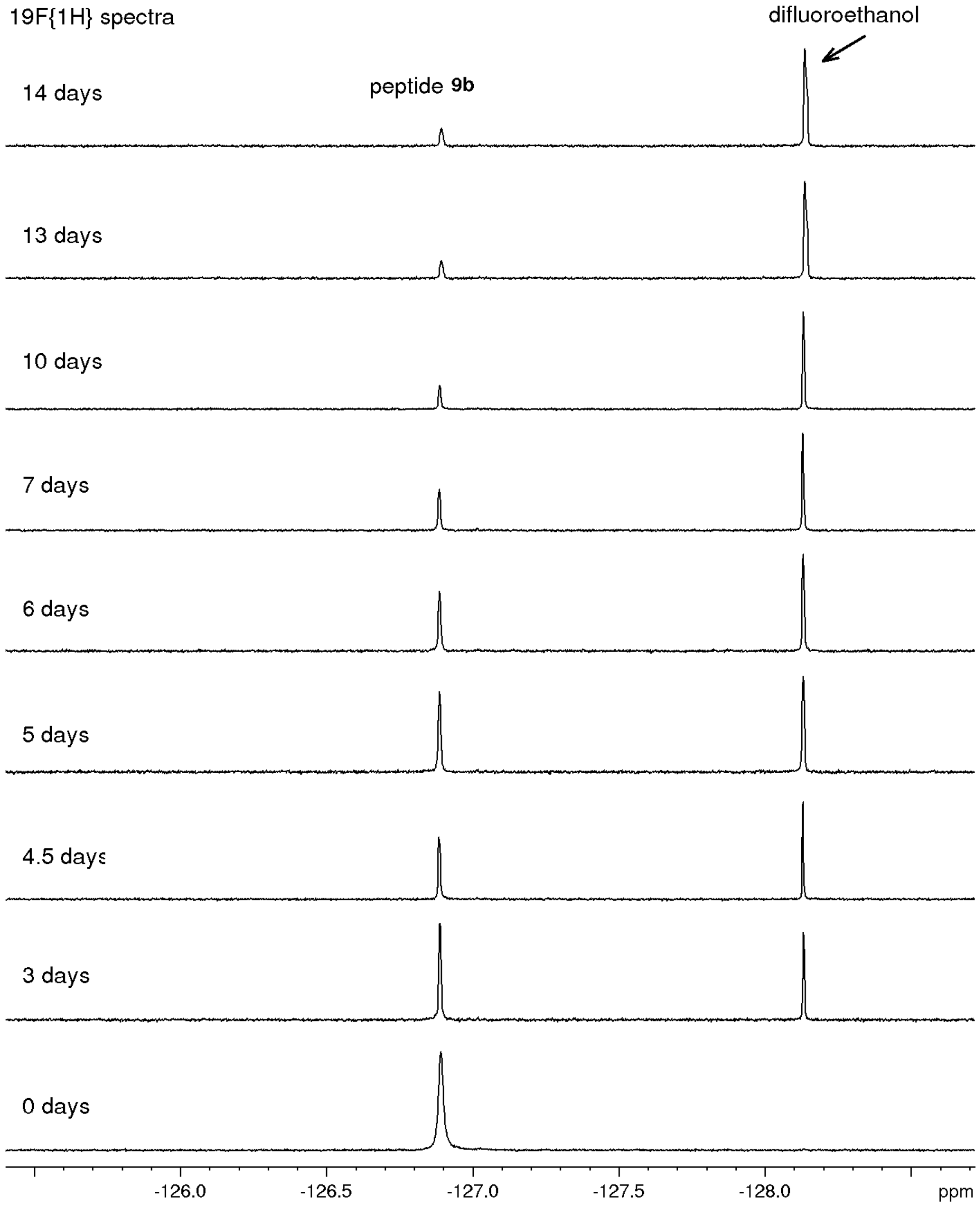

peptide **10b**, series # 1

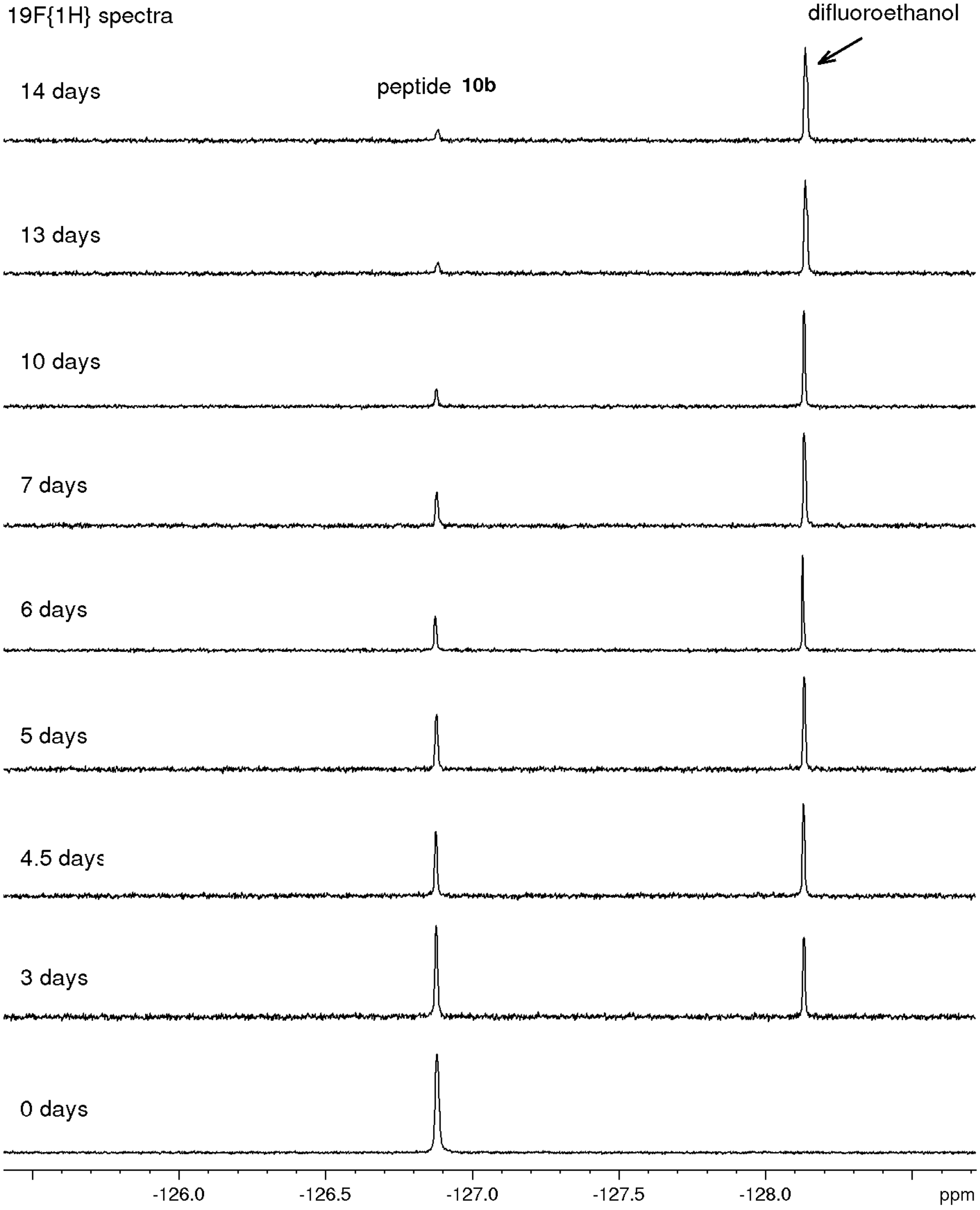

peptide **9b**, series # 2

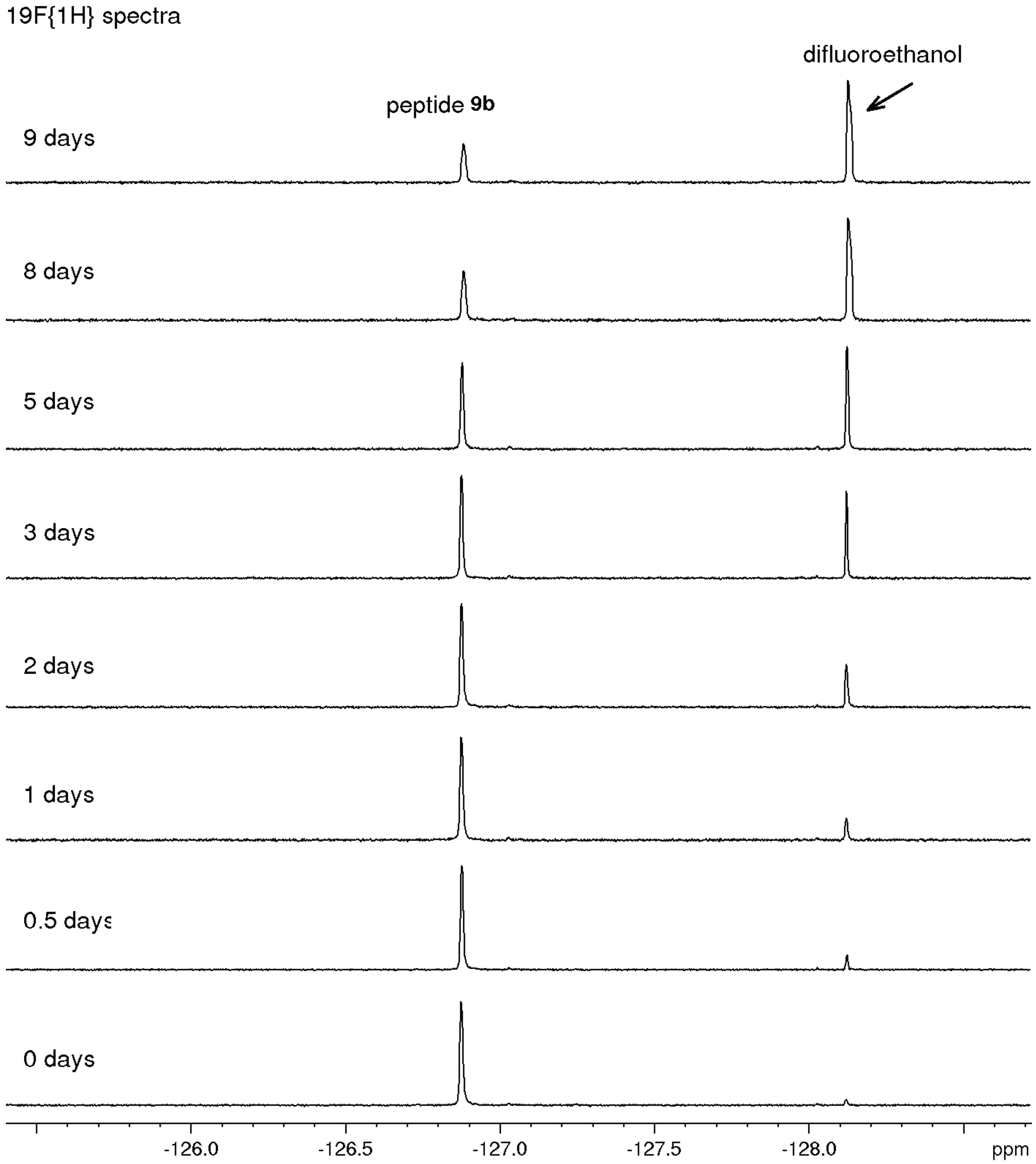

peptide **10b**, series # 2

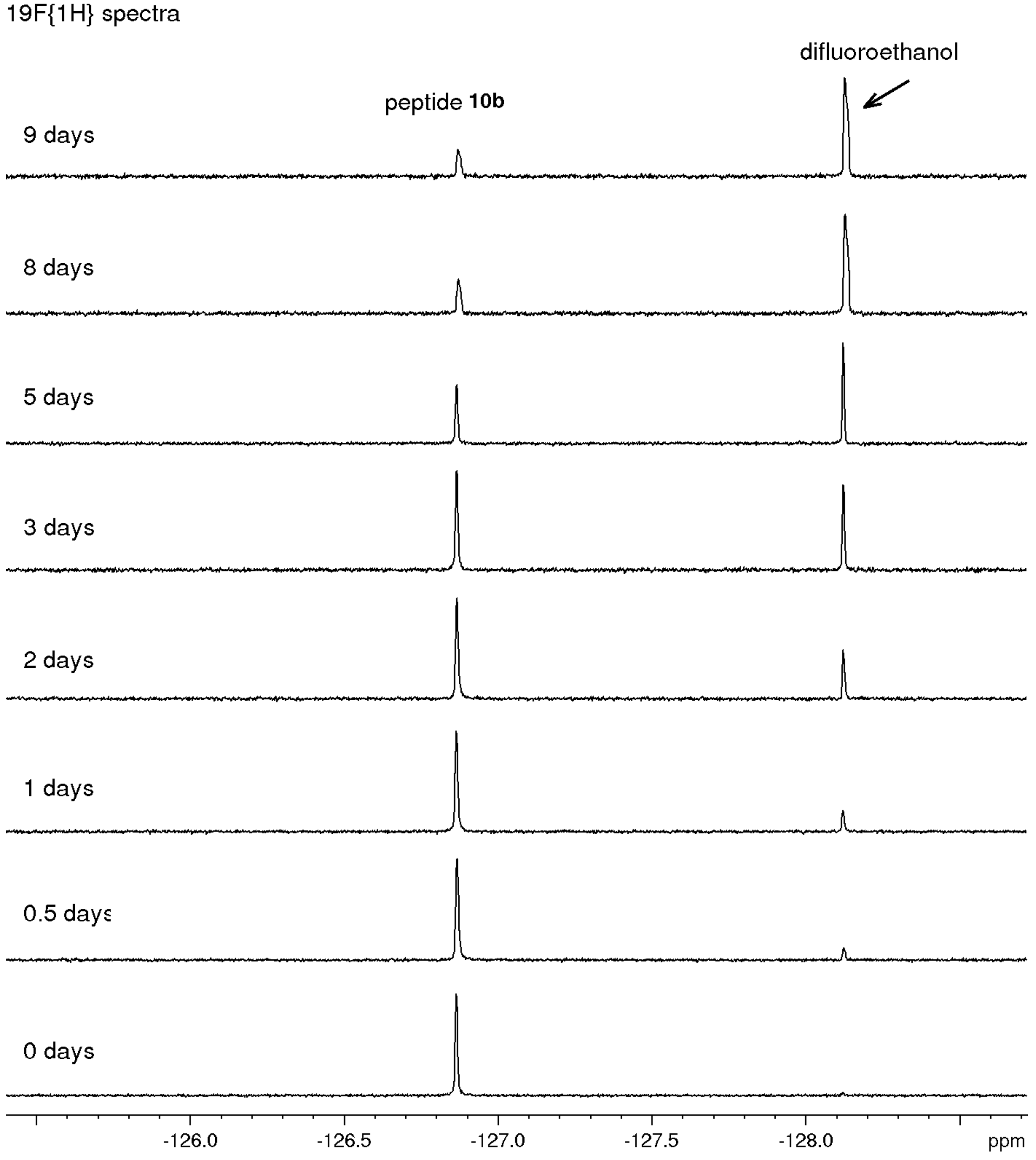

Supplement: File 1 — Amide equilibrium constants (Table S1) and copies of the NMR and CD spectra. [file Beilstein_J_Org_Chem-13-2442-s001.pdf]
